# The AuTOMATIC trial: a study protocol for a multi-arm Bayesian adaptive randomised controlled trial of text messaging to improve childhood immunisation coverage

**DOI:** 10.1186/s13063-023-07097-3

**Published:** 2023-02-07

**Authors:** Grace E. Currie, James Totterdell, Grahame Bowland, Alan Leeb, Ian Peters, Chris C. Blyth, Claire Waddington, Julie A. Marsh, Thomas L. Snelling

**Affiliations:** 1 Wesfarmers Centre of Vaccines and Infectious Diseases, Telethon Kids Institute, Perth, WA Australia; 2 School of Public Health, Faculty of Medicine and Health, University of Sydney, Sydney, NSW 2006 Australia; 3 SmartVax, c/o Illawarra Medical Centre, Perth, WA Australia; 4 Department of Infectious Diseases, Perth Children’s Hospital, Perth, WA Australia; 5 School of Medicine, University of Western Australia, Perth, WA Australia; 6 Department of Medicine, University of Cambridge, Cambridge, UK; 7 School of Public Health, Curtin University, Perth, WA Australia; 8 Menzies School of Public Health, Darwin, NT Australia

**Keywords:** Vaccination, Childhood immunisation, SMS, Text messaging, mHealth, Reminder systems

## Abstract

**Background:**

While most Australian children are vaccinated, delays in vaccination can put them at risk from preventable infections. Widespread mobile phone ownership in Australia could allow automated short message service (SMS) reminders to be used as a low-cost strategy to effectively ‘nudge’ parents towards vaccinating their children on time.

**Methods:**

AuTOMATIC is an adaptive randomised trial which aims to both evaluate and optimise the use of SMS reminders for improving the timely vaccination of children at primary care clinics across Australia. The trial will utilise high levels of digital automation to effect, including eligibility assessment, randomisation, delivery of intervention, data extraction and analysis, thereby allowing healthcare-embedded trial delivery. Up to 10,000 parents attending participating primary care clinics will be randomised to one of 12 different active SMS vaccine reminder content and timing arms or usual practice only (no SMS reminder). The primary outcome is vaccine receipt within 28 days of the scheduled date for the index vaccine (the first scheduled vaccine after randomisation). Secondary analyses will assess receipt and timeliness for all vaccine occasions in all children. Regular scheduled analyses will be performed using Bayesian inference and pre-specified trial decision rules, enabling response adaptive randomisation, suspension of any poorly performing arms and early stopping if a single best message is identified.

**Discussion:**

This study will aim to optimise SMS reminders for childhood vaccination in primary care clinics, directly comparing alternative message framing and message timing. We anticipate that the trial will be an exemplar in using Bayesian adaptive methodology to assess a readily implementable strategy in a wide population, capable of delivery due to the levels of digital automation. Methods and findings from this study will help to inform strategies for implementing reminders and embedding analytics in primary health care settings.

**Trial registration:**

ANZCTR: ACTRN12618000789268.

**Supplementary Information:**

The online version contains supplementary material available at 10.1186/s13063-023-07097-3.

## Administrative information

Note: the numbers in curly brackets in this protocol refer to SPIRIT checklist item numbers. The order of the items has been modified to group similar items (see http://www.equator-network.org/reporting-guidelines/spirit-2013-statement-defining-standard-protocol-items-for-clinical-trials/).

**Table:** 

Title {1}	Full title: A multi-arm Bayesian adaptive randomised controlled trial of text messaging to improve childhood immunisation coverage Trial acronym: AuTOMATIC
Trial registration {2a and 2b}.	ACTRN12618000789268. Registered on 10/05/2018.
Protocol version {3}	V2.0 Oct 2020
Funding {4}	Ramaciotti Foundation and Royal Australasian College of Physicians The funder has approved the study plan for funding administered via Curtin University, but will have no role in the collection, management, analysis, interpretation, reporting or decision to submit for publication which rests entirely with the project team.
Author details {5a}	Ms Grace Currie, Telethon Kids Institute and University of Sydney Mr James Totterdell, Telethon Kids Institute and University of Sydney Mr Grahame Bowland, Telethon Kids Institute Dr Alan Leeb, SmartVax Mr Ian Peters, SmartVax Prof Chris Blyth, Telethon Kids Institute and University of Western Australia Dr Claire Waddington, University of Cambridge Dr Julie Marsh, Telethon Kids Institute Prof Tom Snelling, Telethon Kids Institute and University of Sydney
Name and contact information for the trial sponsor {5b}	Telethon Kids Institute 69 Hospital Ave, Nedlands WA 6009 ResearchGovernance@telethonkids.org.au
Role of sponsor {5c}	The sponsor has overall responsibility over the conduct of this study ensuring it is performed in line with all regulatory standards.
Ethics Committee	University of Western Australia Human Research Ethics Committee (Ref: 2019/RA/4/1/8810)

### Background and rationale {6a}

Vaccination is a highly effective preventative strategy of improving population-level child health [1], but its efficacy is undermined by under-vaccination (not receiving all vaccines for which a person is eligible) and delayed vaccination (not receiving vaccines at the age or time recommended). Reasons for under-vaccination are complex and vary between people; our previous research has shown that practical barriers, such as ease of access to appointments and remembering appointments, may be more important determinants of under-vaccination in Australia than concerns about vaccine safety or doubts about necessity or efficacy [2]. Furthermore, the relative intensity and complexity of the vaccine schedule in early childhood that now covers a wide range of vaccine preventable diseases may present a competing demand to busy families, with some parents simply unaware that a vaccine dose has become due [3, 4].

A low number of small to moderate scale randomised control trials (RCTs) and observational studies have assessed the impact of SMS-based interventions on vaccine uptake and timeliness in childhood vaccination. Most of these studies have assessed impact in specific at-risk populations including lower socioeconomic service areas. Evidence from these studies suggests that SMS reminders can induce small to moderate improvements in vaccination rates and timeliness compared to no SMS (alternative reminders) [5–10] and no reminders [9–11]. Some studies reported greater improvements in vaccine coverage when the reminder included embedded educational messages [6, 8, 10, 12].

The COVID-19 pandemic has highlighted the importance of consistent public health messaging strategies for promoting vaccination. Whether the effectiveness of SMS reminders is influenced by (1) their timing of delivery in relation to the due date or (2) can be improved by framing messages to target potential health benefits, the risks of disease or the social motivators for vaccination, requires evaluation among a large representative population. SMS reminders for routine childhood vaccination have also not been evaluated in an Australian context, leading to uncertainty on how and when to implement this intervention effectively in primary care. Adaptive trial methods and automation of clinical trial processes may provide opportunity to enable these large-scale evaluations in primary care.

## Aim

We aim to evaluate and optimise the use of personalised, provider-initiated SMS reminders for improving the timeliness of routine vaccination among Australian children.

### Objectives {7} and outcomes {12}

#### Primary objective

The primary objective is to determine whether, compared to usual practice, a personalised SMS reminder with specific timing and message framing, will improve the rate of timely vaccination in primary care, that is, the proportion of children vaccinated within 28 days of the scheduled date for a routine childhood vaccine dose.

A child and their siblings may receive more than one scheduled vaccination during the follow-up period; the parent will be the unit of randomisation and will only be randomised once. The parent whose behaviour we seek to change can only be considered naïve to the intervention on the first child-vaccination occasion; therefore, the primary analysis will be performed on the outcome of the first child-vaccination occasion for each parent after randomisation (the *index vaccine occasion* and the *index child*).

#### Primary outcome

The primary outcome is receipt of the index vaccine within 28 days of the vaccine scheduled date for the index child.

#### Secondary objectives

The secondary objectives are to determine whether, compared to usual care, a personalised parental SMS reminder with specific timing and content framing, will improve timely vaccination (vaccination within 28 days of the scheduled date) and reduce the time to vaccination from the scheduled date, for all age-scheduled vaccines after randomisation for all children (index child and siblings).

#### Secondary outcomes

The secondary outcomes are as follows:

1. The time elapsed from 14 days before the due date to administration of the index vaccine, censored at 42 days after the due date;
2. Vaccination status at 28 days post scheduled vaccine due date (binary variable; vaccinated vs not vaccinated) for all children and all vaccine doses;
3. The time elapsed from 14 days before the due date to administration of any vaccine dose, censored at 42 days after the due date for all children and all vaccination doses.

#### Trial design {8}

**Fig. 1:**
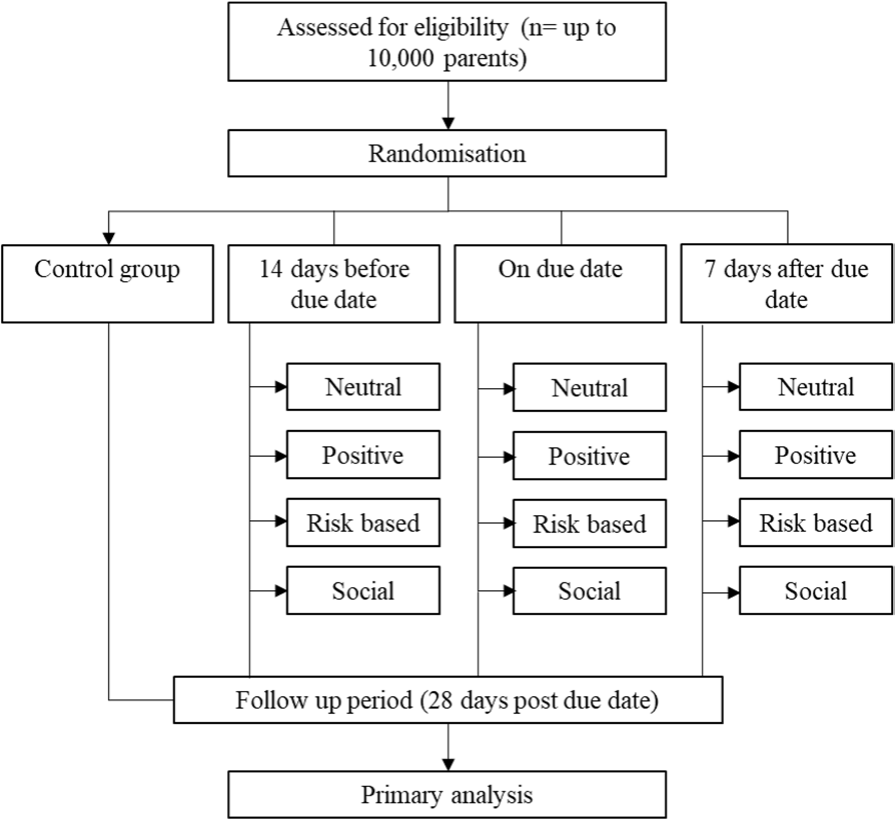
Trial design of the AuTOMATIC study. SMS reminders will comprise different framing options including neutral reminders (indicating vaccine due date), positive (benefit of vaccination), risk-based (risks of disease) and social (responsibility to other children). A full description of the intervention is below in the “Intervention description {11a}” section

This is a pragmatic multi-arm Bayesian adaptive randomised superiority trial that will be enrolling a non-fixed sample size of no fewer than 1500 and no more than 10,000 parents at participating primary care clinics. The design (see Fig. 1) has 13 arms, including 12 active and one usual care arm (no SMS reminder). We will repeatedly measure the time in days from 14 days prior to the vaccine due date to vaccine administration over the childhood vaccine schedule for each child under the care of the enrolled parent, until the administration of each child’s last scheduled vaccine dose at 4 years old, or until the study ends.

The trial will use several adaptive features including adaptive sample size, response adaptive randomisation and arm dropping for inferiority. The allocation of participants to the usual care arm will be fixed at 20% of all randomisations or 0% if discontinued for inferior effectiveness at a subsequent scheduled analysis. Initial randomisation ratios will be equal across the 12 active arms with scheduled analyses using a Bayesian response adaptive algorithm.

The AuTOMATIC trial will perform regular scheduled automated analyses, starting when 1500 parents have reached the primary endpoint, and then after every 500 further parents have reached 28 days after the scheduled date of the index vaccine dose. These analyses will estimate for each arm the posterior probability that it is the best performing arm (highest proportion of index vaccines received by day 28), which will be used to update the future allocation ratios to favour assignment to the better performing arm/s. Enrolment will continue until the maximum sample size is reached or until a pre-specified stopping rule is met. A final analysis will be undertaken at the end of the study to determine secondary objectives.

## Methods: participants, interventions and outcomes

### Study setting {9}

Parents will be enrolled from primary care (general practice, GP) and public vaccination clinics participating in SmartVax across Australia. SmartVax is an Australian software platform installed at GP clinics primarily for active surveillance of adverse events following immunisation (AEFI). The platform interrogates the practice clinical record databases (e.g. Best Practice™ or Medical Director™) and has SMS functionality for issuing surveys to capture self-reported AEFI and vaccine reminders. Clinics across a range of geographical locations will be invited to participate to try to ensure broad sociodemographic representation, including from regional (remote) locations.

### Eligibility criteria {10}

#### Inclusion criteria

To be enrolled, a participant must (1) be a parent of at least one child who is 2 weeks—4 years old and who is registered with a SmartVax-associated clinic or a community vaccination clinic, (2) have their mobile phone number and their child’s name and date of birth recorded in the clinic’s information system and (3) have a child who has received at least one vaccine dose at the clinic previously, except for infants < 2 months old who are yet to receive a first scheduled vaccine dose. This is to target parents who are known to have used the clinic service for childhood vaccination and minimises parents who may be registered at the clinic for medical check-ups but choose to vaccinate their child elsewhere (i.e. community clinics). The system will also check to ensure there is a minimum 4-week window between the last vaccine dose administered and the current vaccine due-date to avoid sending SMS reminders that may confuse parents on catchup schedules.

#### Exclusion criteria

The parent will be excluded if any of the following apply: (1) the parent(s) has requested not to be contacted by the clinic via SMS; (2) the parent(s), in the opinion of clinic staff, would be unsuitable for inclusion in the study, for example because they are known to attend for routine vaccinations elsewhere or because they are conscientious objectors to vaccination; (3) the critical information required to produce the unique study identifier is incorrectly recorded in the clinic’s information system (i.e. parent mobile phone number, child’s date of birth, child’s first and surname); and (4) parents of twin and multiple births will not be eligible as it is not possible to produce a unique child identifier for them in the system.

#### Who will take informed consent {26a}

A waiver of consent has been approved for this study by the University of Western Australia Human Research Ethics Committee (Ref: 2019/RA/4/1/8810) on the basis that the risk to participants is low and that disclosing the study’s objectives is likely to undermine its integrity through selective participation and effects on participant behaviour.

Many patients already receive SMS from clinics, so issuing SMS for vaccine reminders is not considered overly intrusive or burdensome. The study team will have no direct contact with participants and only de-identified data will be extracted and analysed in accordance with the *Australian Privacy Act 1988* [13], the National Statement on the Ethical Conduct of Human Research [14] and other legal and regulatory requirements.

Although participants will not be prospectively informed of their participation in the clinical trial, they can opt-out of receiving further SMS reminders at any stage. Participants who opt out will have their de-identified data included and analysed up to the time of opt-out, but future vaccine occasions will not be captured electronically by SmartVax.

### Interventions

#### Explanation for the choice of comparators {6b}

Usual care (control arm) is no SMS vaccine reminder but might include other non-SMS reminders like telephone or written reminders. Most parents receive a letter from the Australian Government’s universal healthcare payer, Medicare, including a notice if childcare subsidy payments are at risk due to missed vaccinations. Parents in the usual care arm will not receive an SMS reminder for upcoming vaccination, but they may receive an appointment confirmation reminder via SMS if they have already scheduled an appointment where such confirmations are used routinely by the clinic.

#### Intervention description {11a}

SmartVax software interrogates various proprietary electronic clinic information systems to identify children registered with the practice who are age-eligible for a routine childhood vaccine dose. After confirming that the child is either < 2 months old (due for their first vaccine dose) or has received at least one prior vaccine dose at the clinic, the system automates the sending of a personalised SMS reminder to the parent from the clinic. AuTOMATIC will investigate 12 combinations of SMS reminders under a factorial design comprising four message framings and three message timings.

The framing of the SMS reminders will be either (1) framed neutrally, i.e. merely factual with no mention of a benefit or risk; (2) framed positively, i.e. associating vaccination with a direct personal benefit; (3) framed in terms of potential risk, i.e. associating late or missed vaccination with a direct personal health risk; or (4) framed in terms of social importance, i.e. associating vaccination with a broader societal value or benefit.

The timing of the SMS reminders will be either (1) 14 days before the scheduled vaccination due date, (2) on the scheduled due date, or (3) 7 days after the scheduled due date of vaccination if a vaccine has not been received.

If the child is recorded in the clinic information system as vaccinated before the scheduled timing of the SMS reminder delivery, then no message will be sent. The text content of each of the SMS framing and timing options is provided in the *supplementary material*.

#### Criteria for discontinuing or modifying allocated interventions {11b}

A study arm may be discontinued after a scheduled analysis according to the pre-specified rules described below; parents assigned to an active arm which is discontinued will be re-assigned to usual care (no SMS).

#### Relevant concomitant care permitted or prohibited during the trial {11d}

All parents will continue to receive whatever constitutes usual care from their clinic as previously described in {6b}. Any additional pre-emptive SMS reminders for routine childhood vaccination will not be permitted for the duration of the study.

#### Provisions for post-trial care {30}

There is no post-trial care required for participating parents; however, extended use of the trial SMS reminder system will continue for a short period of time after the trial for clinics to adjust to their previous usual care program.

### Participant timeline {13}

#### Eligibility assessment and enrolment (21–28 days prior to due date)

**Fig. 2:**
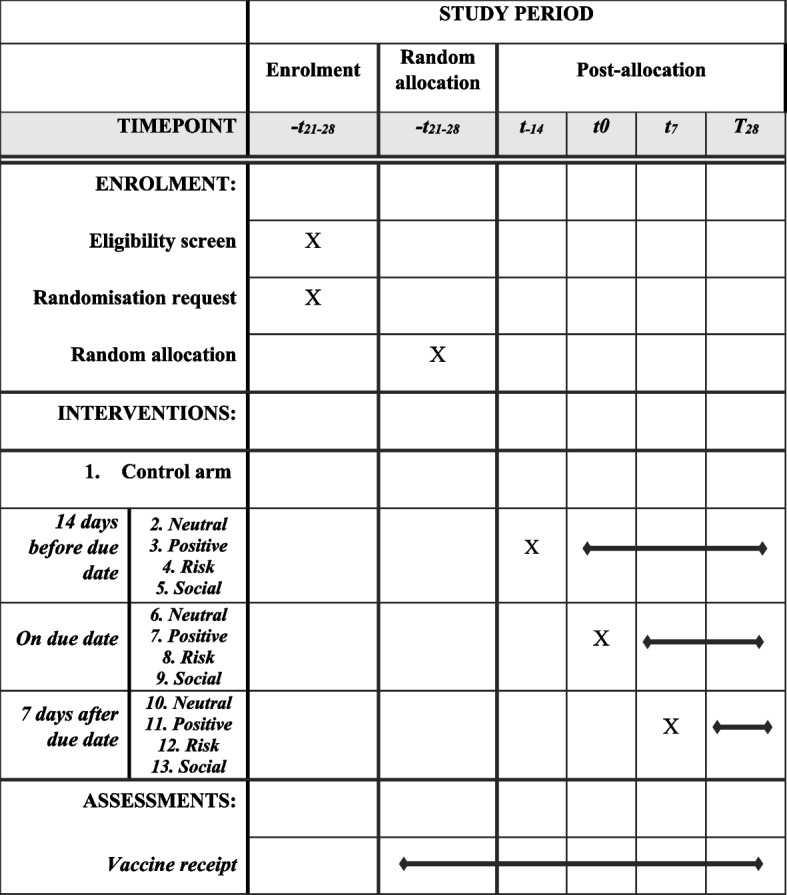
Schedule of enrolment, interventions and assessments for the AuTOMATIC study

The time schedule of events has been designed to allow digital automation (Fig. 2). The SmartVax software will interrogate the clinic electronic practice information system each day to identify any child who is due for a routine scheduled 2, 4, 6, 12, and 18 month or 4 year old vaccine dose in 21–28 days’ time. The software then checks that the child is associated with a parent in the practice information system, that the parent has a valid mobile phone number and that the child is < 2 months old or has had at least one prior vaccine dose received at the clinic.

For each child, the SmartVax software will assign, using a specified algorithm across all participating sites, a unique study identifier based on a scrambling of the child’s date of birth, their first initial and their surname. The parent of the child will also be assigned a unique study identifier based on a scrambling of their mobile phone number. The scrambling algorithm will ensure that parents registered across multiple sites or with multiple children will receive the same randomisation intervention arm as previously assigned and thereby prevent them from being assigned to multiple discordant arms. The scrambling algorithm will generate a deterministic, non-reversible identifier from the input data elements.

#### Randomisation (21–28 days prior to due date)

The SmartVax software will perform an encrypted randomisation request by sending the unique child and parent identifier, the child’s date of birth, the practice identifier and the vaccine scheduled due date to a trial middleware application hosted on a server at the Telethon Kids Institute. The trial middleware application will check if the parent has previously received an arm allocation and if the child and/or parent has been excluded from enrolment.

#### Intervention delivery

Parents will receive an automated SMS reminder at a time and with a message framing in accordance with their assigned arm.

#### Recording of vaccine receipt (vaccine due date followed up to 28 days later)

Each day, the SmartVax system will identify which children have received a vaccine as recorded in the clinic software system; this data will be uploaded to the trial middleware system and subsequently to the study database.

#### Sample size {14}

At least 1500 parents (when the first interim analysis is scheduled to occur) and no more than 10,000 parents will be enrolled unless one or more of the pre-specified stopping rule(s) is/are met at a scheduled analysis.

The maximum sample size was selected on the basis of trial feasibility and, due to the adaptive nature of the trial, the usefulness of sample size was evaluated via simulation which was used to assess the trials operating characteristics.

Under the planned decision thresholds (the “Interim analyses {21b}” section), we estimated a probability of approximately 0.06 of incorrectly declaring any active arm *superior* when all were equivalent to usual care and a probability of less than 0.02 of incorrectly declaring a specific active arm *effective* when equivalent to usual care. We estimated a probability of approximately 0.87 for declaring an active arm effective when it improved the odds of 28-day vaccination by a factor of 1.4 over all other interventions.

Further details of the trial simulations are provided in the *supplementary material*.

#### Recruitment {15}

We will maximise involvement of eligible clinics for this study through an established network that is already associated with SmartVax under its existing adverse event monitoring program and Primary Health Networks (coordination centres for healthcare services within community regions). At the time of protocol publication, SmartVax adverse event monitoring is used by over 600 clinics across Australia.

### Assignment of interventions: allocation

#### Sequence generation {16a}, concealment mechanism {16b} and implementation {16c}

The parent is the unit of randomisation and their allocation will be automatically generated by the middleware application on a daily basis. A randomisation sequence is digitally pre-generated and stored within the middleware application; the next assignment is taken from this ordered list as each new eligible parent is enrolled. The randomisation sequence is generated using a mass-weighted urn design [15] to allow for incorporation of the non-integer target allocation weights generated from the response adaptive randomisation procedure.

At trial initiation, the allocation probability is 1/5 for usual care and 1/15 for each of the 12 active intervention arms. The allocation of 1/5 to the usual care arm will remain fixed throughout the trial unless dropped according to a pre-specified decision rule.

After each scheduled analysis, the probability of allocation to each active intervention arm will be updated such that it is proportional to the scaled posterior probability that the arm is best among all active arms with respect to the primary outcome. In this manner, better performing active arms will receive progressively higher weighting of allocations than worse performing active arms. If an active arm is performing significantly worse than the usual care arm, then that arm will be discontinued (the probability of allocation to the arm will be set to zero), and those parents will be manually switched to usual care, and subsequent outcome data will be excluded from analyses.

### Assignment of interventions: blinding

#### Who will be blinded {17a} and procedure for unblinding if needed {17b}

Parents in the active intervention arms will be aware of any SMS received, but they will not be informed of the study or the arm they have been allocated. Clinic staff may be aware of the study and its objectives but will not be notified of the arm assigned to each parent, although no steps will be taken to prevent them for ascertaining this directly from the parent. Study investigators, except for the trial statistician, will be blinded to arm assignments at both the individual and aggregate level. No procedures are required for breaking codes.

### Data collection and management

#### Plans for assessment and collection of outcomes {18a}

Whenever a child attends a participating clinic for vaccination, the vaccine type and vaccination date and time will be entered in the electronic practice information system and will be transferred automatically to the study REDCap database. Data on participants will continue to be captured until the trial is complete, the child receives their 4-year-old scheduled vaccine doses or the parent opts out of receiving further SMS reminders.

The data captured will include the unique practice identifier; unique parent study identifier; unique child study identifier, parent postcode, child date of birth, current scheduled vaccine (age in months) and due date, assigned study arm, scheduled date of issuing the SMS reminder, SMS sent date, SMS delivery success, most recent prior vaccination date at the clinic, type of vaccine administered and opt out of future SMS.

#### Data management {19}

No data will be specifically entered for this trial, either by the investigators or by clinic staff, nor will there be a study case report form. Instead, all data necessary for the analysis will be extracted directly from the source (the clinic’s practice information system) using SmartVax software. As this trial is largely automated, regular data monitoring will be performed by the trial coordinator and trial statistician to ensure integrity of data flow and that analyses are occurring as specified in the protocol.

#### Confidentiality {27}

Identifiable data will only be stored on each clinic’s practice information system according to their usual procedures. De-identified data required for study implementation and analysis will be exported daily using TLS (transport layer security) encrypted HTTPS (hypertext transfer protocol secure) transfer to a password-protected database, located on a secure server at Telethon Kids Institute. Individuals will only be identified by a unique study identifier to allow cross-site linkage while maintaining confidentiality.

### Statistical methods

#### Statistical methods for primary and secondary outcomes {20a}

The full statistical analysis plan for this study is provided in the *supplementary material*. Statistical analyses of the primary outcome will use a Bayesian logistic regression model. The model will be used to infer the log-odds of vaccination by 28 days after the scheduled due date for the index vaccine, for each active intervention arm relative to usual care. The model will account for variation in outcomes by including terms for: the intervention (each message framing by timing combination), the vaccine dose schedule point (age), the clinic and the scheduled vaccine due date in calendar time (aggregated into 4 week blocks relative to the most recent due date).

Using this model, we will report posterior summaries for the parameters of interest and evaluate for each active arm the probability that it is superior to the usual care arm and the probability that it maximises the log-odds of vaccination among all the other considered intervention arms.

The primary outcome model priors were selected as Normal(0, 2.5^2^) to be mildly regularising on the intervention effects on the log-odds scale while reflecting neutrality with respect to their effect on the primary outcome. The intercept term was specified as Normal(1.37, 2.5^2^) to be weakly informative based on an expected 28-day vaccination rate of 80%. Priors on other model coefficients were specified as Normal(0, 2.5^2^) to be weakly informative. Prior sensitivity analyses will be conducted as part of the final trial analyses to assess the influence of the pre-specified priors. Sensitivity of the decision error rates to the assumed priors were assessed as part of the pre-trial simulations and the prior influence was deemed negligible given the planned sample size.

#### Interim analyses {21b}

Regular analyses will be conducted throughout the trial. The first analysis will be scheduled to occur after 1500 index children have been followed to 28 days after the scheduled date for their index vaccine. After that first analysis, future analyses will be scheduled to occur after every additional 500 index children have been followed to 28 days after the scheduled due date for their index vaccine. These analyses will include all data on all index vaccination occasions which are at least 28 days past their scheduled due date.

At each analysis, the model parameter’s posterior distribution will be used to assess trial decision rules and to update the target allocation probabilities for each active arm. At each analysis, we will calculate the posterior probability that (i) each active arm is *effective* compared to usual care, that is, it increases the log-odds of vaccination relative to usual care, (ii) each active arm is *superior* to all other active arms, that is, it increases the log-odds of vaccination more than all other interventions, and (iii) an active arm is, on average (equally-weighted across all interventions), effective compared to usual care.

Based on these calculated probabilities, a decision will be made after each analysis whether to (i) discontinue an active arm for *lack of effectiveness* if there is a small (low) probability that it increases the proportion vaccinated by 28 days compared to usual care only; (ii) discontinue the usual care arm if, compared to usual care, all active arms collectively on average, or any particular active arm, has/have high probability of increasing the proportion vaccinated by 28 days; (iii) discontinue the trial for *futility* if all active arms have been discontinued for lack of effectiveness; and (iv) discontinue the trial for *success* if any active arm has high posterior probability of being the best overall, and usual care has been discontinued.

The probability thresholds for discontinuing are scaled according to the current sample size relative to the maximum sample size and have been chosen based on extensive simulations to achieve good trial-wise frequentist operating characteristics. Denoting by *n*_*t*_ the sample size at the *t*th scheduled analysis, for effectiveness, the threshold is 1 – 0.01(*n*_*t*_*/10,000)*^*0.5*^; for ineffectiveness, the threshold is 0.01(*n*_*t*_*/10,000)*^*0.5*^; and for superiority, the threshold is 0.95–0.25 (*n*_*t*_*/10,000)*^*0.5*^. Following the analysis, the target allocation probabilities to each continuing active arm will be updated to be proportional to the variance and sample-size scaled probability that each arm is superior.

If after 10,000 parents have been randomised and none of the above trial stopping rules have been met, no further parents will be randomised, and posterior summaries of model parameters and comparisons of interest will be calculated.

#### Methods for additional analyses (e.g. subgroup analyses) {20b}

The effect of SMS vaccine reminders on the primary endpoint will be determined in the final analysis for each vaccine dose schedule point for childhood vaccination (i.e. at 2, 4, 6, 12, and 18 months and 4 years).

#### Methods in analysis to handle protocol non-adherence and any statistical methods to handle missing data {20c}

Where digital errors may occur, for example, a clinic server malfunction leading to no SMS being sent as assigned, participants will be included in each scheduled analyses according to their intended assignment. As this is equally likely to occur in any of the active arms, there is no significant risk of bias. The data capture system is designed to ensure all fields are populated so missing data is not expected; however, in the event any data is missing, we will not use imputation in our analyses.

#### Plans to give access to the full protocol, participant level-data and statistical code {31c}

The full protocol can be requested at any time by contacting the corresponding author. Population-level data can be requested following publication of the study results and will be released subject to necessary human research ethics committee approvals. Statistical code will be shared in certain approved cases, subject to the author/s properly attributing any derived code used for future work.

### Oversight and monitoring

#### Composition of the coordinating centre and trial steering committee {5d}

Coordination of the trial is by the Telethon Kids Institute and comprises the coordinating principal investigator (CPI), study coordinator, trial statistician (unblinded) and software developers. Important trial updates will be promptly communicated to study investigators and other personnel.

#### Composition of the data monitoring committee, its role and reporting structure {21a}

An independent statistical monitoring committee comprising two independent statisticians will be appointed to provide oversight and external validation concerning adherence to the protocol and analysis plan. This includes appropriate adaptation of the randomisation allocation probabilities and implementation of the trial stopping rules. They will meet at least twice a year or within 28 days of any trigger for discontinuation, to monitor and review accumulating efficacy analyses and to make recommendations to the CPI on any reasons why the trial should be modified or should not continue. The committee may request to be unmasked to the intervention allocation of individual participants and/or to have additional blinded or unblinded analyses performed by the trial statistician.

#### Adverse event reporting and harms {22}

There will be no adverse event monitoring; however, the trial statistician and trial coordinator will review the number of SMS opt-out requests over the course of the study. If an active arm is identified as having a high number of opt-out requests, the independent statistical monitoring committee will be notified to discuss the appropriate action, including possible elimination of a particular intervention arm if required.

#### Frequency and plans for auditing trial conduct {23}

Direct access will be granted to authorised representatives from the sponsor or the regulatory authorities to permit trial-related monitoring, audits and inspections.

#### Plans for communicating important protocol amendments to relevant parties (e.g. trial participants, ethical committees) {25}

Protocol amendments will be submitted directly to the ethics committee for review. Participants will not be informed of any amendments.

#### Dissemination plans {31a}

The results of this study will be made openly available and will be submitted for peer review.

#### Patient and public involvement

A community reference group was involved in the project design. The group was supportive of the request for a waiver of consent and contributed to informing the acceptability of message framing options of the SMS reminders. Messages considered to be broadly acceptable were then presented to wider group of 80 parents of young children across Australia via an electronic and paper-based survey; the parents ranked each message from most to least preferred. One SMS message in each framing category was then selected for use in the study based on these preferences.

## Discussion

Despite good coverage for routine childhood vaccines in Australia, under-vaccination remains a challenge, and strategies to improve vaccine uptake and timeliness are still required to prevent transmission of preventable infectious diseases. Here, we propose a unique resource-efficient adaptive study that contains almost complete digital automation of trial processes, including eligibility screening, randomisation, delivery of the intervention, data capture and regular analyses with trial adaptations. We believe this may be the most digitally automated trial ever undertaken globally and, we anticipate, will become an exemplar for implementing embedded adaptive trials in primary care.

Given the risks and consequences of under-vaccination and delayed vaccination, it is important to identify effective ways of increasing vaccine uptake at both a local and national level [16]. While much effort has been invested in strategies to motivate vaccination among hesitant parents, for many parents, simply reminding them when vaccines are due using SMS reminders may be a cheap, effective and implementable strategy at a large scale. The effectiveness of an SMS reminder plausibly depends on different factors, such as who the message is from, the framing of the message, and the timing of message relative to the vaccine due date.

Previous studies have found high acceptance among parents for vaccine reminders via SMS [17–23], with one reporting that parents preferred receiving SMS reminders over telephone calls or letters [22]. Although these data support the use of SMS reminders, they have largely come from studies in specific sub-populations (for example urban, low-income parents) and from the USA where recipients typically pay to receive messages; therefore, these studies may not be generalisable to a whole-of-population intervention outside the USA.

Most parents report that their GP is their most important source for information about vaccination [2]. Data also show that parents trust information from healthcare providers such as GPs, with a personal recommendation from a GP being a strong driver of the decision to vaccinate [24]. Accordingly, we hypothesise that an SMS sent by a child’s GP (or usual vaccine provider) might be an effective way to drive vaccine uptake and may be more effective than a message sent from another source, such as a government health department. This study will therefore evaluate messages issued by those vaccine providers.

To our knowledge, no studies to date have yet examined the influence of different message framing strategies in the context of SMS reminders on routine childhood vaccine uptake. In some contexts, a risk-framed message which appeals to potential harm arising from failure to act (like risk of contracting a preventable infection) can be effective [25], but in other contexts, these messages may have either little effect or may paradoxically associate the harm with the desired behaviour itself [26].

We are also not aware of previous studies examining the influence of multiple timing options on the effectiveness of SMS reminders for childhood vaccination. Sending an SMS vaccine reminder to all parents before the scheduled date might afford them sufficient time to organise vaccination, but reminders that are issued too early may be less effective for motivating action. Targeting parents of children who have already passed the scheduled due date may be most effective for motivating action, and if shown to be as or more effective than earlier reminders, may greatly reduce costs while also minimising burdensome reminders on parents who do not require them.

In summary, this adaptive trial will examine the effectiveness of SMS reminders on vaccine uptake and timeliness for routine childhood vaccination. Evaluating different health messaging strategies using simple SMS may serve as one effective aspect within a multimodal program for influencing vaccine acceptance and behaviour.

### Trial status

At the time of this submission, 5462 parents have been randomised in the trial, with 5370 index cases. The first scheduled analysis was performed in August 2021. The anticipated date for completion of enrolment is December 2023.

## Supplementary Information

**Additional file 1.**

## Data Availability

Individual-level de-identified data can be provided to external parties with relevant ethics and governance approvals.

## Abbreviations

AEFI: Adverse event following immunisation
CPI: Coordinating principal investigator
GP: General practitioner
HTTPS: Hypertext transfer protocol secure
RCT: Randomised control trial
SMS: Short message service
TLS: Transport layer security
US: United States of America
