# Supplementary material for: The AuTOMATIC trial: a study protocol for a multi-arm Bayesian adaptive randomised controlled trial of text messaging to improve childhood immunisation coverage
Source: Trials. 2023 Feb 7;24:97. doi: 10.1186/s13063-023-07097-3 (PMC9904255; doi:10.1186/s13063-023-07097-3)
Supplement: Supplementary file 1 — Additional file 1. [file 13063_2023_7097_MOESM1_ESM.pdf]

Supplementary Material:

A: SMS Examples

Please note that XX/XX/XX will be populated by a date and call XXXX XXXX will be populated by the clinic phone number.

Content	Example text: 14 days before scheduled vaccine date	Example text: scheduled vaccine date	Example text: 7 days after scheduled vaccine date
**Neutral**	"[CLINIC NAME]: [Child's name] is due for a X-month vaccination on XX/XX/XX. To book or opt-out of SMS, call XXXX XXXX. Replies are not monitored, pls ignore SMS if you have an appt"	"[CLINIC NAME]: [Child's name] is due for a X-month vaccination today. To book or opt-out of SMS, call XXXX XXXX. Replies are not monitored, pls ignore SMS if you have an appt"	"[CLINIC NAME]: [Child's name] is overdue for a X-month vaccination. To book or opt-out of SMS, call XXXX XXXX. Replies are not monitored, pls ignore SMS if you have an appt"
**Positive**	"[CLINIC NAME]: [Child's name] is due for a vaccination on XX/XX/XX. Vaccinating on time gives [Child's name] the best defence against infectious diseases. To book or opt-out of SMS, call XXXX XXXX. Replies are not monitored, pls ignore SMS if you have an appt"	"[CLINIC NAME]: [Child's name] is due for a vaccination today. Vaccinating on time gives [Child's name] the best defence against infectious diseases. To book or opt-out of SMS, call XXXX XXXX. Replies are not monitored, pls ignore SMS if you have an appt"	"[CLINIC NAME]: [Child's name] is overdue for a vaccination. Vaccinating on time gives [Child's name] the best defence against infectious diseases. To book or opt-out of SMS, call XXXX XXXX. Replies are not monitored, pls ignore SMS if you have an appt"
**Negative**	"[CLINIC NAME]: [Child's name] is due for a vaccination on XX/XX/XX. Delaying a child's vaccination can put them and other children at risk. To book or opt-out of SMS, call XXXX XXXX. Replies are not monitored, pls ignore SMS if you have an appt"	"[CLINIC NAME]: [Child's name] is due for a vaccination today. Delaying a child's vaccination can put them and other children at risk. To book or opt-out of SMS, call XXXX XXXX. Replies are not monitored, pls ignore SMS if you have an appt"	"[CLINIC NAME]: [Child's name] is overdue for a vaccination. Delaying a child's vaccination can put them and other children at risk. To book or opt-out of SMS, call XXXX XXXX. Replies are not monitored, pls ignore SMS if you have an appt"
**Social benefit**	"[CLINIC NAME]: [Child's name] is due for a vaccination on XX/XX/XX. Vaccinating on time protects [Child's name] and other children who are too young to be vaccinated. To book or opt-out of SMS, call XXXX XXXX. Replies are not monitored, pls ignore SMS if you have an appt"	"[CLINIC NAME]: [Child's name] is due for a vaccination today. Vaccinating on time protects [Child's name] and other children who are too young to be vaccinated. To book or opt-out of SMS, call XXXX XXXX. Replies are not monitored, pls ignore SMS if you have an appt"	"[CLINIC NAME]: [Child's name] is overdue for a vaccination. Vaccinating on time protects [Child's name] and other children who are too young to be vaccinated. To book or opt-out of SMS, call XXXX XXXX. Replies are not monitored, pls ignore SMS if you have an appt"

Supplementary Material:
B: AuTOMATIC Statistical Analysis Plan

AuTOMATIC

Statistical Analysis Plan

Adaptive Trial of MessAging to improve Immunisation Coverage

Date

November 2020

Versions

SAP version: 1.0

Protocol version: 2.0, October 2020

Study Identifiers

ANZCTR number: U1111-1189-6054

HREC reference: RA/4/1/8810

Contents

Version History 2
List of Authors 2
Approvals 3

1 Definitions 4

2 Introduction 5

3 Study Details 5
3.1 Background and Rationale 5
3.2 Intervention 5
3.3 Study Objectives 6

4 Study Design 8
4.1 Type 8
4.2 Estimands 9

4.3 Randomisation 10
4.4 Sample size 11

5 Trial Population **11**
5.1 Eligibility criteria 11
5.2 Analysis datasets 12

6 Analysis Methods **13**
6.1 Data 13
6.2 Analysis of primary outcome 13
6.3 Sensitivity analyses of primary outcome 16
6.4 Statistical quantities 16
6.5 Analysis of secondary outcomes 18
6.6 Interim analyses and trial adaptations 20
6.7 Subgroup analyses 22
6.8 Missing data 22
6.9 Software 22

7 Operating Characteristics **23**
7.1 Simulation scenarios 23
7.2 Simulation results 24

References **64**

Version History

Version	Date	Summary
1.0	November 2020	Statistical analysis plan AuTOMATIC

List of Authors

Mr James Totterdell, Senior Biostatistician, University of Sydney

Dr Julie Marsh, Senior Biostatistician, Telethon Kids Institute

Approvals

The undersigned have reviewed this plan and approve it as final. They find it to be consistent with the requirements of the protocol as it applies to their respective areas. They also find it to be compliant with ICH-E9 principles and, in particular, confirm that this analysis plan was developed in a completely blinded manner (i.e. without knowledge of the effect of the intervention(s) being assessed).

Trial Statistician	Date
Principal Investigator	Date

1 Definitions

Term	Definition
Estimand	Estimands align protocol objectives with the quantification of the intervention effect(s). It is defined as "the target of estimation to address the scientific question of interest posed by the trial objective" in ICH E9(R1).<br><br>An estimand is a combination of eligibility criteria (population of interest), endpoint definition, treatment description (including the comparator), statistical analysis, treatment of intercurrent events post-randomisation (eg. missing values, non-compliance, use of rescue medication or interventions, etc.) and a population level summary (e.g. pairwise differences in the log hazard rates between the interventions).
Index child	The first child scheduled for vaccination for a given parent after the parent has been randomised.
Index vaccine	The first scheduled vaccine date for the index child after the parent's randomisation date.
Late vaccination	A child who has not received the recommended vaccinations within 4 weeks (28 days) of the scheduled date according to the standard childhood immunisation schedule.
Parent	A parent of a child including a person who is regarded as the legally responsible caregiver of the child under law.
Sites	Participating SmartVax GPs and community-based providers.
Subsequent child	Any child belonging to a parent whom, at the moment of enrolment, already has another child enrolled.
Under-vaccination	A child who has not received all of the age-appropriate vaccinations recommended by the standard childhood immunisation schedule.

2 Introduction

The purpose of this document is to describe the statistical methods which will be used to analyse data in the AuTOMATIC: Adaptive Trial of MessAging to improve Immunisation Coverage trial. It is written for statisticians and should be read in conjunction with the protocol.

The basic trial structure, objectives, endpoints, and quantities of interest are outlined in Sections 3 and 4, the eligibility criteria are explained in 5, the statistical model and quantities of interest are introduced in Section 6, trial adaptations are defined in Section 6.6, and a summary of trial operating characteristics or provided in Section 7.

This statistical analysis plan (SAP) is based on version 2.0 of the study protocol, October 2020.

3 Study Details

3.1 Background and Rationale

The potential of SMS and the effect of message framing and timing on vaccination timeliness has not been studied in an Australian setting. We hypothesise that SMS reminders emanating from a family's usual vaccine provider may be more effective for improving vaccination uptake and timeliness than, for example, impersonal messages originating from a government source.

The study aims to determine the optimal timing of sending reminders, as sending an SMS reminder to everyone before the scheduled-date may have a similar effectiveness to targeting parents of children who are over-due. However, from a practical perspective, reducing the costs associated with the sending of SMS reminders will have an impact on how they are implemented in routine clinical care. In addition, the optimal framing of the messages will be examined, as a neutral SMS reminder may be all that is necessary to convince parents to vaccinate on time. All messages were assessed by a panel of consumer representatives prior to obtaining ethics approval.

3.2 Intervention

SmartVax is a vaccine safety initiative originally developed to monitor adverse reactions following vaccination. The system integrates with all major GP patient information systems and sends an automated SMS to parents 3 days after receiving a vaccine to ask whether their child had any reactions (e.g. fever, rash). Parents can respond to the SMS and are prompted to complete an electronic survey asking for details of the side-effects if they indicated yes ("Y").

Recently, SmartVax has been further developed to send optional automated SMS 'pre-call' reminders to notify parents that their child's next vaccination is nearly due, due or over-due. This study will investigate the effectiveness of sending SMS reminders using this technology. The SmartVax platform will interrogate

the medical records of children registered at a practice to determine when vaccinations are due, and to send an automated SMS reminder prompting parents to call the clinic and schedule an appointment at the appropriate time. Following vaccine administration, the SmartVax platform will be able to use the details of the vaccination (entered electronically by site staff) and the child's date of birth to schedule the next SMS reminder. The platform will be used to send SMS reminders at different time points and with different message framing. We will examine the effect of these interventions on vaccine uptake. The intervention is designed to influence the parent, therefore, each parent will be allocated at random to a single intervention arm, which will apply to any children under their care at all scheduled vaccine occasions.

Twelve intervention arms, consisting of the combination of four different message framings and three different timings will be investigated (i.e. a 4x3 factorial design). The text message framings are classified as either:

1. positive in tone (espousing a personal benefit from vaccination),
2. negative in tone (espousing a risk from late or failed vaccination),
3. neutral in tone (factual and conveying neither benefit nor risk), or
4. social norm (conveying the societal preponderance of vaccination).

The messages may be sent at timings:

1. 14 days before the scheduled due date,
2. on the scheduled due date,
3. 7 days after the schedule due date.

Additionally, a control arm that receives no SmartVax SMS reminder will be included as a reference arm. This control arm will still receive standard care from the practice. Actual standard care may vary slightly from practice to practice and may include, for example, a letter from Medicare to signal an overdue vaccination, a notice from Centrelink if the parent is receiving a childcare rebate or a telephone/written reminder from the GP clinic. Parents in the standard care group will not receive an unsolicited SMS reminder for an upcoming vaccination, but participants may receive an appointment reminder or confirmation via SMS if they have scheduled an appointment if that is usual practice for their provider.

3.3 Study Objectives

The aim of the study is to determine whether provider-initiated SMS reminders are effective for improving the timeliness of routine vaccination among Australian children. The parent (whose behaviour we seek to change) can only be considered naive to the intervention on the first child-vaccination occasion, therefore, the primary analysis will be performed on the outcome of the first intervention occasion for each parent.

The specific objectives and the related outcomes are tabled below.

3.3.1 Primary Objectives

Objective	Outcome
To determine the effect of different timing and framing of a personalised SMS reminder, emanating from a family's vaccine provider (general practice or community vaccination clinic), on the proportion of children vaccinated within 28 days of the scheduled due date for routine childhood vaccination compared to usual practice. The objective of most interest is identification of the best message framing and timing combination out of those considered in terms of proportion of children vaccinated within 28 days of the scheduled due date.	Vaccination status (vaccinated or unvaccinated) at 28 days after scheduled vaccine due date for the index child for the index vaccine. On occasion, vaccination might occur prior to the due date. Such occasions will be counted as vaccinated by 28 days after the due date if it occurs no more than 14 days before the scheduled due date.
	Parents may have multiple children eligible for study inclusion and each child may receive multiple vaccine doses during the trial period. The index child is the first child scheduled to be vaccinated after the parent's randomisation date and the index vaccine is the first scheduled vaccine date for each child after the parent's randomisation date

3.3.2 Secondary Objectives

Objective	Outcome
To determine the effectiveness of vaccine provider initiated SMS reminders of varying content and timing for reducing the time to vaccination, relative to vaccine due date, for routine childhood vaccines	Time to vaccination (measured in days) from 14 (timing of earliest intervention) days before the scheduled due date up to 42 days after the scheduled due date. Calculated as the difference between date of vaccine administration and due date as recorded in the SmartVax system for the *index vaccination* of each parent randomised. Events occuring more than 42 days after the scheduled due date are considred right censored
	Time to vaccination (measured in days) from 14 days before the scheduled due date (timing of earliest intervention) up to 42 days after the scheduled due date. Calculated as the difference between date of vaccine administration and due date as recorded in the SmartVax system for *all scheduled vaccinations* of each parent randomised. Events occuring more than 42 days after the scheduled due date are right censored
To further evaluate the effectiveness of vaccine provider initiated SMS reminders of varying content and timing for improving the rate of timely vaccination for routine childhood vaccines	Vaccination status (vaccinated or not) by 28 days after the scheduled due date measured by the difference between date of vaccine administration and due date as recorded in the SmartVax system for *all scheduled vaccinations* of each parent randomised

4 Study Design

4.1 Type

This is a Bayesian adaptive, factorial, superiority trial. Frequent interim analyses will be performed to assess if a combination of message framing and timing is superior to the other interventions. Intervention performance with respect to the primary outcome will be used to inform response adaptive randomisation leading to a higher allocation of future participants to better performing interventions. Interventions which perform worse than control will be dropped from the trial. If any intervention is better than control, or on average receiving an intervention is better than receiving control, the control group will be dropped.

In addition to the response adaptive randomisation, pre-defined decision rules for early stopping are:

1) Stop for superiority if there is substantial evidence that one intervention arm results in a higher 28 day vaccination proportion than all others including control.

2) Stop for harm if there is substantial evidence that all intervention arms have a lower 28 day vaccination proportion than control or there is strong evidence that on average an intervention is worse than control.

4.2 Estimands

Estimands provide a structured framework to increase the transparency and precision in describing an intervention effect of interest. They inform decision making within a clinical trial setting by clearly describing the risks and benefits of an intervention. Interventions designed to increase childhood vaccine uptake and coverage are assessed in terms of timely percentage uptake and time-to-event endpoints, respectively. However, these endpoints address different objectives and the effect of each intervention may differ over time or by the age of the child at scheduled vaccination. It is anticipated that the results may differ between the estimands as each addresses a different hypothesis. Estimands and sensitivity analyses are described according to recommendations in ICH E9-R1.

4.2.1 Primary Estimand

Objective: To determine the real-world comparative effectiveness of the first vaccine-provider initiated SMS reminder framing and timing for increasing the proportion of children vaccinated within 28 days of scheduled routine vaccine date.

Strategy: Treatment-policy, irrespective of age-at-scheduled-vaccine.

Population of interest: Parents of index children receiving their index vaccine, in addition to meeting the study eligibility criteria in Section 5.1. No details are provided from participating sites concerning any reasons for delayed child vaccine administration or vaccines administered outside the site.

Endpoint: Evidence of administration of vaccine for index vaccine at participating site within 28 days of scheduled vaccine date (binary endpoint).

Treatment description: Control arm and 12 intervention arms as detailed in Section 3.2. Comparisons will be made between all arms.

Treatment of intercurrent events: Parents will be included in the intervention group they were allocated to, irrespective of whether they received the SMS text or materials related to the site standard-of-care or not. In the absence of evidence of vaccine administration within 28 days of scheduled date at the participating site, including due to participants who are lost to follow up or move out of the GP-site catchment area, it will be assumed that the vaccine has not been administered.

Statistical method: Bayesian logistic regression; details in Section 6.2.

Population summary: The posterior probability that an intervention is superior to all other invention arms. In addition, posterior summaries from the statistical model parameters for average SMS text framing and

timing, relative to the overall intervention effect and interaction effects for framing and timing combinations, will be presented as point estimates and highest density intervals (HDI). Further details are given in Section 6.2.

Sensitivity analysis: The sensitivity of the results will be investigated by varying the model covariates. For example, the interaction terms will be excluded, and main effects terms excluded for comparison of results. Further details are given in Section 6.3.

4.2.2 Secondary Estimand

Objective: To determine the real-world timeliness of first vaccine-provider initiated SMS reminder framing and timing for reducing the time to vaccination for routine childhood vaccines.

Strategy: Treatment-policy, irrespective of age-at-scheduled-vaccine.

Population of interest: Parents of index children receiving their index vaccine, in addition to meeting the study eligibility criteria in Section 5.1. No details are provided from participating sites concerning any reasons for delayed child vaccine administration or vaccines administered outside the site.

Endpoint: Time to vaccination at participating sites from the date of randomisation. Vaccine administration which occurs later than 42 days after the vaccine due date will be censored. The reminder system records no information as to which of the scheduled vaccines have been given. Therefore, vaccinations which occur within 14 days of a subsequently scheduled vaccination are assumed to correspond to the upcoming scheduled dose rather than the preceding one.

Treatment description: Control arm and 12 intervention arms as detailed in Section 3.2.

Treatment of intercurrent events: Parents will be included in the intervention group they were allocated to, irrespective of whether they received the SMS text or materials related to the site standard-of-care or not. In the absence of evidence of vaccine administration within 42 days of scheduled date at the participating sites, including due to participants who are lost to follow up or move out of the GP-site catchment area, it will be assumed that the vaccine has not been administered and the endpoint will be right censored at 42 days after the due date.

Statistical method: Bayesian proportional continuation ratio regression model; more details in Section 6.5.

Population summary: The posterior summary of conditional odds ratio and restricted mean survival time under each intervention relative to the control group.

4.3 Randomisation

The parent is the unit of randomisation, as it is their behaviour we seek to change. Parents of eligible children will therefore be allocated at random to one of the 13 arms. Each child may receive more than

one scheduled vaccination during the study period, but the parent will receive the same intervention for all eligible children under their care for all scheduled vaccinations.

To avoid randomising the same parent to different arms across different sites (in case they have registered at multiple SmartVax GPs and/or community-based providers) the random allocation will be automatically generated from a central de-identified allocation list. The use of centralised randomisation will ensure that if the parent is registered at multiple participating SmartVax sites, they will be allocated to the same intervention arm for all eligible children under their care, at all scheduled vaccine doses and across all participating sites throughout the study period. The standard de-identification algorithm employed across all sites will ensure that the same unique parent ID will be generated at all sites based on the recorded mobile phone number. It is anticipated that there will be a low rate of incorrect or out of date mobile phone numbers recorded in the site records and that this will be equally likely to occur across all intervention arms.

Prior to the first interim analysis, parents will be equally likely to be randomised to any of the interventions and a higher ratio will be allocated to the control arm; this is the run-in period before response-adaptive randomisation commences. Following each interim analysis, the allocation ratios to each intervention arm will be updated and a new allocation list generated on the middleware application. Any arms which have been dropped will receive zero allocations and the control arm allocation ratio will be fixed unless dropped. All parents randomised after an interim will then be allocated to arms according to the new allocation list.

4.4 Sample size

The minimum sample size will be 1,500 index vaccinations due to the timing of the first interim analysis. A maximum of 10,000 parents will be randomised unless a pre-specified stopping rule is met at interim analyses. This sample size was chosen based on simulations of the adaptive trial (see Section 7).

5 Trial Population

Up to 10,000 parents will be enrolled from GP and public vaccination clinics participating in SmartVax across Australia. A diverse geographical range of clinics will be invited to participate to ensure representation from a broad socio-demographic cross section of suburbs, including from regional Australia. Agreements will be sought from all clinics, and governing bodies prior to enrolling at these sites. Study participation (parent and child/children) is from parent's randomisation until the last scheduled vaccine dose has been administered before five years of age for any child under their care or until the trial concludes.

5.1 Eligibility criteria

To be included in the study ALL of the following criteria must be satisfied;

- Parents of children aged 6 weeks – 4 years (strictly less than 5 years) who are registered with a SmartVax registered GP clinic or community vaccination clinic or whose clinic has expressed interest in SmartVax.
- Parents must have a mobile phone number registered with the vaccine provider.
- Eligible children must have their details entered into their electronic health record, including the parent mobile phone number and the child's date of birth and name.

The participant will be excluded if ANY of the following apply:

- The parent(s) of the child have previously requested not to be contacted by the clinic via SMS.
- Parents who in the opinion of clinic staff would be unsuitable for inclusion in the study, for example because they are known to attend for routine vaccinations elsewhere, they have relocated outside of the clinic catchment area, the registered mobile phone number is known to be obsolete or wrong, or because they are registered as conscientious objectors to vaccination.
- The critical information required to produce a unique identification number has not been entered properly into the practice's electronic medical record (i.e. parent mobile phone number, child's date of birth, child's first and surname).
- Children known or suspected to be twins and triplets will be excluded; producing a unique identification number will not be possible for siblings with the same birthdate.

It may be necessary to withdraw the following participants from the analysis if:

- Any of the exclusion criteria are met subsequent to enrolment and before the end of follow-up.

For some participants, the 2 month scheduled vaccination may be administered at 5 weeks. Participants whose 2 month scheduled vaccination was given more than 14 days before the due date will be excluded, and their next scheduled vaccination will be selected as the new index vaccination.

5.2 Analysis datasets

The primary analysis dataset consists of index vaccines for each parent. The secondary outcome datasets will consist of all children and all vaccines for each parent. At the time of an interim analysis, all index vaccinations which are past due (greater than 28 days after the scheduled due date) relative to the analysis reference date will be included in the analysis set. The reference date will be one week prior to the date on which the analysis is being run to allow the system to catch-up on any outstanding updates.

The data will be analysed and reported on an intention-to-treat (ITT) basis with all randomised participants contributing according to the estimands defined in 4.2. In particular, we cannot know that the SMS reminders were received or read by the participants, only that they were delivered. Participants will be analysed in the arm they were allocated to.

6 Analysis Methods

6.1 Data

Individual-level de-identified and encrypted data for every enrolled parent with associated child vaccination outcomes will be exported electronically and in an encrypted fashion to the secure REDCap database on the Telethon Kids Institute server. This data will include:

1. the child's date of birth;
2. practice code;
3. child and parent's unique identifier codes;
4. date and time of randomisation;
5. intervention allocation (message type and timing);
6. date and time of intervention SMS sent if sent;
7. date and time of any subsequent index vaccination at that clinic within 90 days after randomisation.
8. Parent postcode
9. Date vaccine due
10. SMS delivery failure (if applicable)

For each scheduled vaccination, the closest vaccination occasion in time will be obtained from SmartVax and the relative timing of the vaccination will be calculated as the difference between the date of vaccine administration and the scheduled due date.

Vaccinations occurring later than 42 days after the scheduled due date will be right-censored. Vaccinations occurring earlier than 14 days before the scheduled due date will be excluded. Therefore, the time to vaccination in days will range from -14 to 42 days. Vaccination status at 28 days will be calculated as the proportion of participants with a time to vaccination between -14 to 28 days relative to the scheduled due date.

6.2 Analysis of primary outcome

For each arm and overall, the number of assigned participants and the raw count and proportion amongst those participants vaccinated with 28 days will be reported.

Inferences for the primary outcome will be based on a Bayesian logistic regression model. The model will be used to estimate the log-odds of vaccination by 28 days (primary endpoint) following the scheduled due date for the interventions. The model will account for variation in outcomes by:

- intervention effect (timing by framing combination)
- scheduled age of vaccination at 2,4,6,12,18, and 48 months of age (fixed categorical effects).
- clinic attended (random effect)
- calendar time of vaccine due date (grouped into epochs of 4 weeks and smoothed across groups).

The factorial design of the intervention arms in terms of SMS message framing and timing is presented in Table 1 with cell labels for each framing-timing combination.

Table 1: Intervention summary

	Arm	Timing	Framing	Combination
Control	0	0	0	a_0b_0
Intervention 1	1	1	1	a_1b_1
Intervention 2	2	1	2	a_1b_2
Intervention 3	3	1	3	a_1b_3
Intervention 4	4	1	4	a_1b_4
Intervention 5	5	2	1	a_2b_1
Intervention 6	6	2	2	a_2b_2
Intervention 7	7	2	3	a_2b_3
Intervention 8	8	2	4	a_2b_4
Intervention 9	9	3	1	a_3b_1
Intervention 10	10	3	2	a_3b_2
Intervention 11	11	3	3	a_3b_3
Intervention 12	12	3	4	a_3b_4

In what follows, the message timings are denoted by a and message framings by b. The interim analyses are designated by $t = 1, ..., T$ and cohort t refers to individuals recruited between interim $t-1$ and t which has sample size n_t. The total sample size at analysis t is then $N_t = \sum_{j=1}^{t} n_t$. We denote all data available at analysis t by D_t.

The model for the log-odds of vaccination at 28 days after the scheduled due date, denoted η, and the associated probability, p, are modelled by

$$\begin{aligned}\eta_i &= \alpha + l_i\zeta + x_{ia}^{\mathsf{T}}\gamma_a + x_{ib}^{\mathsf{T}}\gamma_b + x_{iab}^{\mathsf{T}}\gamma_{ab} + w_i^{\mathsf{T}}\beta + z_{i\xi}^{\mathsf{T}}\xi + z_{i\tau}^{\mathsf{T}}\tau \\ p_i &= \text{logit}^{-1}(\eta_i).\end{aligned}$$

- The α term is the intercept parameter giving the average log-odds of vaccination for the control arm.
- The ζ term is the average intervention effect relative to the control arm across all message framings and timings.
- The γ_a constrained to sum to zero, are the deviation effects for the message timings from the average intervention effect.
- The γ_b constrained to sum to zero, are the deviation effects for the message framings from the average intervention effect.
- The γ_{ab} constrained to sum to zero on the relevant message and timing margin, are the interaction effects between message framing and timing.
- The β are other model parameters for the model covariates: scheduled age of vaccination.

- The ξ and τ are random effect terms adjusting for clinic and calendar due date respectively.

The prior distributions for the intercept and average intervention effect parameters will be set to

$$\alpha \sim N(\text{logit}^{-1}(0.8), 2.5)$$
$$\zeta \sim N(0, 1)$$

noting that we expect the proportion vaccinated by 28 days to be closer to 1 than 0.

The priors on the γ terms will be set so as to enforce the relevant sum-to-zero constraints and be permutation invariant

$$S_l = \mathrm{I}_l - l^{-1}\mathrm{J}_l$$
$$\gamma_a \sim N(0, S_4)$$
$$\gamma_b \sim N(0, S_3)$$
$$\gamma_{ab} \sim N(0, S_4 \otimes S_3).$$

The prior on other model coefficients will be

$$\beta \sim N(0, 2.5^2).$$

The use of response adaptive randomisation means that allocation ratios will change over time. If background trends in the outcome are also present, then arms with higher allocations over certain periods may display larger or smaller effects than those attributable to the intervention. Therefore, the two random effects terms are included, one for clinic, and one for epoch. These will be modelled as

$$\xi|\sigma_x i^2 \sim N(0, \sigma_\xi^2)$$
$$\sigma_\xi^2 \sim \text{Half-}t(3, 0, 1)$$
$$\tau_e|\sigma_\tau^2 \sim N(\tau_{e-1}, \sigma_\tau^2)$$
$$\sigma_\tau^2 \sim \text{Half-}t(3, 0, 1)$$
$$\tau_1 = 0.$$

where e indicates the epoch. The parameter τ has a first-order random walk prior to smooth baseline changes in the response across epoch's. The epoch groupings will be in 28 day increments with $\tau_1 = 1$ representing the most recent 28 days. In the event that accrual is slower then expected, this grouping may need to be reviewed.

Pre-specified adaptations will be assessed using the primary analysis model.

6.3 Sensitivity analyses of primary outcome

As sensitivity analyses, reduced versions of the primary analysis model will be estimated. A reduced model is specified without adjustment for clinic and epoch. Another reduced model is specified without the interaction term between framing and timing, and also without the framing and timing effects themselves for a completely pooled model comparing control to intervention.

The reduced models considered for comparison will be

$$\begin{aligned}
\eta &= 1\alpha + L\zeta + W\beta + Z_\xi \xi + Z_\tau \tau \\
\eta &= 1\alpha + L\zeta + X_a\gamma_a + X_b\gamma_b + W\beta + Z_\xi \xi + Z_\tau \tau \\
\eta &= 1\alpha + L\zeta + X_a\gamma_a + X_b\gamma_b + X_{ab}\gamma_{ab} + W\beta.
\end{aligned}$$

The same priors on common parameters as for the primary analysis will be used in these reduced models.

6.4 Statistical quantities

The posterior distribution of the model parameters will be approximated using Markov chain Monte Carlo methods via the Stan language. At least 10,000 draws will be generated from the joint posterior distribution for the primary analysis, and these draws will be used to approximate the quantities of interest as outlined in the current section and Section 6.6.

For each of the 13 arms, we define μ_j as the conditional log-odds of vaccination by 28 days for arm $j = 0, ..., 12$ and define $\theta_{jk} = \mu_j - \mu_k$ as the pairwise differences for $j, k = 0, ..., 12$.

6.4.1 Posterior summaries

At the final analysis, posterior summaries for the model parameters will be presented in terms of point estimates and highest density intervals. These values will summarise: the average intervention effect across all message framing and timings relative to control, the average effect of each framing and timing relative to the overall intervention effect and control, the main effect of each framing and timing, and interaction effects of the framing and timing combinations. Due to the potential to drop the control arm, comparisons will also be made with the current most probably best arm as defined in the next section. Where explicit hypotheses and decision rules have been stated, these will be assessed and reported in terms of their posterior probability as outlined below.

6.4.2 Probability an intervention arm is best

Decisions related to the primary outcome and response adaptive randomisation will be based on the posterior probability that each arm is superior to all others. We define π_{jt} be the posterior probability an intervention

$j = 1, ..., 12$, is the best intervention given the data available up to interim t. The set of interventions to be included in the comparison is only those interventions which are still active (those not found to be worse than control). This set of interventions is denoted $\mathcal{A}_t$. If $\mathcal{A}_t = \emptyset$, that is, there are no active interventions, then the trial will be stopped. The value is

$$\pi_{jt} = \mathbb{P}[\mu_j > \mu_k; \forall k \neq j, k, j \in \mathcal{A}_t | D_t].$$

The value of π_{jt} for any $j \notin \mathcal{A}_t$ is defined to be 0. The **current most probably best** intervention arm is then chosen to be intervention arm which satisfies $b_t = \arg\max_j \pi_{jt}$.

6.4.3 Probability an intervention arm is better than control

To declare an intervention arm superior, it must also be better than standard of care. Let ϕ_{jt} be the posterior probability that each intervention $j = 1, ..., 12$, is better than control given the data available to interim t, given by

$$\phi_{jt} = \mathbb{P}[\mu_j > \mu_0 | D_t].$$

6.4.4 Probability of beneficial average intervention effect

There may be insufficient precision to declare any single intervention better than control, but there may be strong evidence that on average receiving an intervention is better than not. The probability that an intervention is on average beneficial is

$$\varphi_t = \mathbb{P}[\alpha_1 > 0 | D_t].$$

6.4.5 Probability of Rank

At the end of the trial, we may not have declared any intervention arm superior. There may still be a subset of intervention arms which are competing amongst each other, but standout as superior to the others. Investigation of rank probabilities may help with decision making in such a scenario.

At each interim, the cell means and factor level means may be ranked according to (in terms of cell means as an example)

$$R_j = \text{rank}(\mu_j) = \sum_{l=1}^{12} \mathbb{I}[\mu_j \geq \mu_l]$$
$$R_j^\star = \text{rank}\left(\mathbb{E}[R_j | D_t]\right).$$

For each intervention arm and main effect, we will investigate the marginal rank probabilities $\mathbb{P}[R_j = k | D_t]$, that is, the probability that arm j is ranked kth in response rate.

Clusters of ranked arms may be identified by collections which have high probability of exceeding a given rank and near-zero probability of being below this rank. For example, four arms may each have probability 1 of rank at least 4 and 0 of rank less than 4 indicating that these stand-out as the top 4 interventions even though there is insufficient information to declare any one of those 4 best overall.

6.5 Analysis of secondary outcomes

6.5.1 Secondary Outcome 1. - Time-to-event

For the secondary outcome definitions refer to Section 3.3.

For each arm and overall, the empirical hazard and survival for days to vaccination will be reported.

Inferences for the time to vaccination outcome will be based on a proportional continuation ratio model (logistic link). Primary interest lies in the treatment effect on the conditional log-odds of vaccination at a given time (or the corresponding conditional odds ratio).

Events may occur at times $k \in \{1, ..., K\}$ with $y_{ik} = 1$ denoting vaccination and $y_{ik} = 0$ no vaccination. We take $k = 1$ to be 14 days before the due date of the index vaccination. SMS messages may then be sent at times $k = 1, 15, 22$ (14 days before due, on the due date, and 7 days after the due date, respectively). Censoring at 42 days after the due date then occurs at $k = 57$.

The interventions themselves are time-varying; the message cannot affect the outcome until it has been sent. All participants contribute to the baseline hazard until the their intervention becomes active. The intervention design matrix are the same as for the primary outcome. If $\tau_i \in \{1, 15, 22\}$ denotes the timing of the intervention for participant i, then

$$u_{ik} = \mathbf{1}_{[\tau_i,\infty)}(k)$$

indicates whether the intervention is active at time k for participant i.

We specify a semi-parametric hazard regression model for flexible modelling of the baseline (control group) hazard with the curve modelled by penalised O'Sullivan splines (Currie and Durban, 2002; Eilers and Marx, 1996; Wand and Ormerod, 2008)

$$\begin{aligned}
\eta_{ik} &= s_{0k} + u_{ik}\left[l_i\zeta + x_{ia}^{\mathsf{T}}\gamma_a + x_{ib}^{\mathsf{T}}\gamma_b + x_{iab}^{\mathsf{T}}\gamma_{ab}\right] + w_i^{\mathsf{T}}\beta + z_{\xi,i}^{\mathsf{T}}\xi + z_{\tau,i}^{\mathsf{T}}\tau \\
\lambda_{ik} &= \text{logit}^{-1}(\eta_{ik}) \\
s_{0k} &= \alpha_0 + \alpha_1 k + \textstyle\sum_{m=1}^{M} b_m B_{mk} \\
b_m|\sigma^2 &\sim N(0, \sigma^2) \\
\sigma &\sim \text{Half-}t(3, 0, 10) \\
\alpha &\sim N(0, 2.5^2).
\end{aligned}$$

where B_m are spline bases enforcing the penalty and priors on the other parameters are as in the primary analysis model. We specify 28 equally spaced interior knots to be used for the baseline hazard.

6.5.1.1 Sensitivity Analyses It may be that the assumption of proportional continuation ratio is inappropriate. As sensitivity analysis, expanded versions of the model will be investigated. These models will allow interventions effects to be flexibly varying on the conditional log-odds of vaccination. Under these models, the intervention specific curves are specified as

$$\eta_{ik} = s_{0k} + u_{ik}\left[s_{j(i),k}\right] + w_i^\mathsf{T}\beta + z_{\xi,i}^\mathsf{T}\xi + z_{\tau,i}^\mathsf{T}\tau$$

where the group specific term is modelled by either

$$\begin{aligned}
(1) \quad s_{jk} &= \gamma_{0j} + \gamma_{1j}k \\
(2) \quad s_{jk} &= \gamma_{0j(a)} + \gamma_{1j(a)}k + \textstyle\sum_{m=1}^{M} g_{j(a)m}B_{mk} \\
\gamma &\sim N(0, 2.5^2) \\
g_{jm}|\omega_j^2 &\sim N(0, \omega_j^2) \\
\omega_j &\sim \text{Half-}t(3, 0, 10).
\end{aligned}$$

where $j(a)$ returns the timing of intervention j. That is (1) represents combination specific curves and (2) timing specific curves combined across all message framings.

Under these models, the proportional continuation ratio does not apply. Therefore, primary interest will be in the survival curves themselves and restricted mean survival time (RMST) (Royston and Parmar, 2013, 2011) to $t^\star$ in each group, defined as

$$\text{RMST}_j(k^\star) = \int_0^{k^\star} S_j(k)\,\mathrm{d}k, \quad j = 0, 1, ..., 12.$$

with $S_j(k)$ the survival curve of remaining unvaccinated obtained from the above models for group j. In particular, we will compare groups according to the difference in their RMST relative to the baseline (no intervention) survival

$$\Delta_j(k^\star) = \int_0^{k^\star} S_j(k) - S_0(k)\,\mathrm{d}k, \quad j = 1, ..., 12.$$

as a summary of the relative average number of days spent unvaccinated.

6.5.2 Secondary Outcomes 2. and 3.

Secondary outcome 2. and 3. will be analysed analogously to the primary and first secondary outcome. However, nesting of vaccine occasions within parents will be accounted for by an additional hierarchical component for the parent in the linear predictor.

6.6 Interim analyses and trial adaptations

A first interim analysis is scheduled to occur when 1,500 index vaccinations reach the primary endpoint. Subsequent interim analyses are scheduled to occur every additional 500 index vaccinations which reach the primary endpoint. Only the primary analysis will be performed at each interim and thus only the primary outcome will inform the response-adaptive randomisation and trial adaptations. At each interim analysis there will be index cases who are enrolled but yet to reach 28 days after their due vaccination; these are not eligible for inclusion in the current interim analysis but will be included in subsequent interims once their 28 days has passed. With the maximum sample size of 10,000, a first interim analysis at 1,500, and interims every 500 participants implies a maximum of 18 interim analyses.

6.6.1 Response Adaptive Randomisation

The control arm will have fixed allocation of $q_0 = 1/5$ throughout the duration of the trial unless dropped in which case $q_0 = 0$. This fixed allocation applies regardless of the number of active intervention arms. The higher allocation to control was chosen to increase power on the multiple comparisons of each intervention to control for effectiveness.

At the start of the trial, allocation ratios to each intervention arm will be equal to $\frac{4}{5} \times \frac{1}{12}$. The randomisation probabilities to the intervention arms will be updated is data accrues.

Following each analysis t, the allocation probability to arm j will be a function of the probability that each arm is the best intervention arm in proportion to

$$r_{jt} \propto \sqrt{\frac{\pi_{jt}\mathbb{V}[\mu_j|D_t]}{n_{jt}+1}}, \quad j = 1, ..., 12.$$

The ratios are normalised to sum to one and share the remaining probability after accounting for the fixed control arm allocation q_0

$$q_{jt} = (1 - q_0)\frac{r_{jt}}{\sum_j r_{jt}}, \quad j = 1, ..., 12.$$

An intervention arm may be permanently dropped if there is evidence it is worse than no intervention. If $\hat{\phi}_{jt} < \kappa_t^{\text{harmful}}$ then $q_{jt} = 0$ and it's allocation mass is redistributed amongst the remaining intervention arms.

6.6.2 Intervention effectiveness and superiority

At any analysis, if a single arm has at least $\kappa_t^{\text{effective}}$ posterior probability of being better than control then the control arm will be dropped. Additionally, if on average receiving an intervention is more effective than

control, then the control arm will be dropped. The dropping rule for the control arm is,

$$\left(\exists j \in \{1, ..., 12\} : \hat{\phi}_{jt} > \kappa_t^{\text{effective}}\right) \text{ or } \left(\hat{\varphi}_t > \kappa_t^{\text{effective}}\right).$$

At any analysis, if a single arm has at least $\kappa_t^{\text{superior}}$ posterior probability of being the best overall arm, and this arm is better than the control group with at least $\kappa_t^{\text{effective}}$ posterior probability, then this result triggers a stopping of the trial for superiority of that arm. The stopping rule is,

$$\exists\, j \in \{1, ..., 12\} : \hat{\pi}_{jt} > \kappa_t^{\text{superior}} \text{ and } \hat{\phi}_{jt} > \kappa_t^{\text{effective}}.$$

6.6.3 Harmful interventions

If at any interim analysis an intervention is found to be harmful, then that intervention will be dropped. The dropping rule for intervention arm j is

$$\hat{\phi}_{jt} < \kappa_t^{\text{harmful}},\ j = 1, ..., 12.$$

If all arms are worse than control then the trial may be stopped early for lack of effectiveness of any intervention relative to standard care. Alternatively, if on average receiving an intervention is worse than control the trial may be stopped for lack of effectiveness. The decision rule is

$$\left(\hat{\phi}_{jt} < \kappa_t^{\text{harmful}} \text{ for all } j \in \{1, ..., 12\}\right) \text{ or } \left(\hat{\varphi}_t < \kappa_t^{\text{harmful}}\right).$$

6.6.4 Thresholds

Due to the number of arms and the number of interims, the thresholds are set to relatively strict values to account for multiplicity. The thresholds are also allowed to reduce as information accrues to balance the risk of making a decision based on less information. The threshold to be used in the interim analyses are

$$\begin{aligned}
\kappa_t^{\text{harmfjul}} &= 0.01\sqrt{\frac{N_t}{10,000}} \\
\kappa_t^{\text{superior}} &= 0.95 - 0.25\sqrt{\frac{N_t}{10,000}}, \quad t = 1, ..., 17, \\
\kappa_t^{\text{effective}} &= 0.99^{\sqrt{\frac{N_t}{10,000}}}
\end{aligned}$$

where N_t is the sample size at the interim analysis.

6.6.5 Summary of Interim Analyses

The following summarises the adaptation process at each interim analysis.

- The primary analysis model is updated conditional on the available data as outlined in Section 6.2.
- The posterior summaries and statistical quantities are calculated as outlined in Section 6.4.
- The decision rules are evaluated as outlined in Section 6.6 and Table 2.
- If the trial has not stopped, the allocation probabilities are updated as in Section 6.6.1.

Table 2: Interim analysis, t, decision rule summary

Comparison	Criteria	Decision	Action
j vs. control	$\phi_{jt} > \kappa_t^{\text{effective}}$	Intervention effective	Drop control arm
avg vs. control	$\varphi_t > \kappa_t^{\text{effective}}$	On average effective	Drop control arm
j vs. control	$\phi_{jt} < \kappa_t^{\text{harmful}}$	Intervention harmful	Drop intervention j
avg vs. control	$\varphi_t < \kappa_t^{\text{harmful}}$	On average harmful	Stop trial
all vs. control	$\mathcal{A}_t = \emptyset$	No active interventions	Stop trial
j vs. all	$\pi_{jt} > \kappa_t^{\text{superior}} \cap \phi_{jt} > \kappa_t^{\text{effective}}$	Intervention superior	Stop trial

6.7 Subgroup analyses

The effect of SMS vaccine reminders on the primary endpoint will be investigated in the final analysis for the following subgroups:

- Scheduled age for vaccination (2, 4, 6, 12, 18 months and 48 months)

6.8 Missing data

Randomisation will only occur for participants with complete baseline data (all data fields except for the date of index vaccination). The SV software will include basic logic checks to prohibit entry of nonsensical or internally inconsistent data. Children with missing date of index vaccination data after 42 days since randomisation will be assumed to have not received the index vaccine before that date. No attempt will be made to confirm the practice-entered data with the practice or to ascertain or verify from other sources.

6.9 Software

Data processing will be performed using R. Models will be fit in R using Stan via the `rstan` package.

7 Operating Characteristics

Given the trial adaptations, simulations were required to explore and assess the operating characteristics of the design. Extensive simulations were undertaken where the trial design parameters were varied and trial quantities of interest were investigated.

The choice of the trial parameters and thresholds aims to achieve the following:

- ≈ 0.25 probability of dropping the control arm in the null scenario, $\mu_0 = \mu_1 = ... = \mu_{12}$
- ≈ 0.05 probability of declaring any single intervention superior at stopping in the null scenario
- ≈ 0.10 probability of declaring any single intervention effective at stopping in the null scenario

Later dropping/stopping times were preferred to earlier times, therefore the thresholds were scaled with the information ratio.

For efficiency, simulations utilised variational approximations to the model parameter posteriors, and posterior quantities were calculated on the basis of this approximation. Operating characteristics were determined by conducting 10,000 simulations under each scenario. The trial parameters as outlined in this statistical analysis plan were chosen based on the results from these simulations.

The following does not include all simulations conducted, but rather only those under the chosen parameterisation.

7.1 Simulation scenarios

Although additional simulation scenarios were considered, included in this document are results under the following scenarios. Effect sizes of odds ratios range from $1/1.2$ to 1.5 by increments of 0.1. Under each effect size we consider:

- all interventions are modified by effect size,
- one intervention is modified by effect size,
- one message timing is modified by effect size,
- one message framing is modified by effect size.

The simulations are summarised with a focus on the timing of the decision rules being triggered and the posterior summaries at trial stopping.

7.2 Simulation results

7.2.1 All equally effective

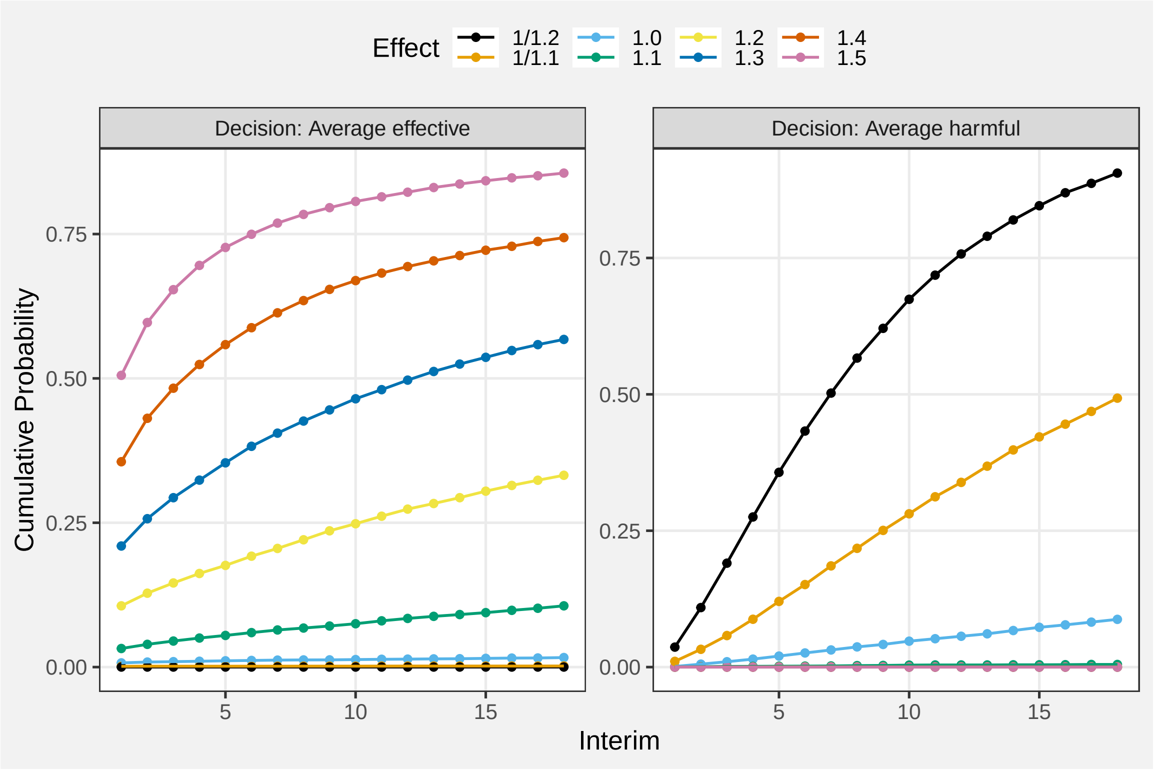

Figure 1: Decision times for average intervention effect.

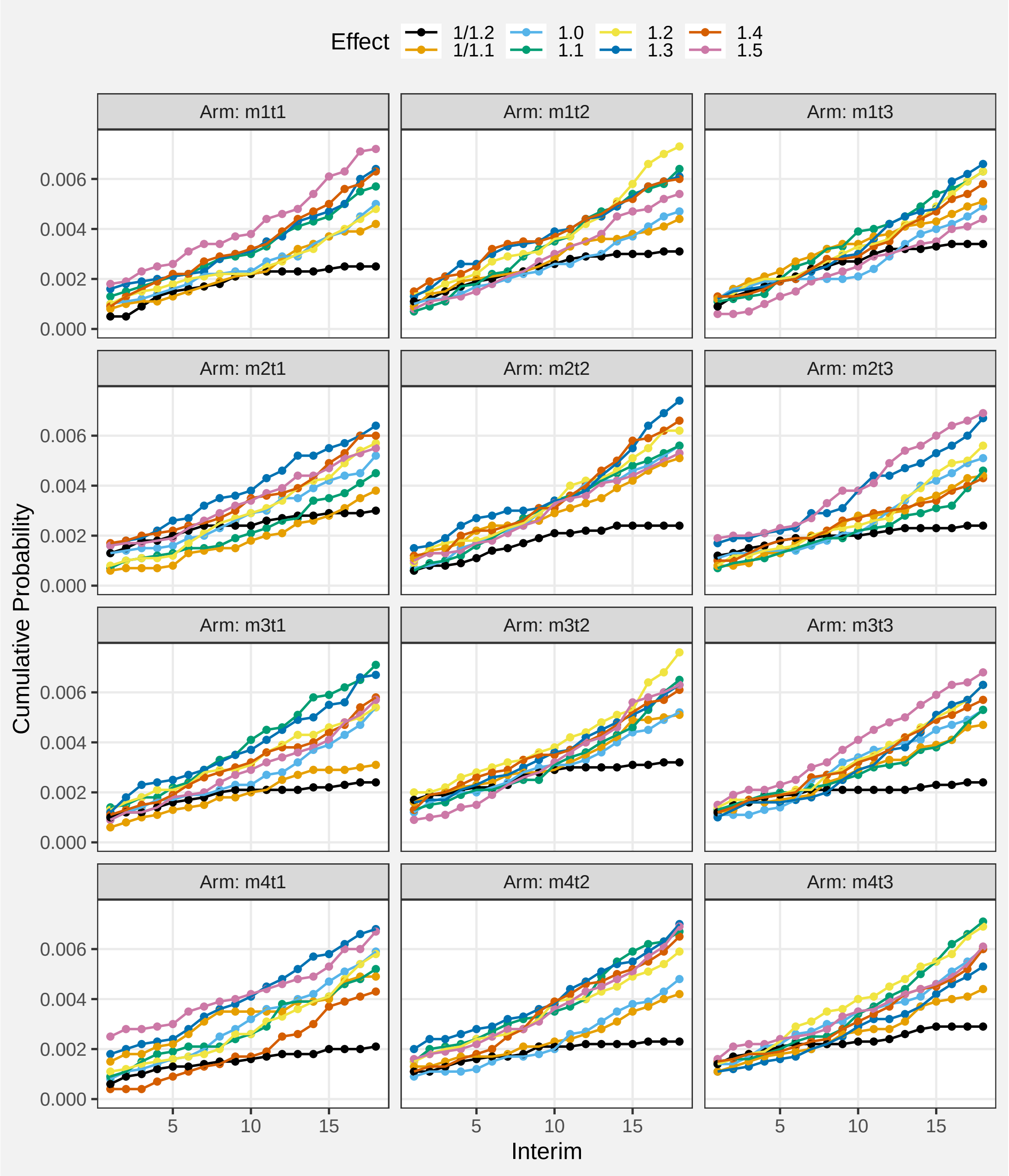

Figure 2: Decision times for superiority of each intervention.

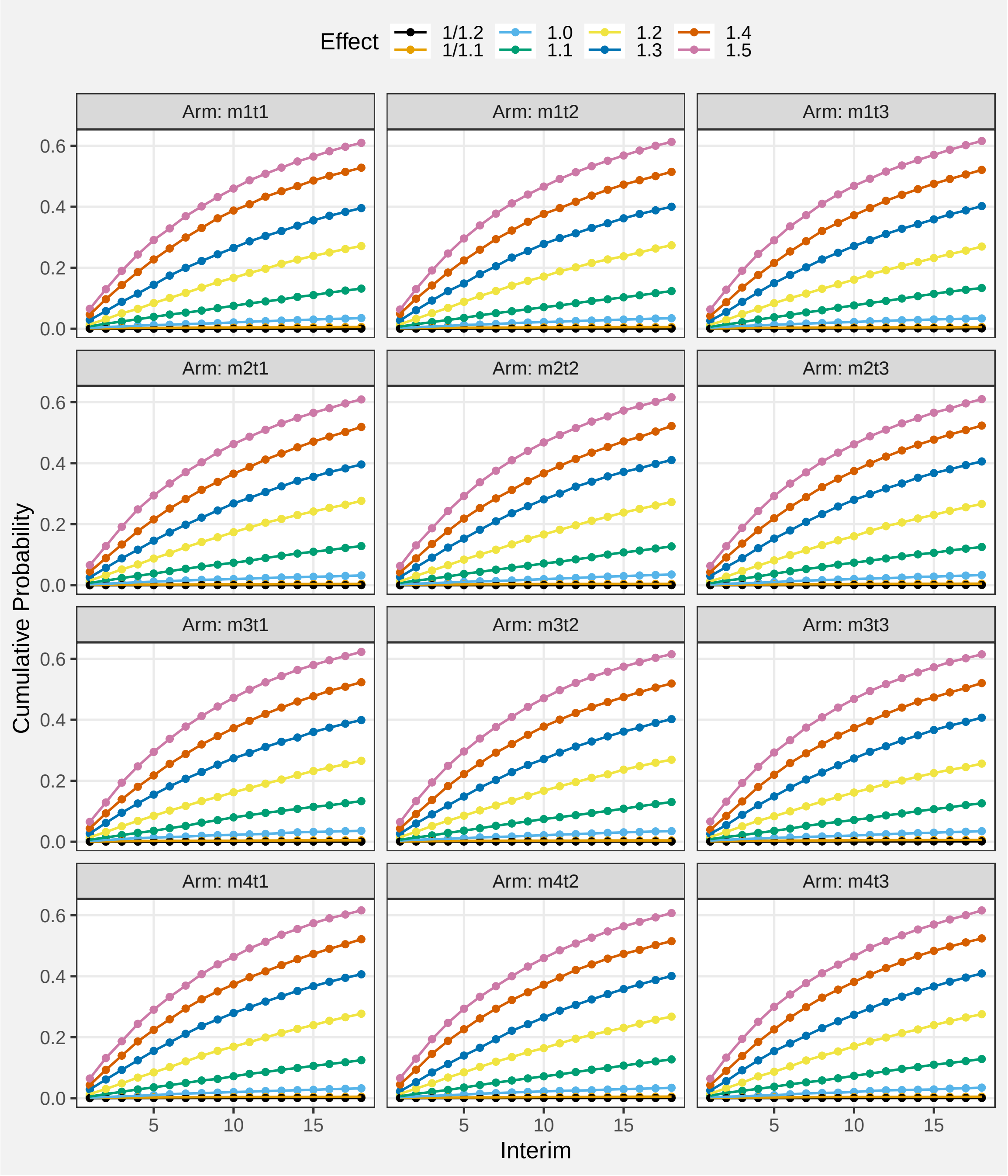

Figure 3: Decision times for effectiveness of each intervention.

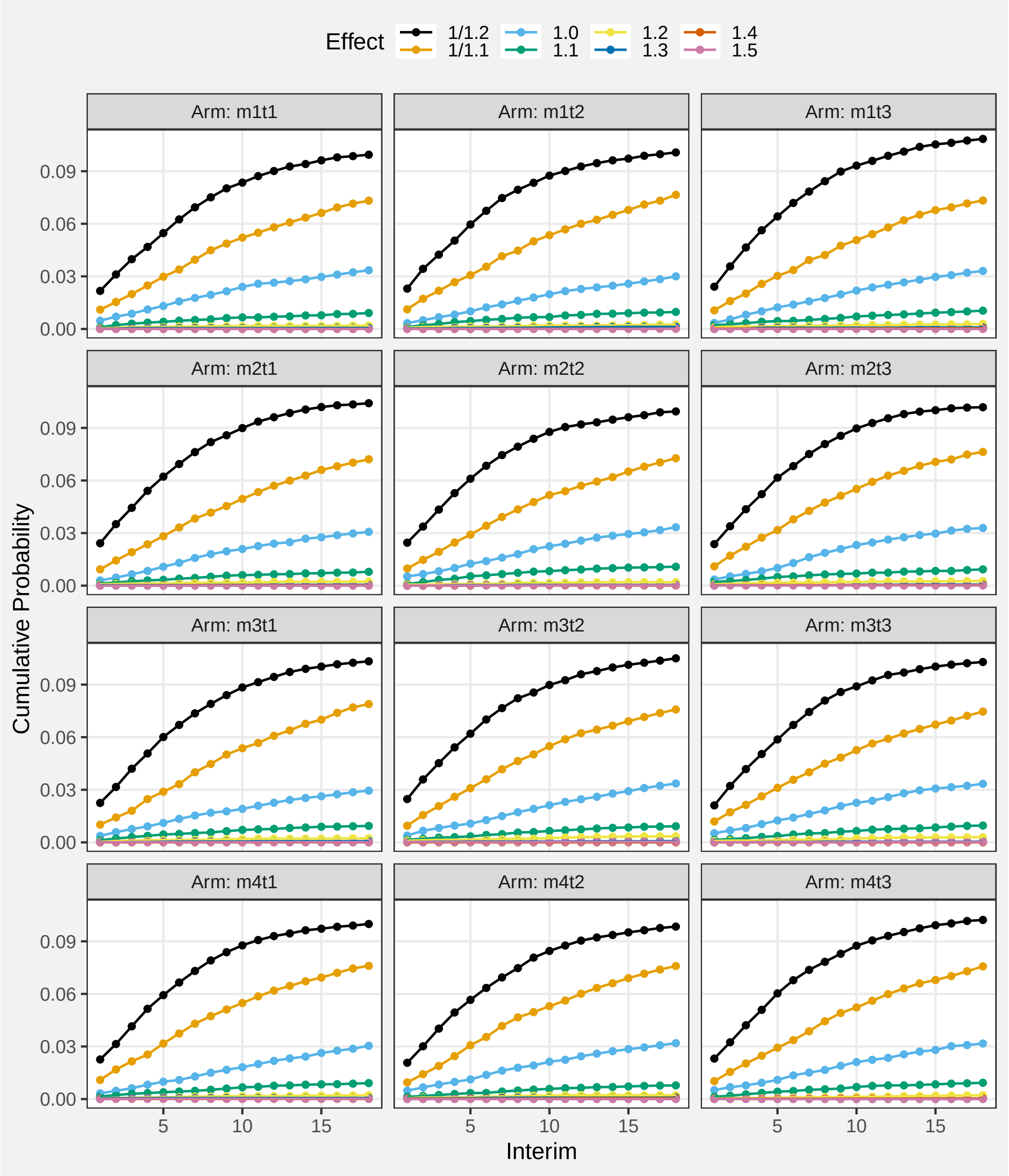

Figure 4: Decision times for harmfulness of each intervention.

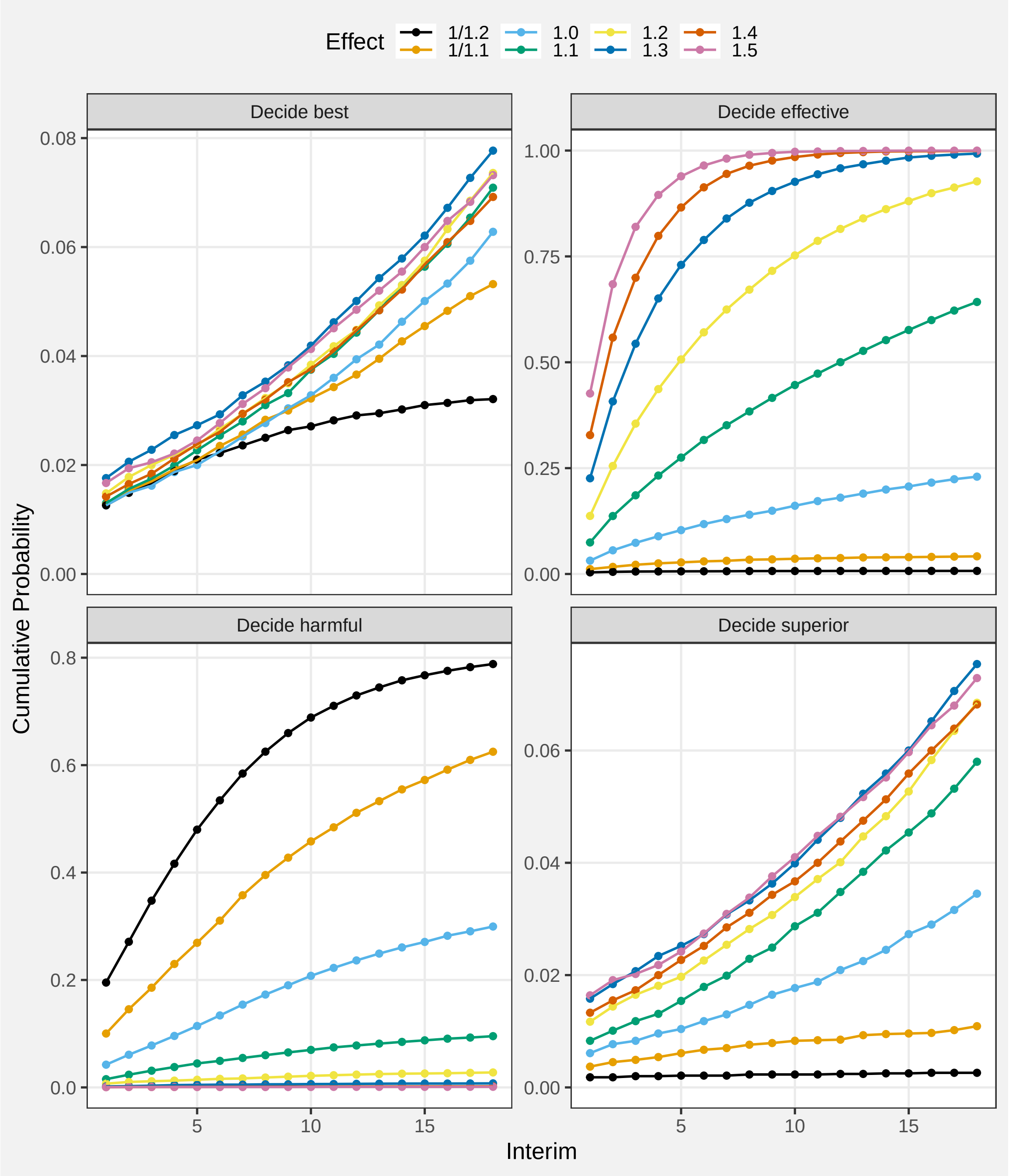

Figure 5: Earliest decision times across all intervention arms.

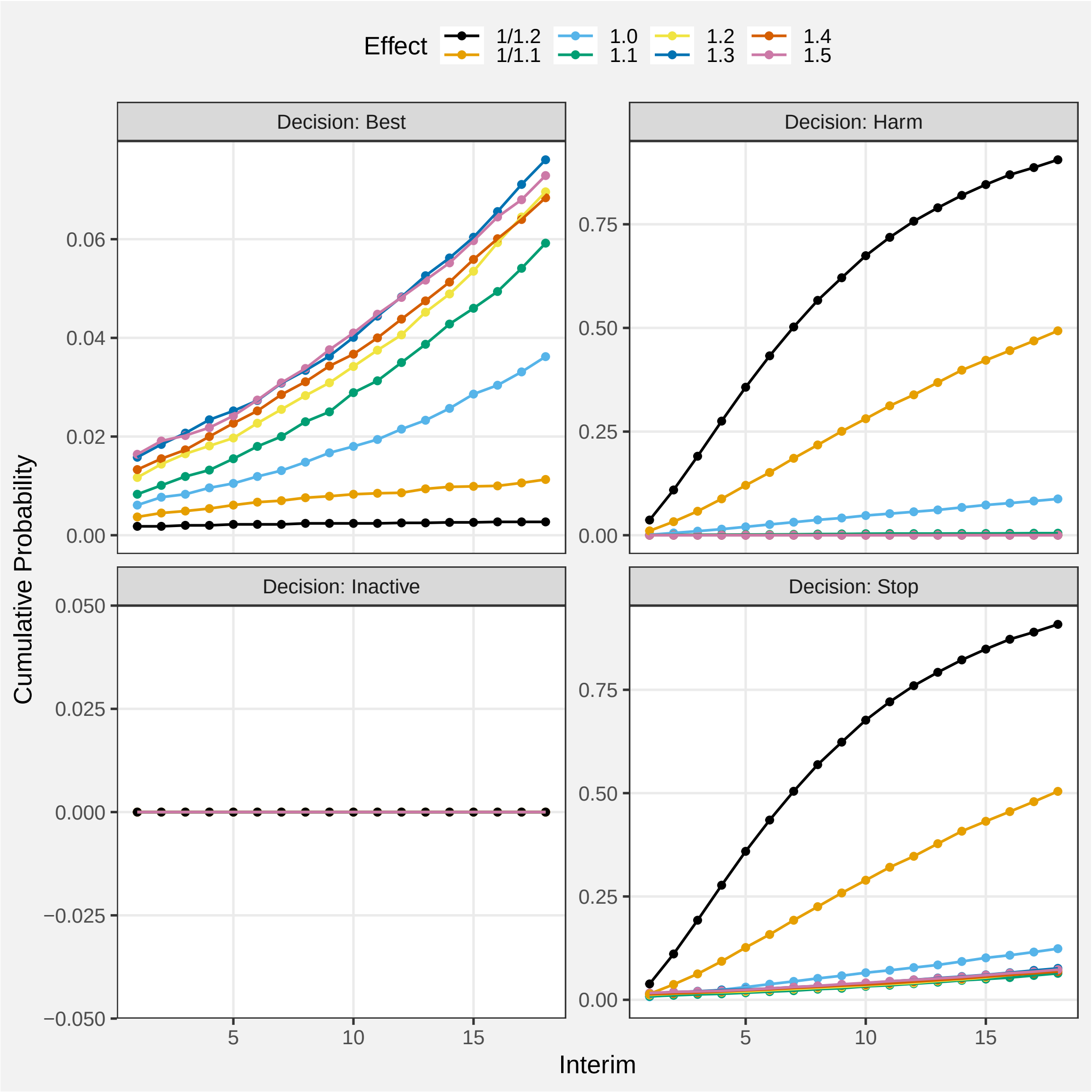

Figure 6: Stopping times and reason for stopping.

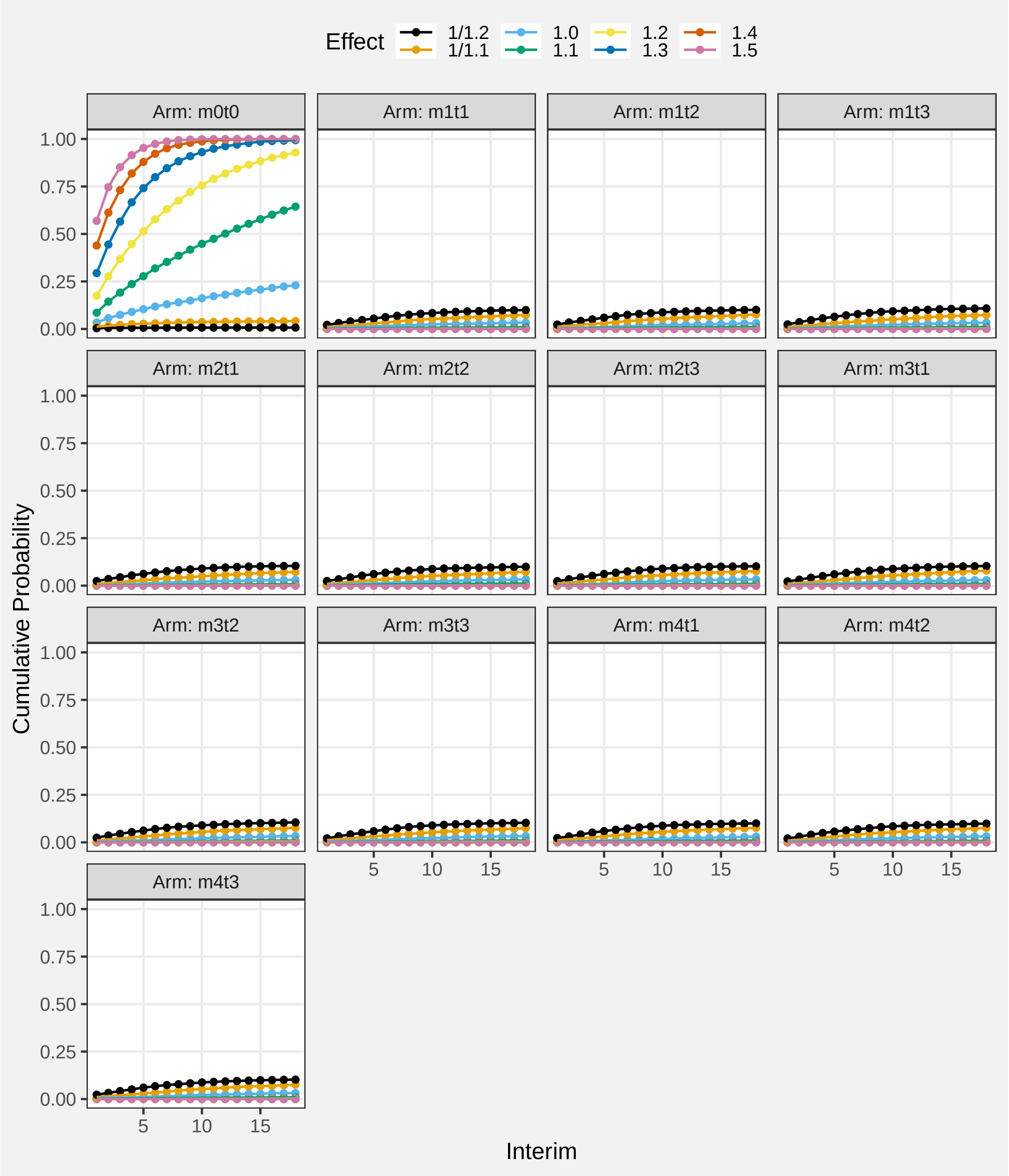

Figure 7: Decision times for dropping each intervention arm.

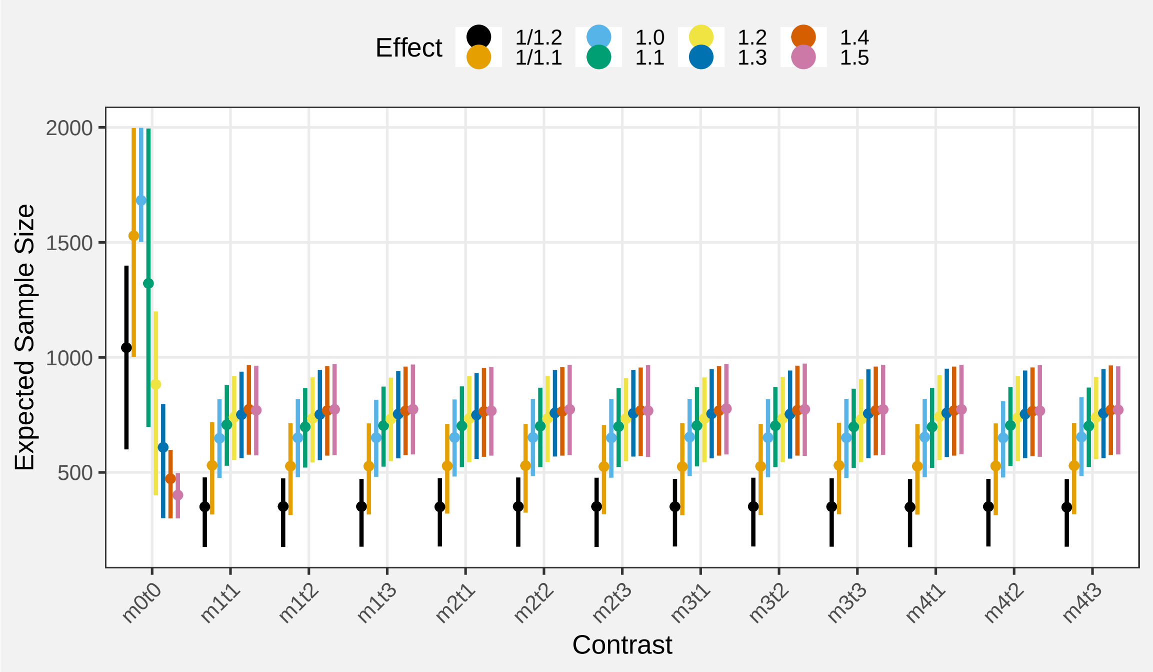

Figure 8: Expected sample size by arm.

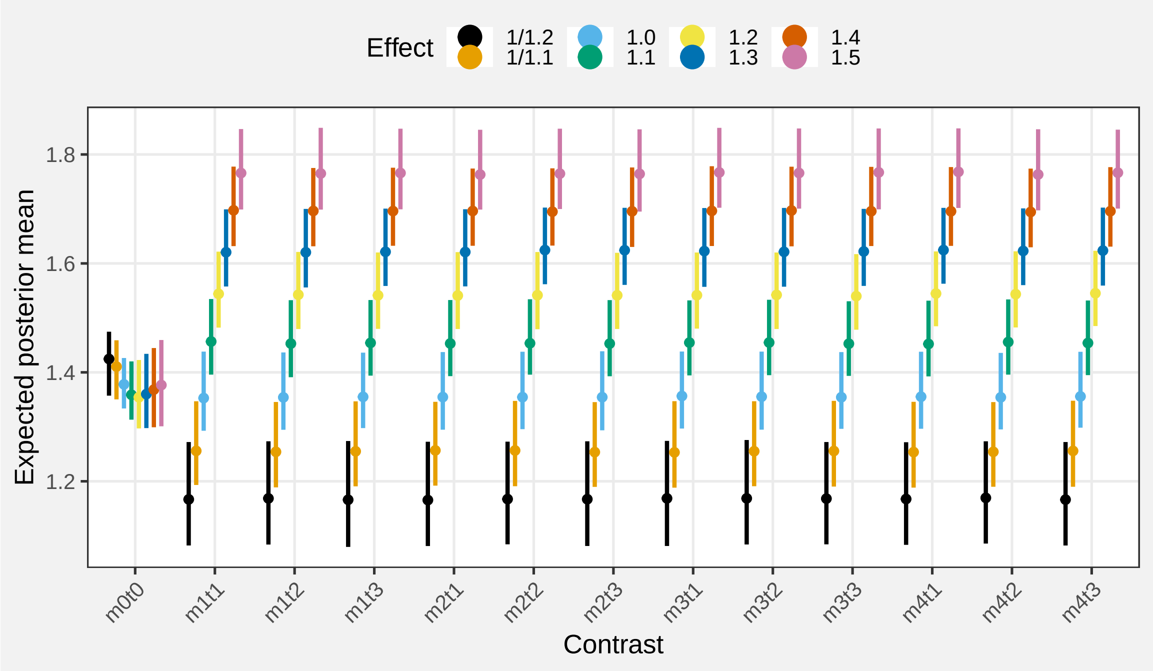

Figure 9: Expected posterior mean by arm.

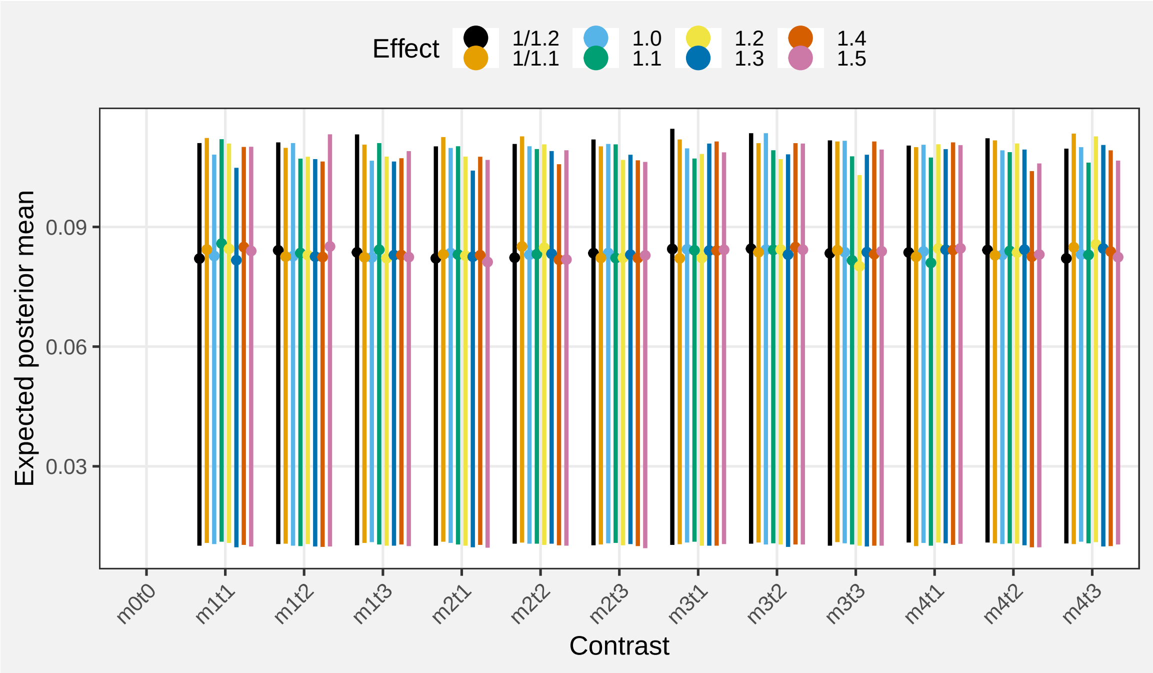

Figure 10: Expected posterior probability of superiority by arm.

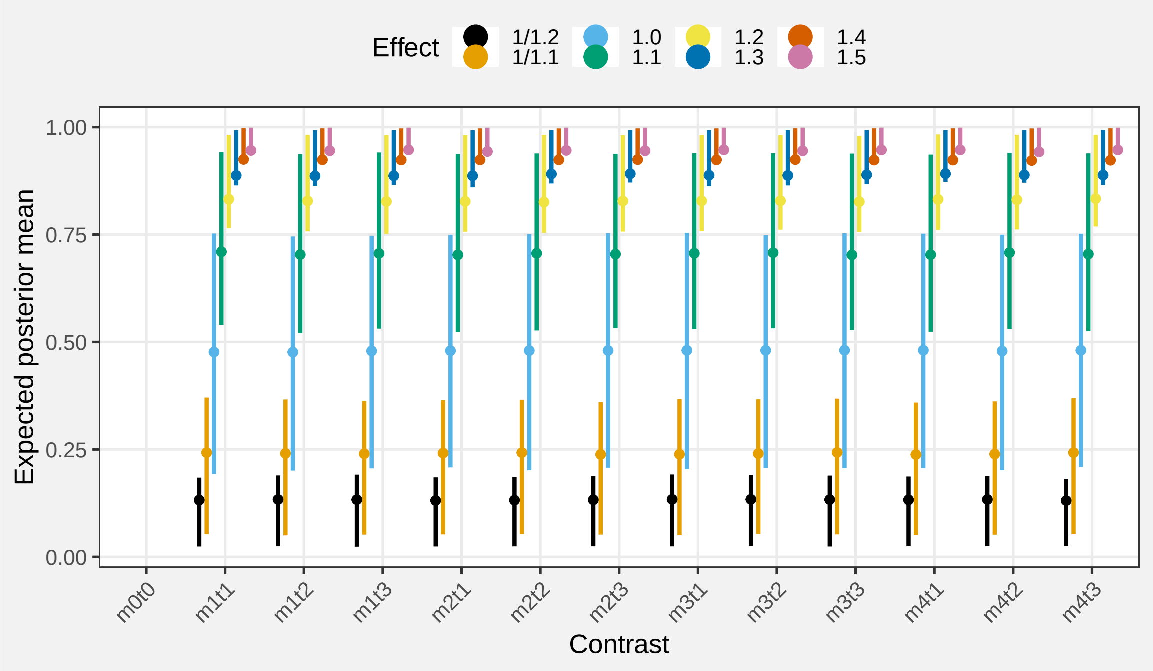

Figure 11: Expected posterior probability of effectiveness by arm.

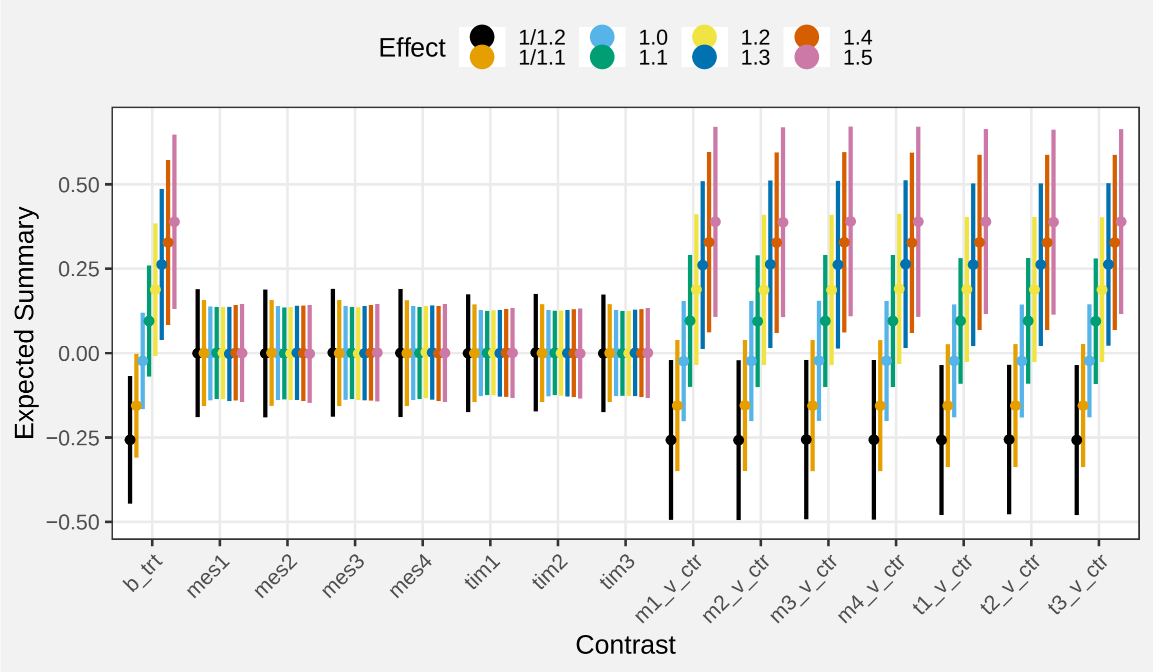

Figure 12: Expected posterior summary of contrasts.

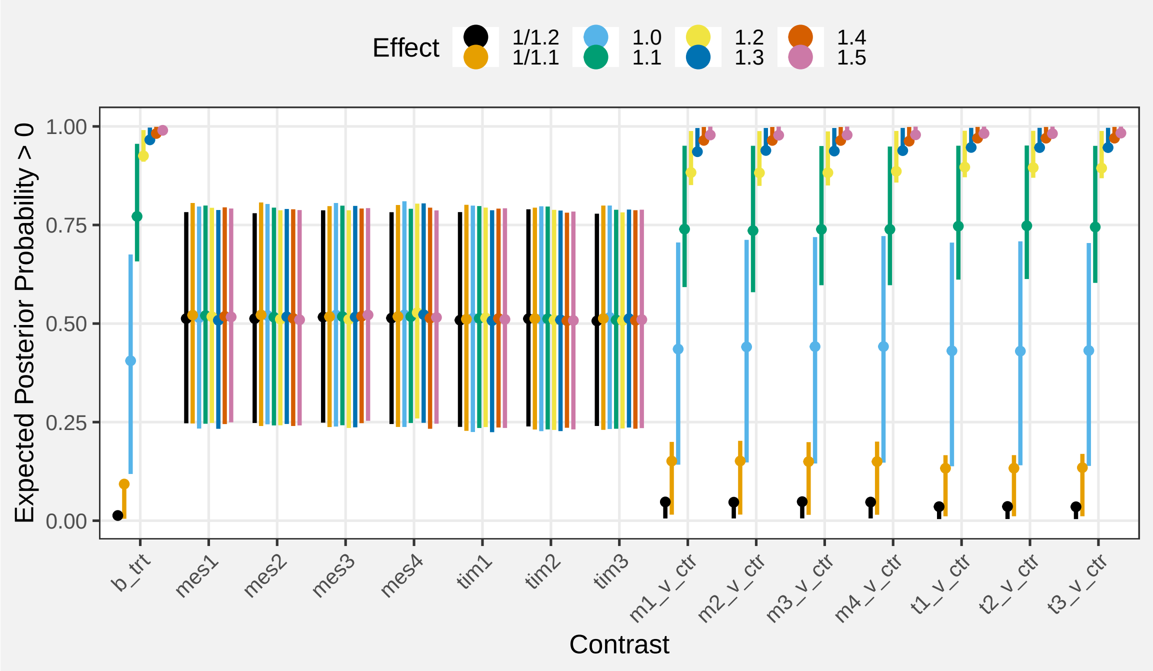

Figure 13: Expected posterior probability of contrast effect.

7.2.2 One intervention effective

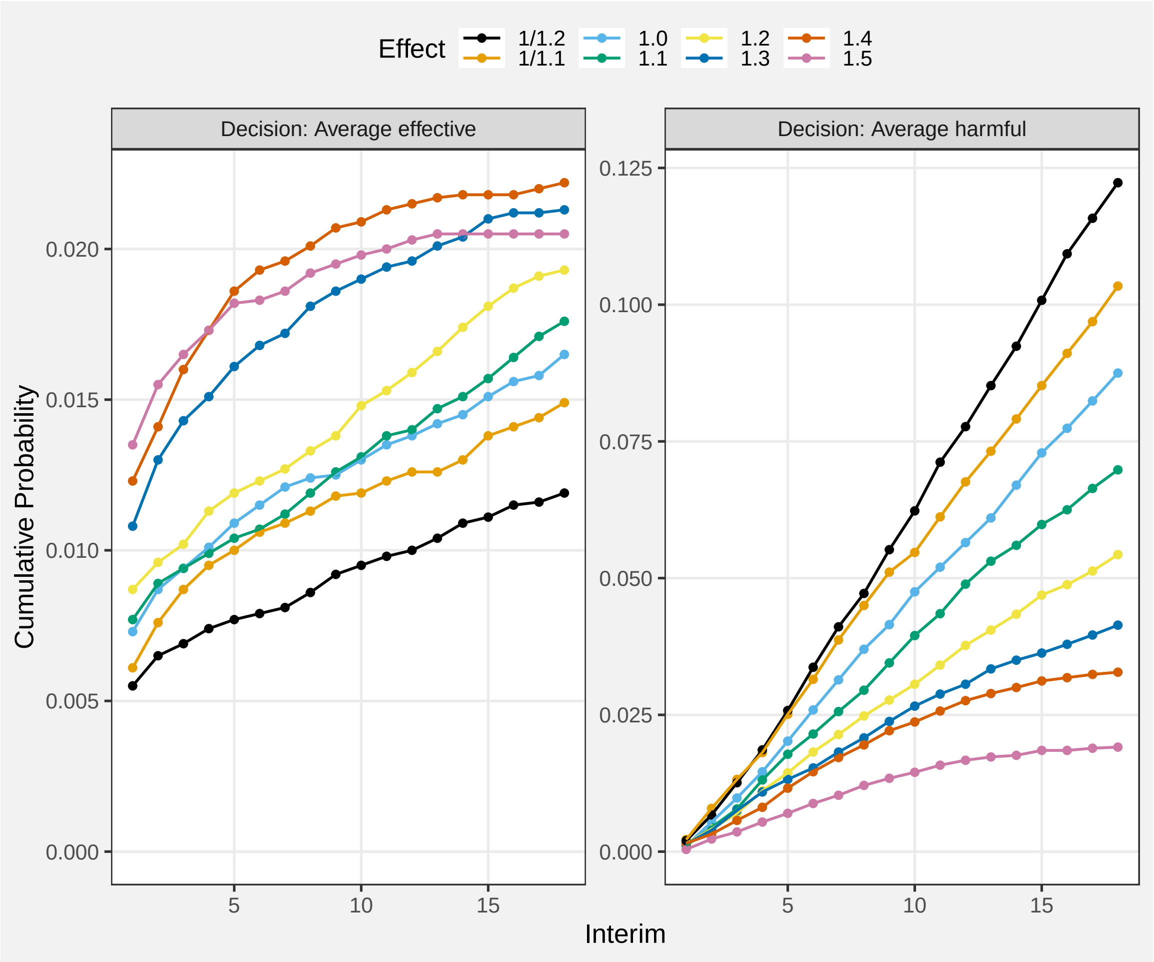

Figure 14: Decision times for average intervention effect.

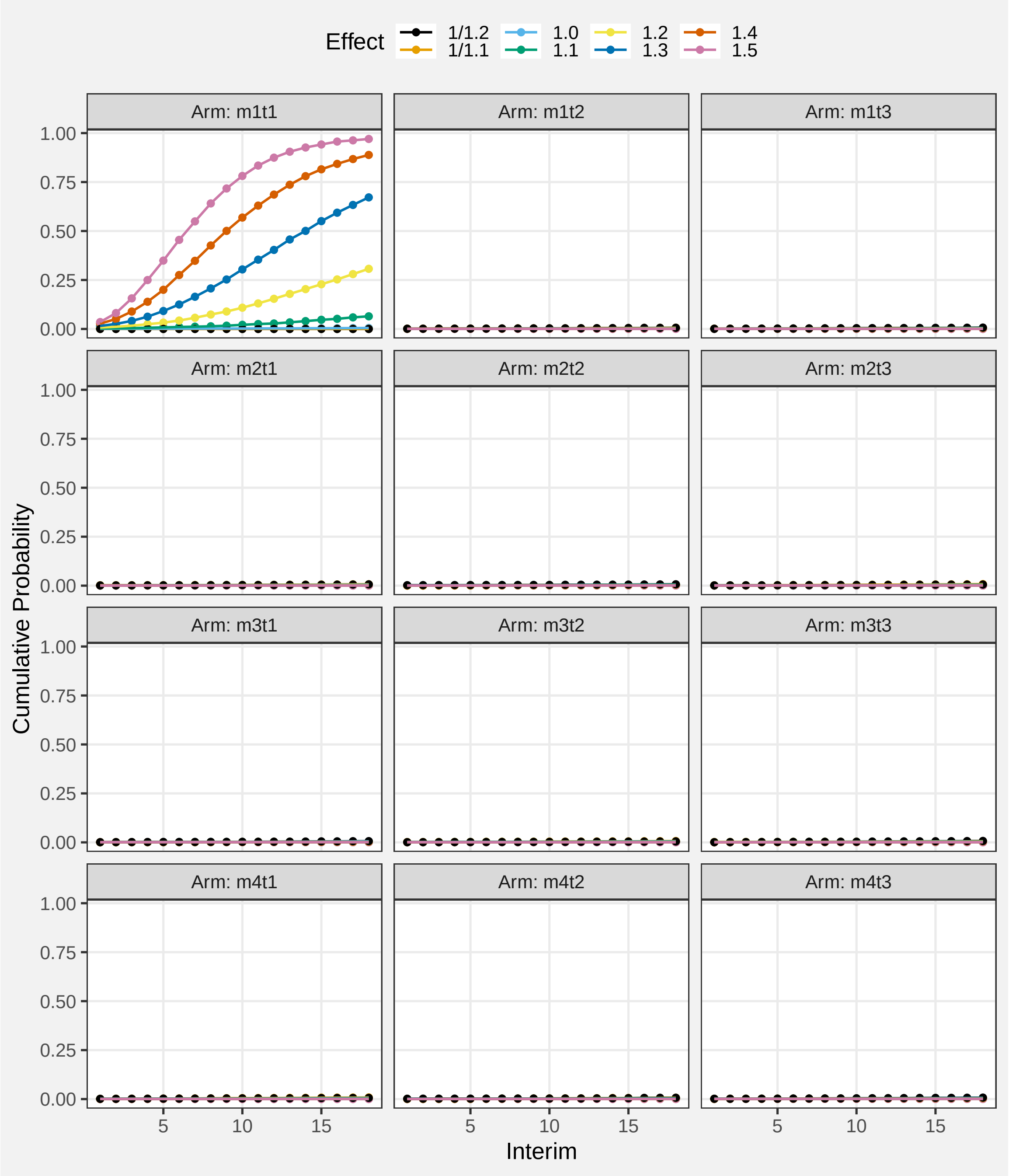

Figure 15: Decision times for superiority of each intervention.

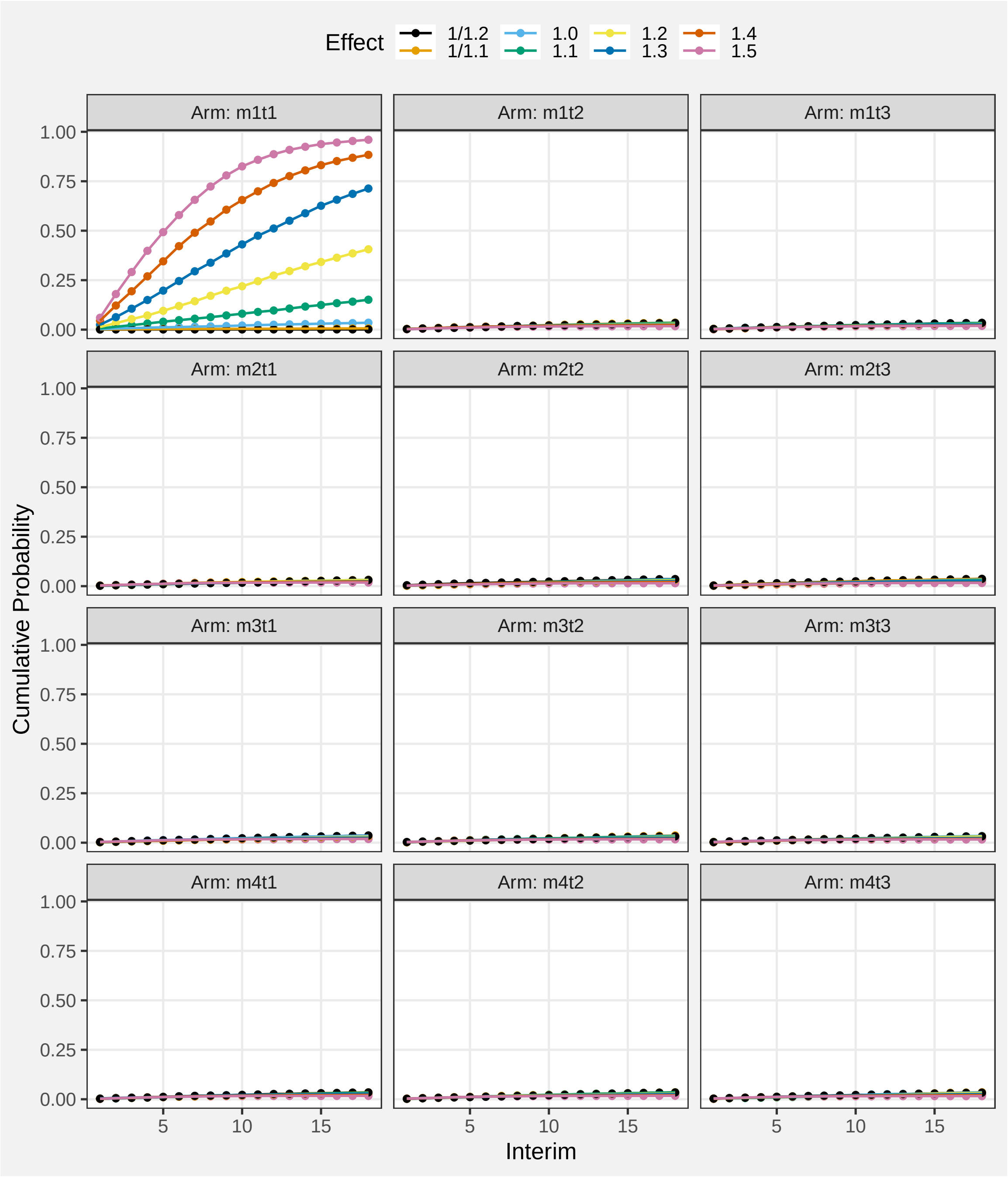

Figure 16: Decision times for effectiveness of each intervention.

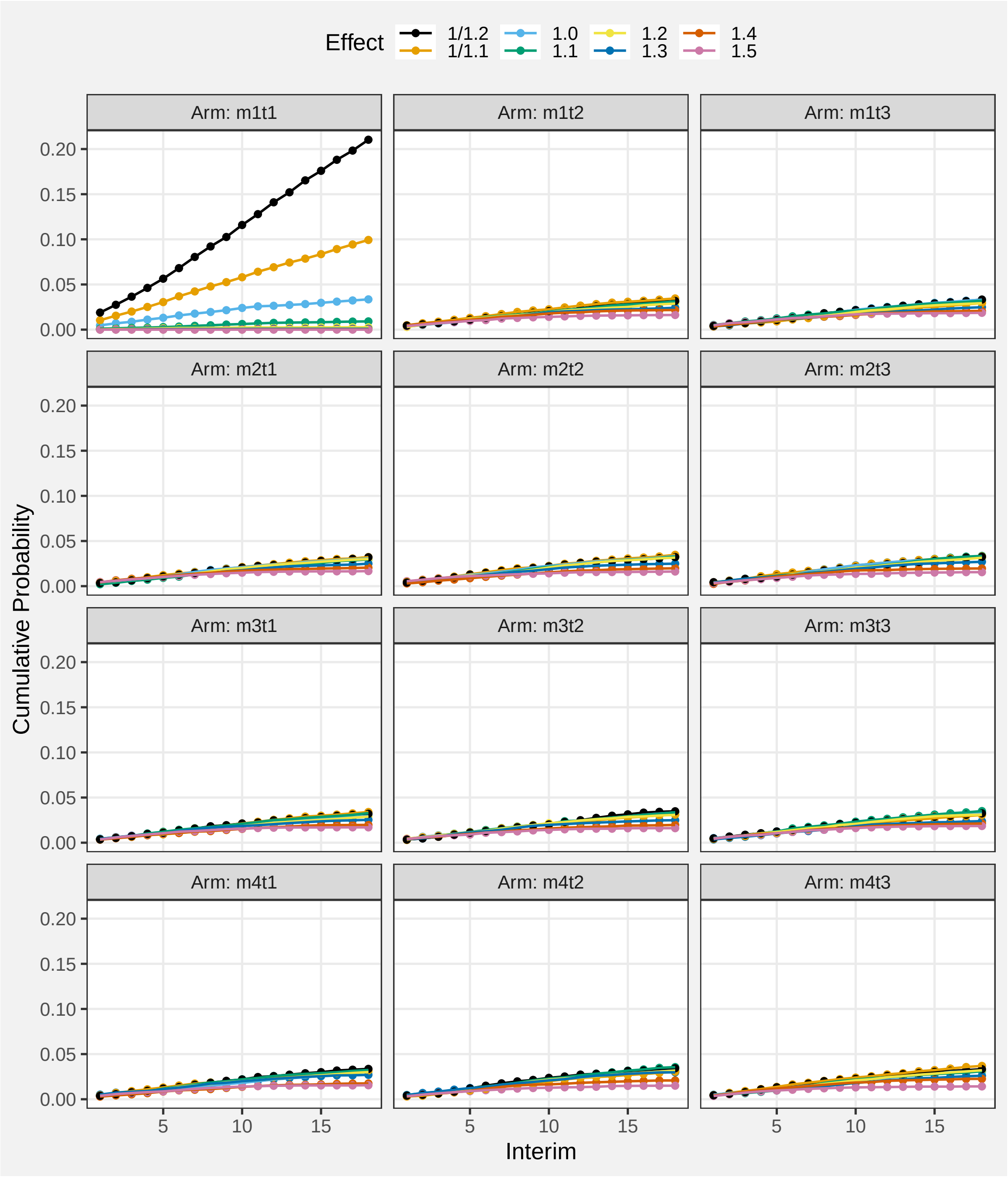

Figure 17: Decision times for harmfulness of each intervention.

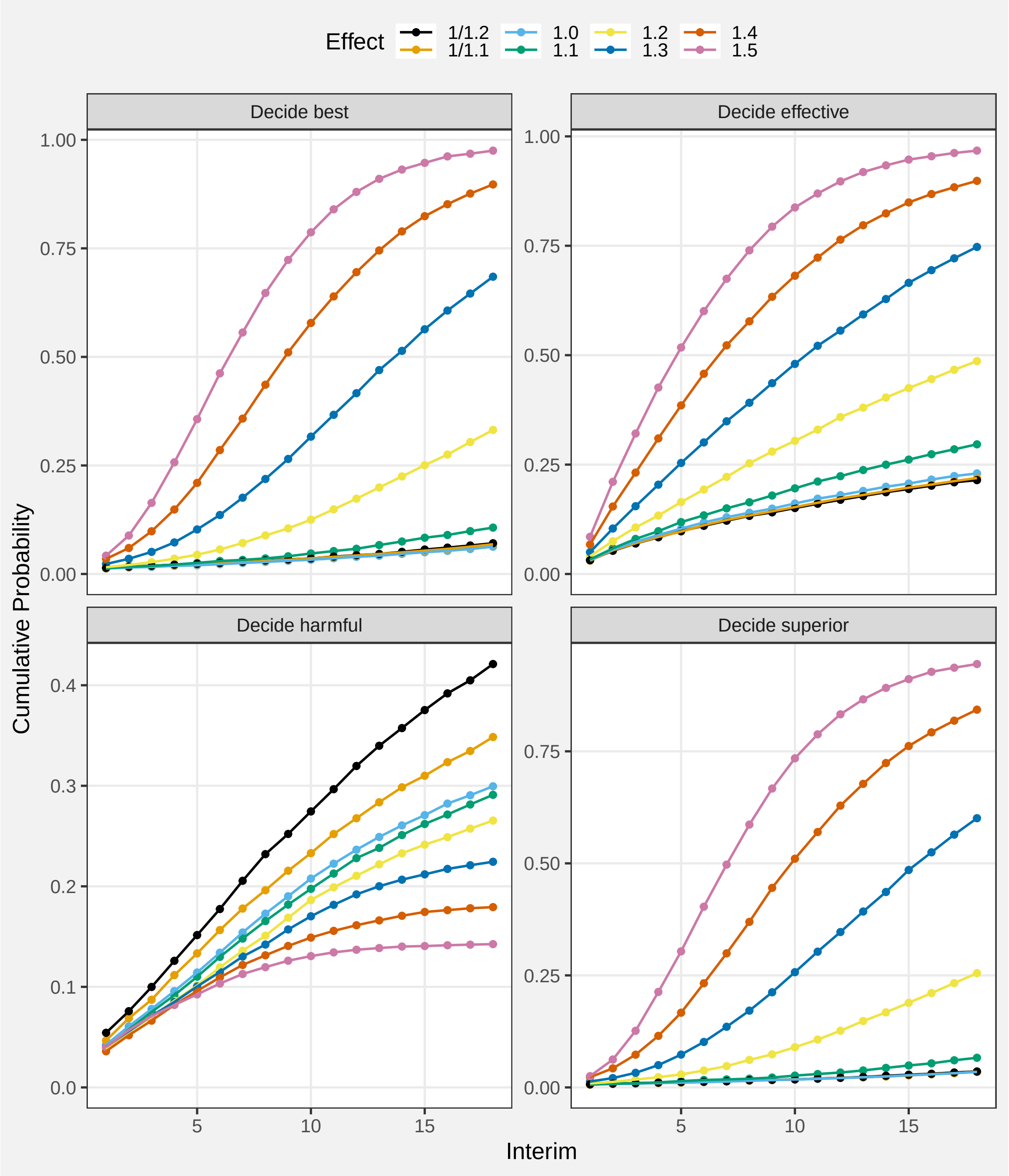

Figure 18: Earliest decision times across all intervention arms.

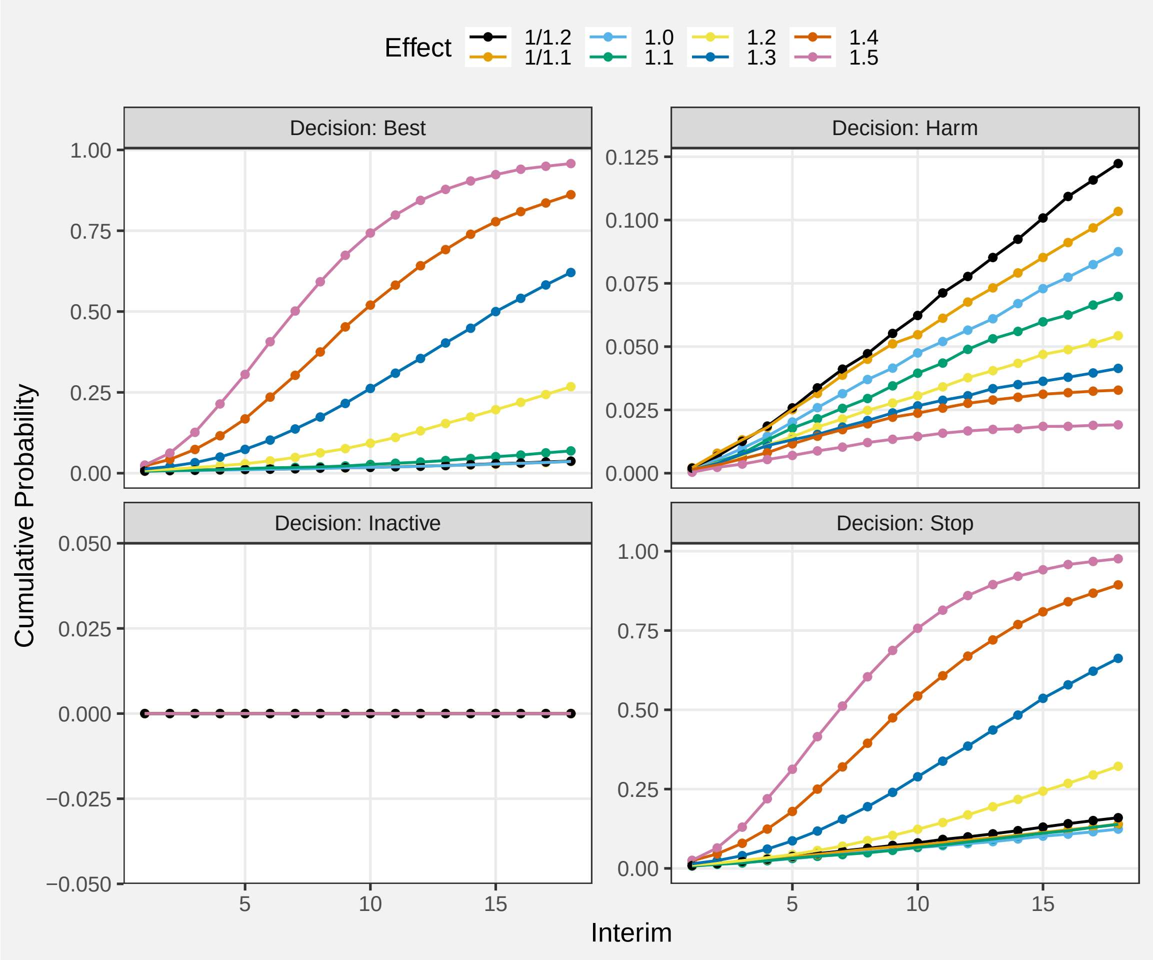

Figure 19: Stopping times and reason for stopping.

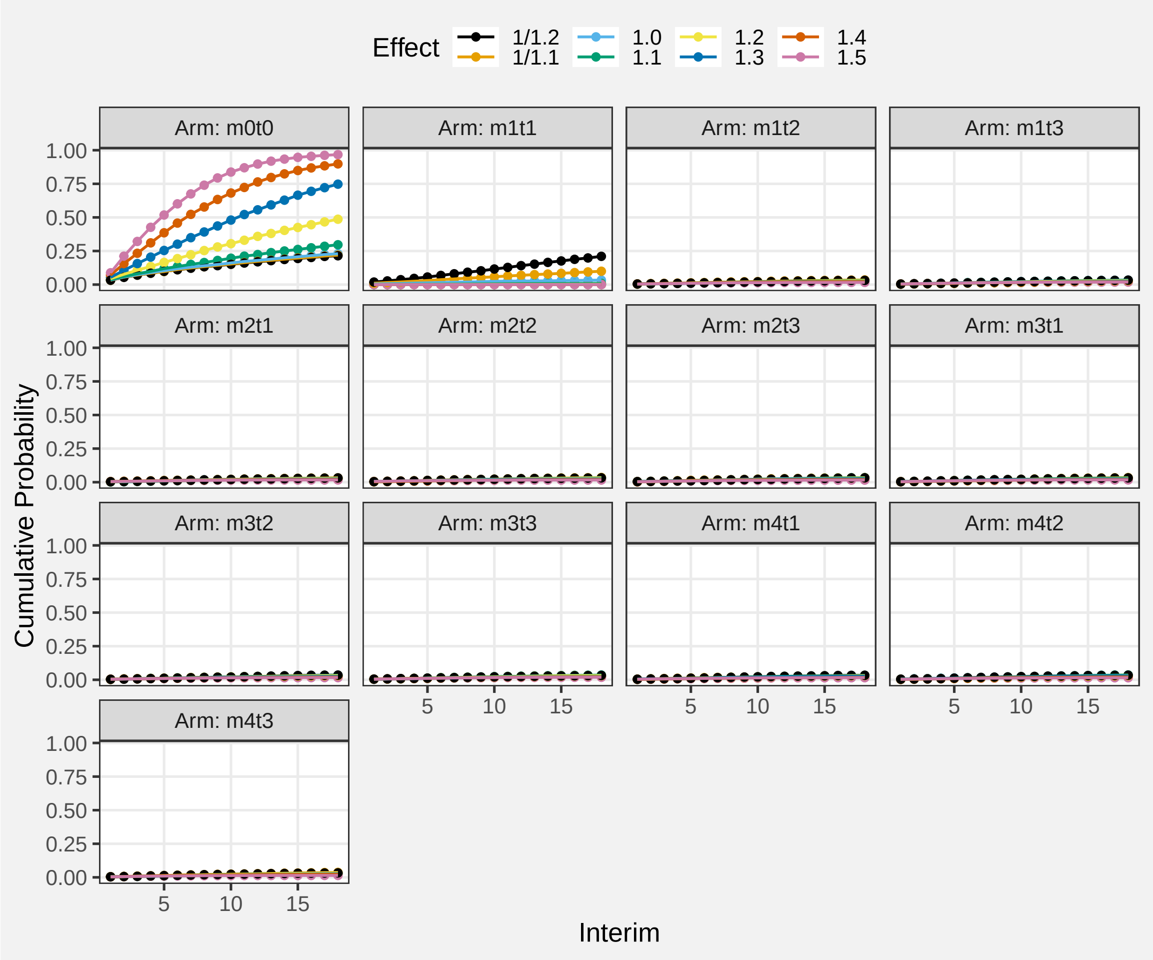

Figure 20: Decision times for dropping each intervention arm.

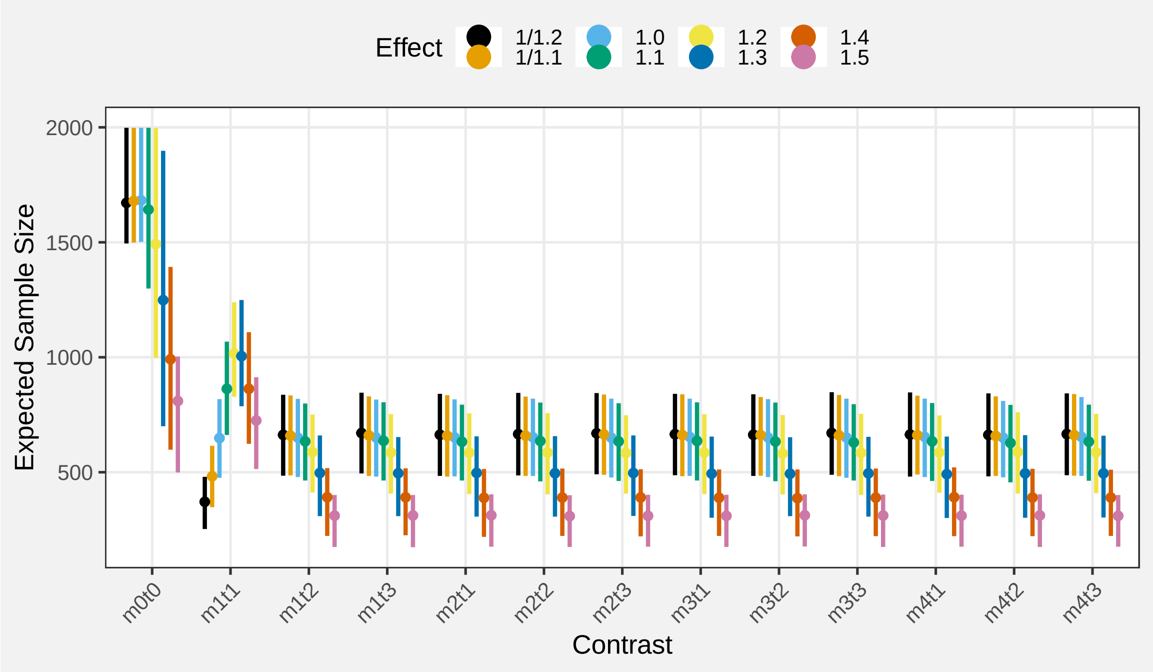

Figure 21: Expected sample size by arm.

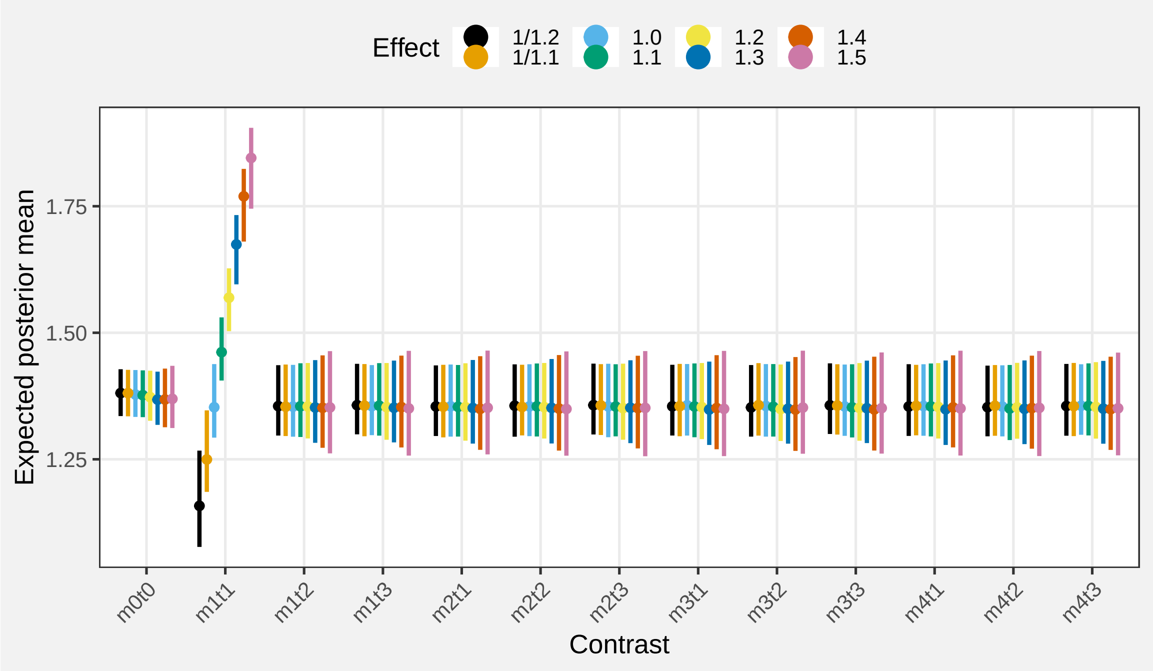

Figure 22: Expected posterior mean by arm.

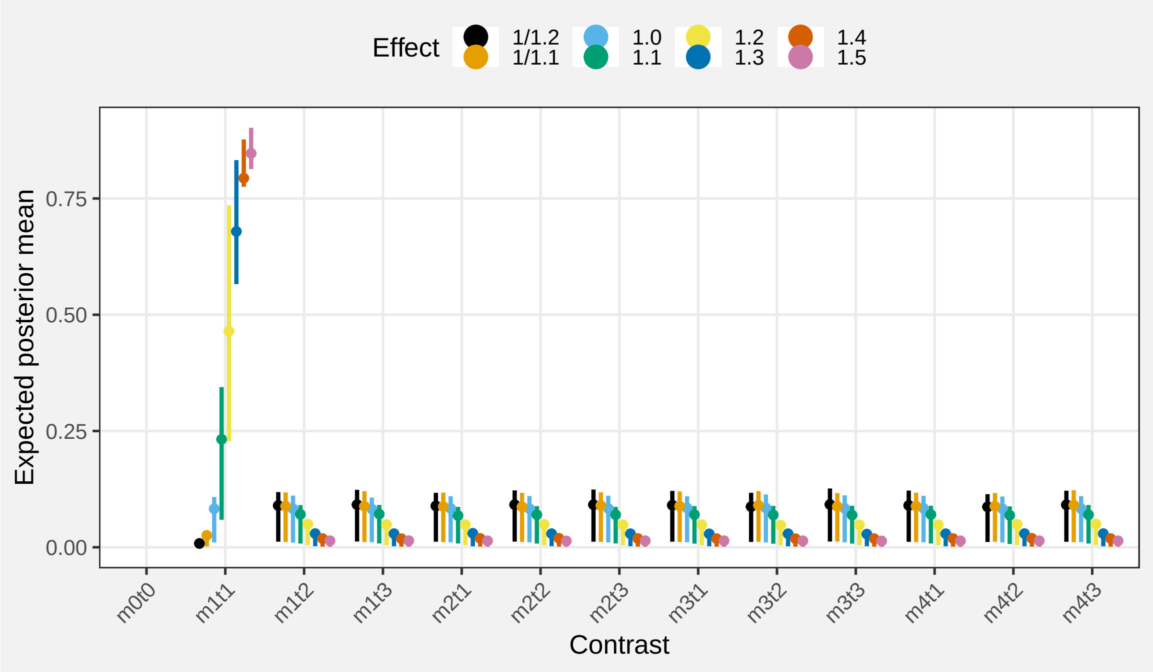

Figure 23: Expected posterior probability of superiority by arm.

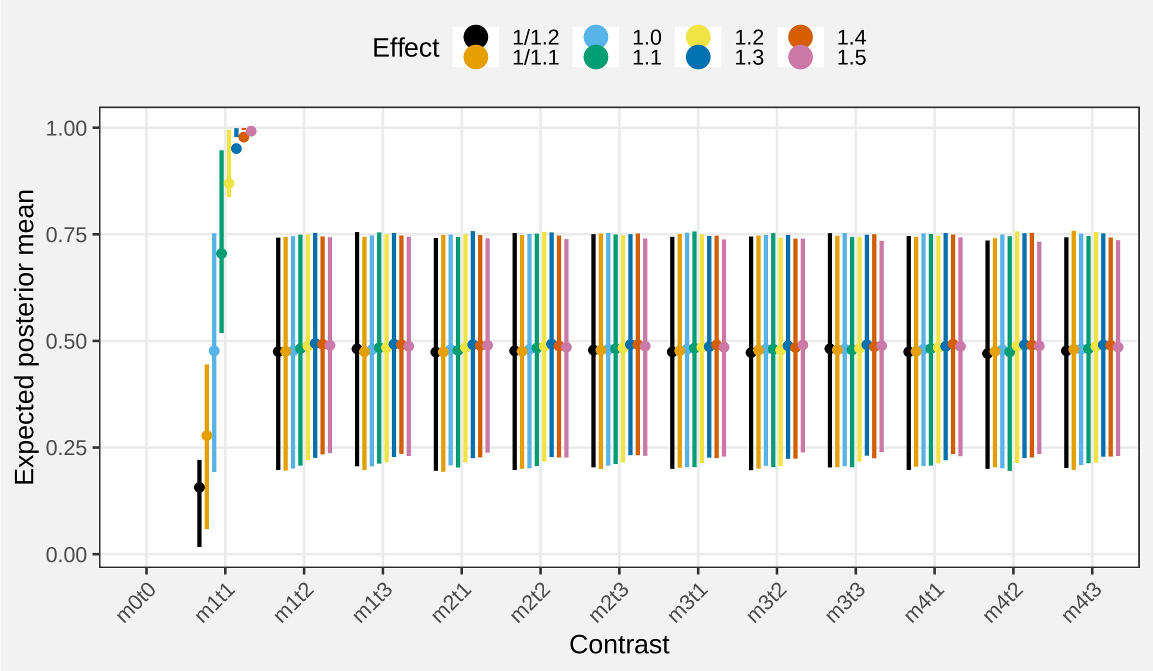

Figure 24: Expected posterior probability of effectiveness by arm.

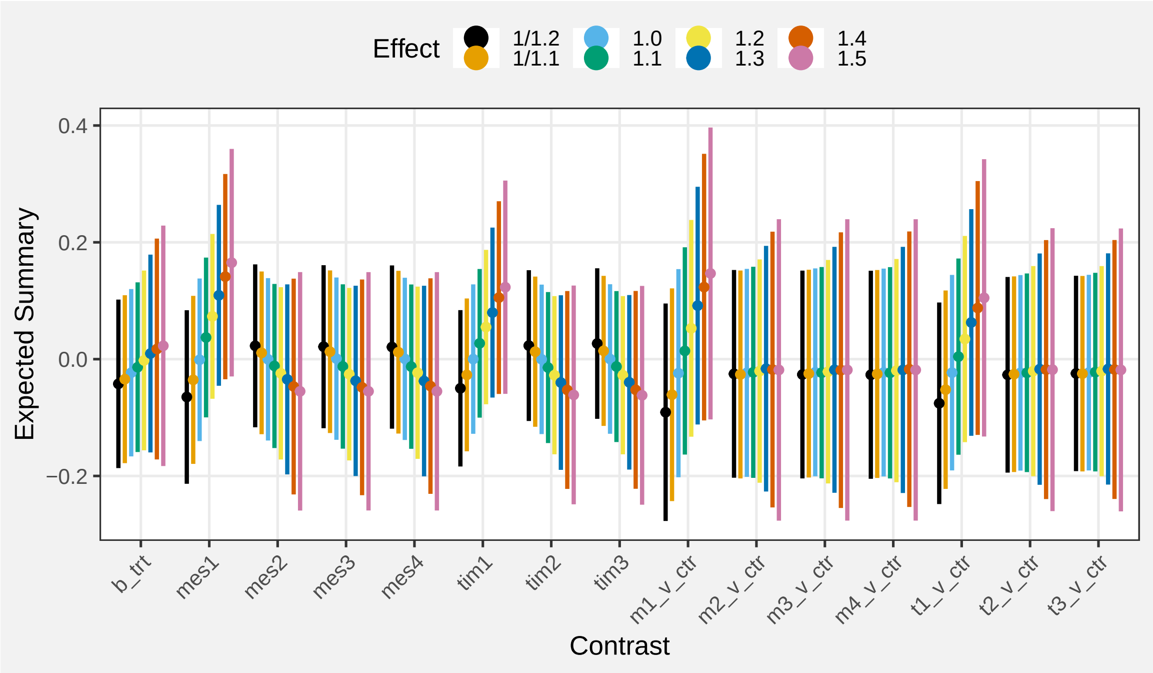

Figure 25: Expected posterior summary of contrasts.

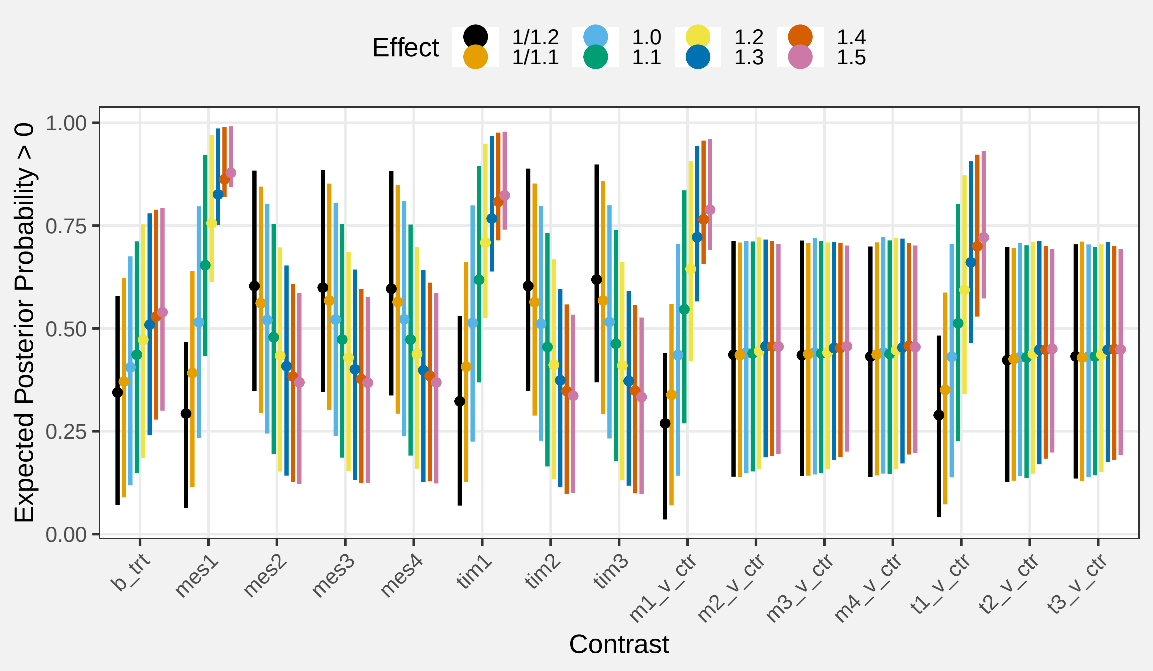

Figure 26: Expected posterior probability of contrast effect.

7.2.3 One timing effective

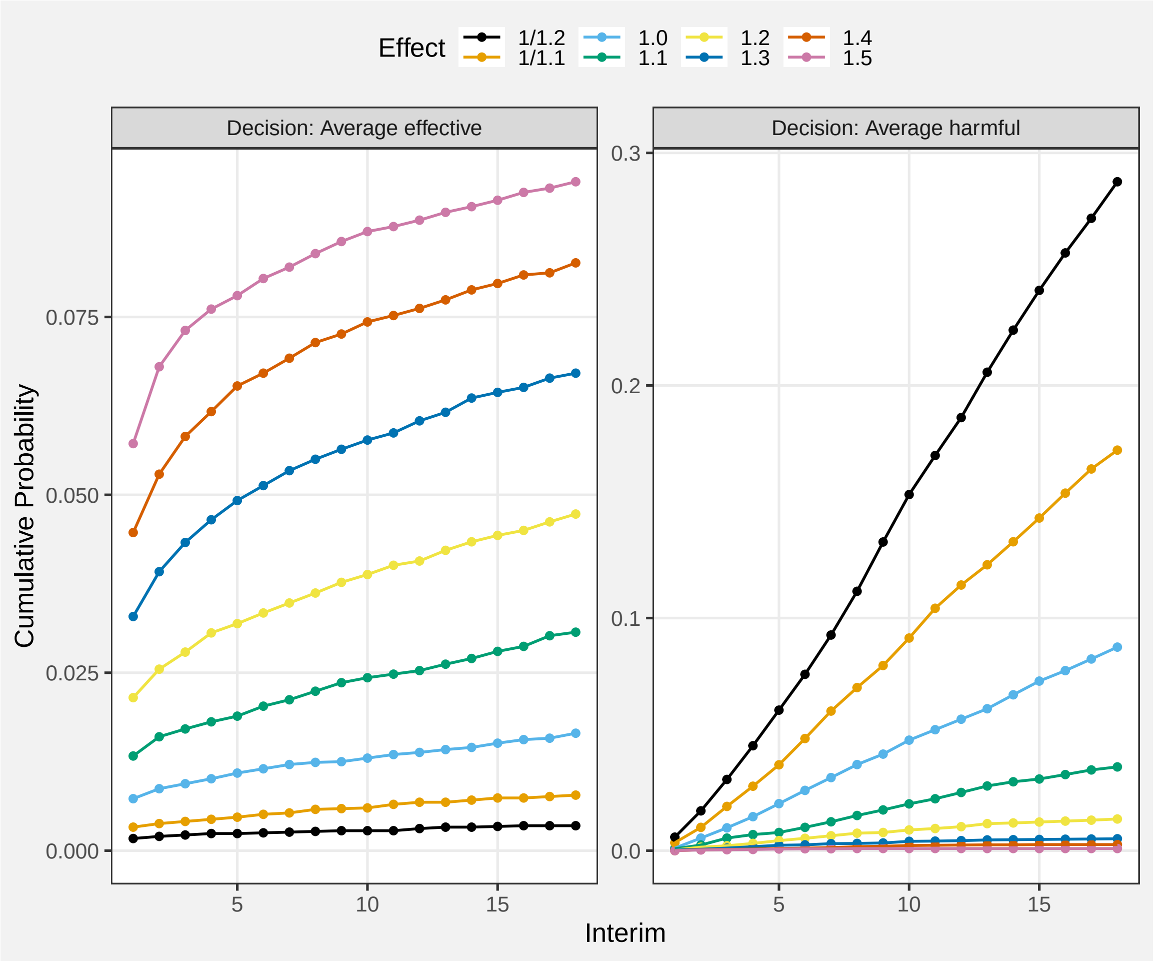

Figure 27: Decision times for average intervention effect.

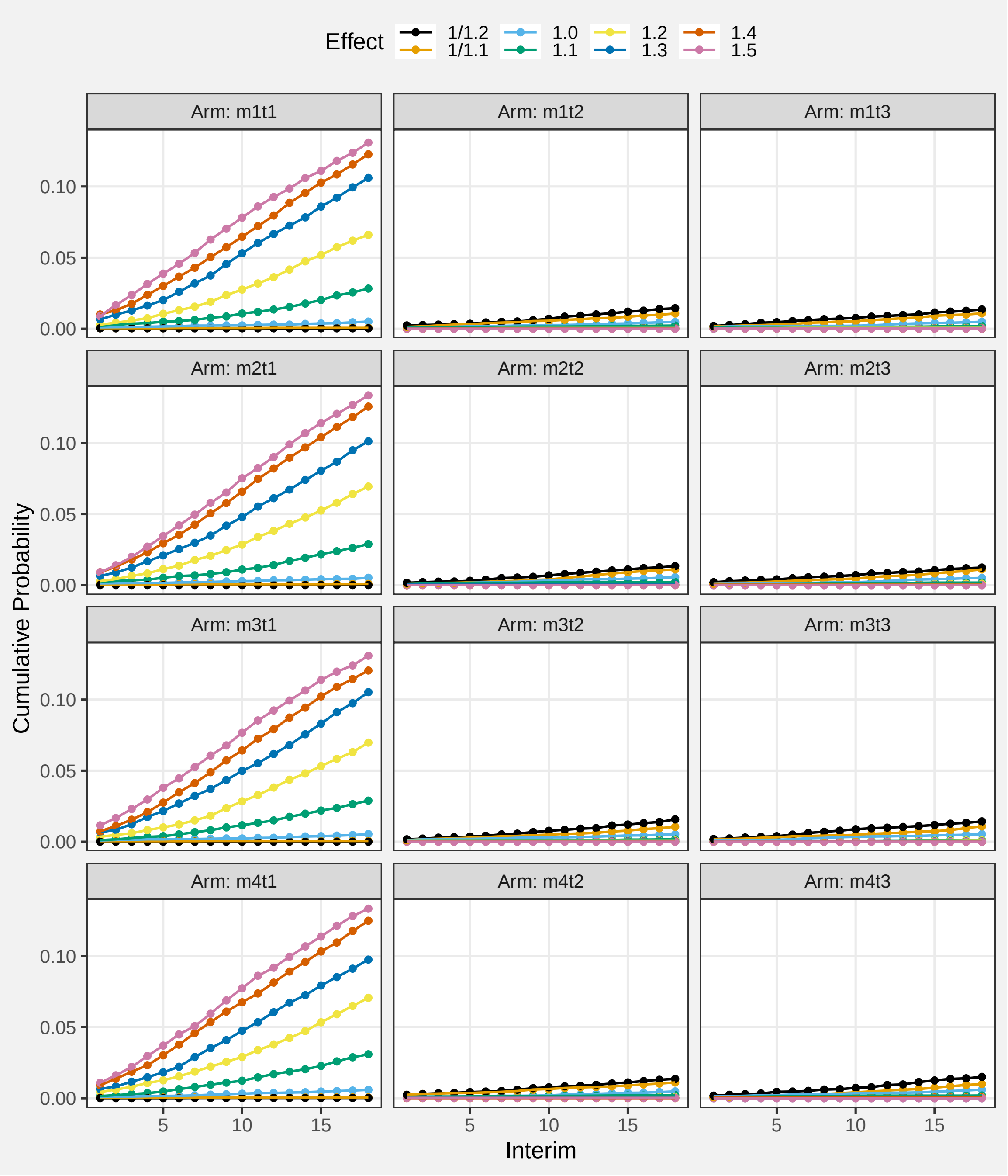

Figure 28: Decision times for superiority of each intervention.

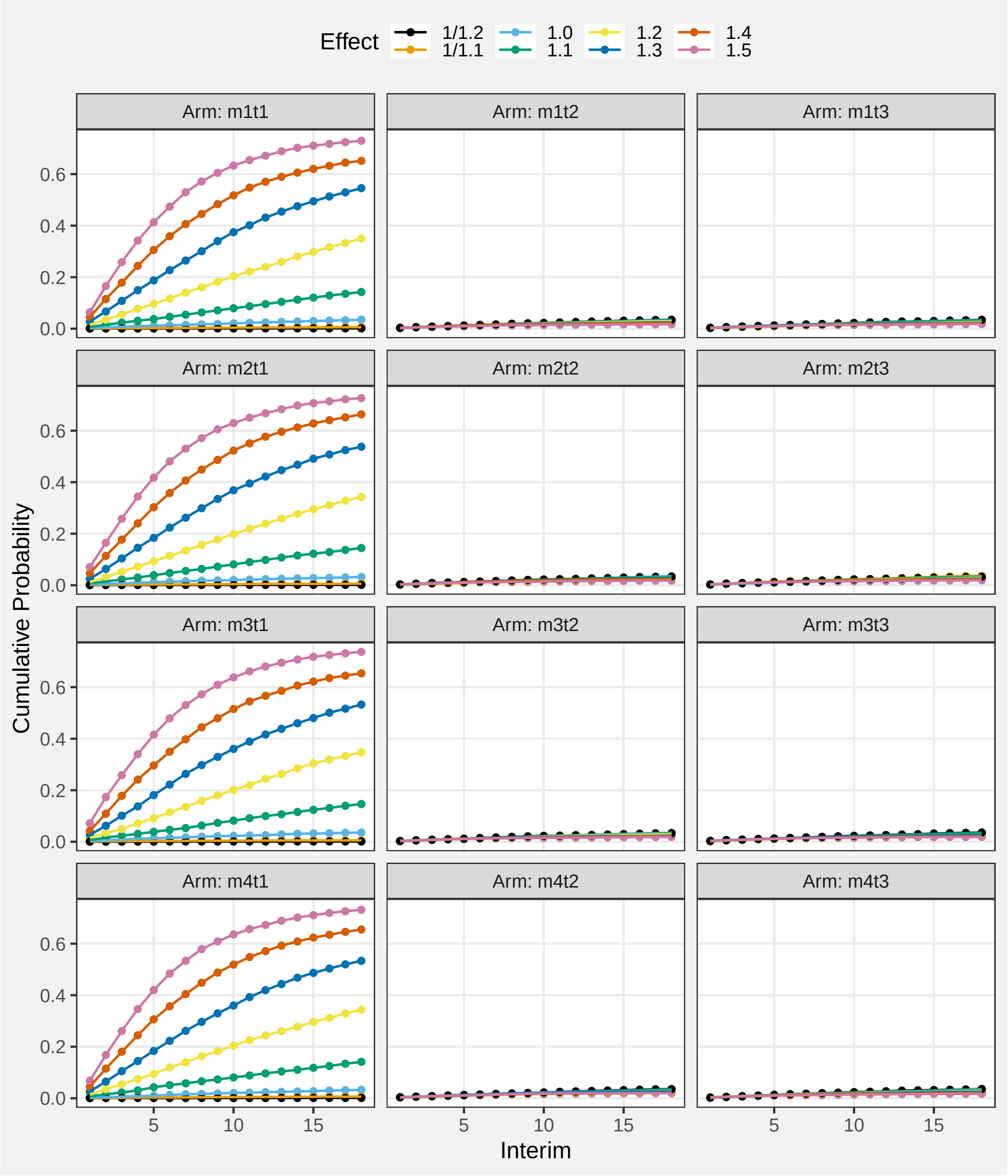

Figure 29: Decision times for effectiveness of each intervention.

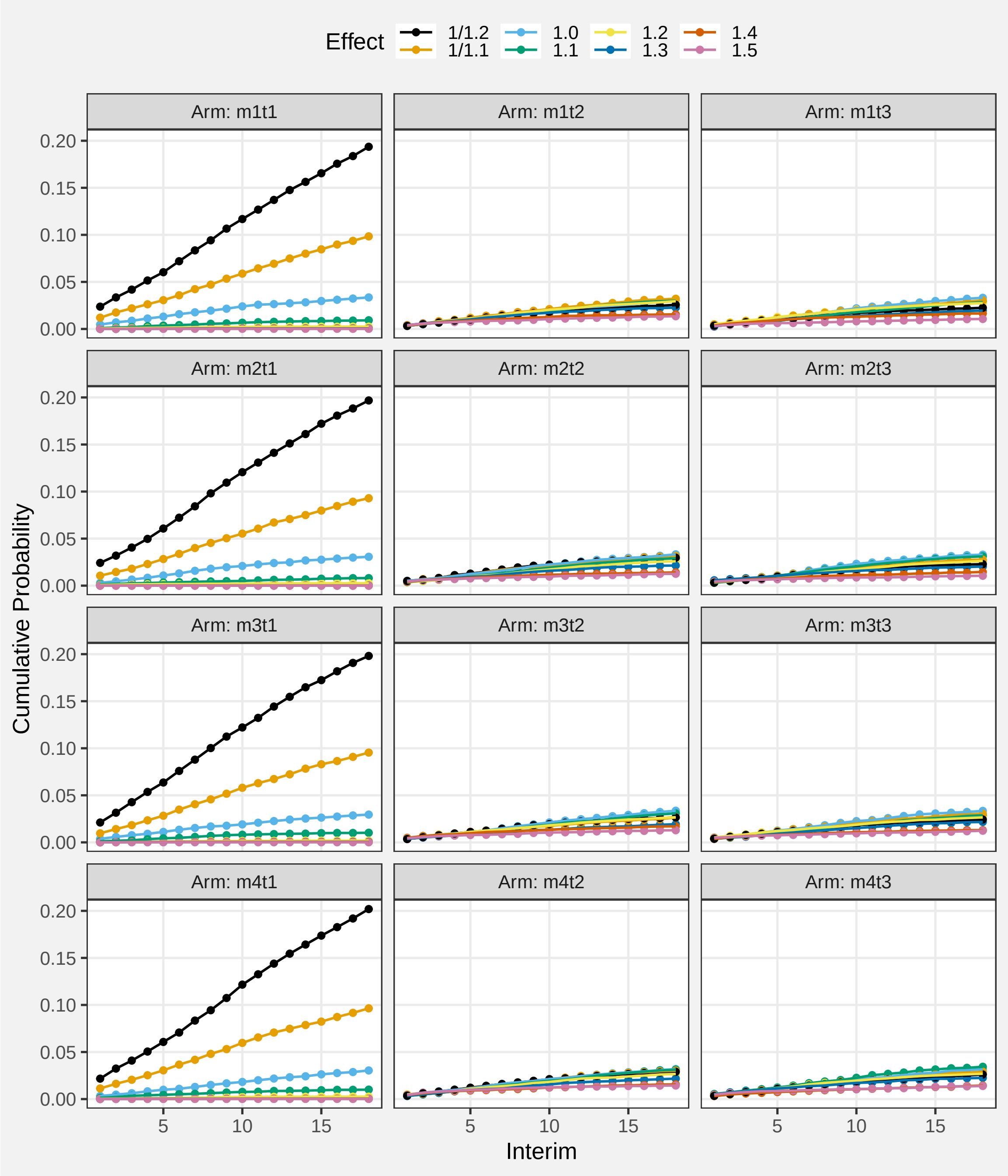

Figure 30: Decision times for harmfulness of each intervention.

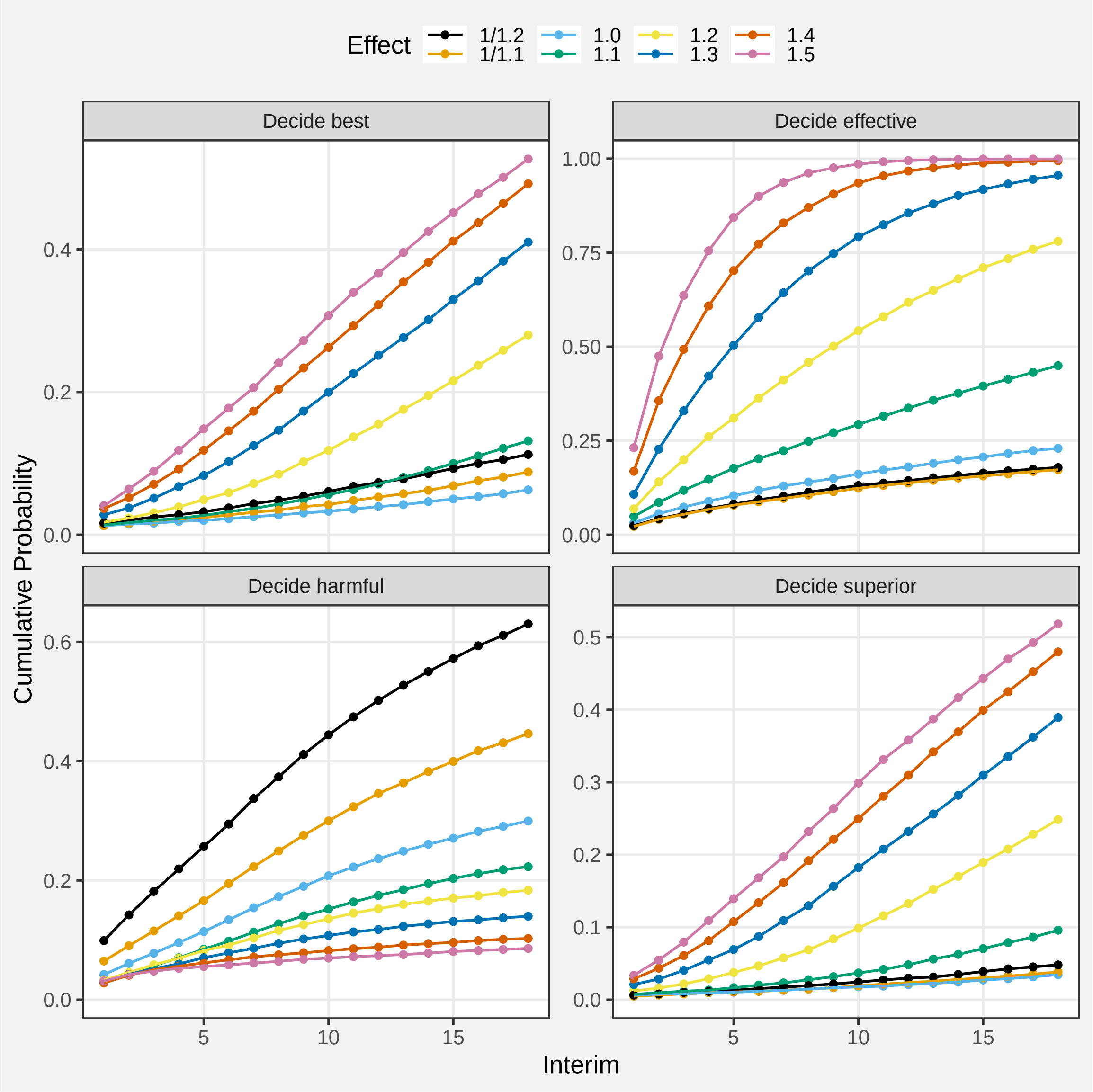

Figure 31: Earliest decision times across all intervention arms.

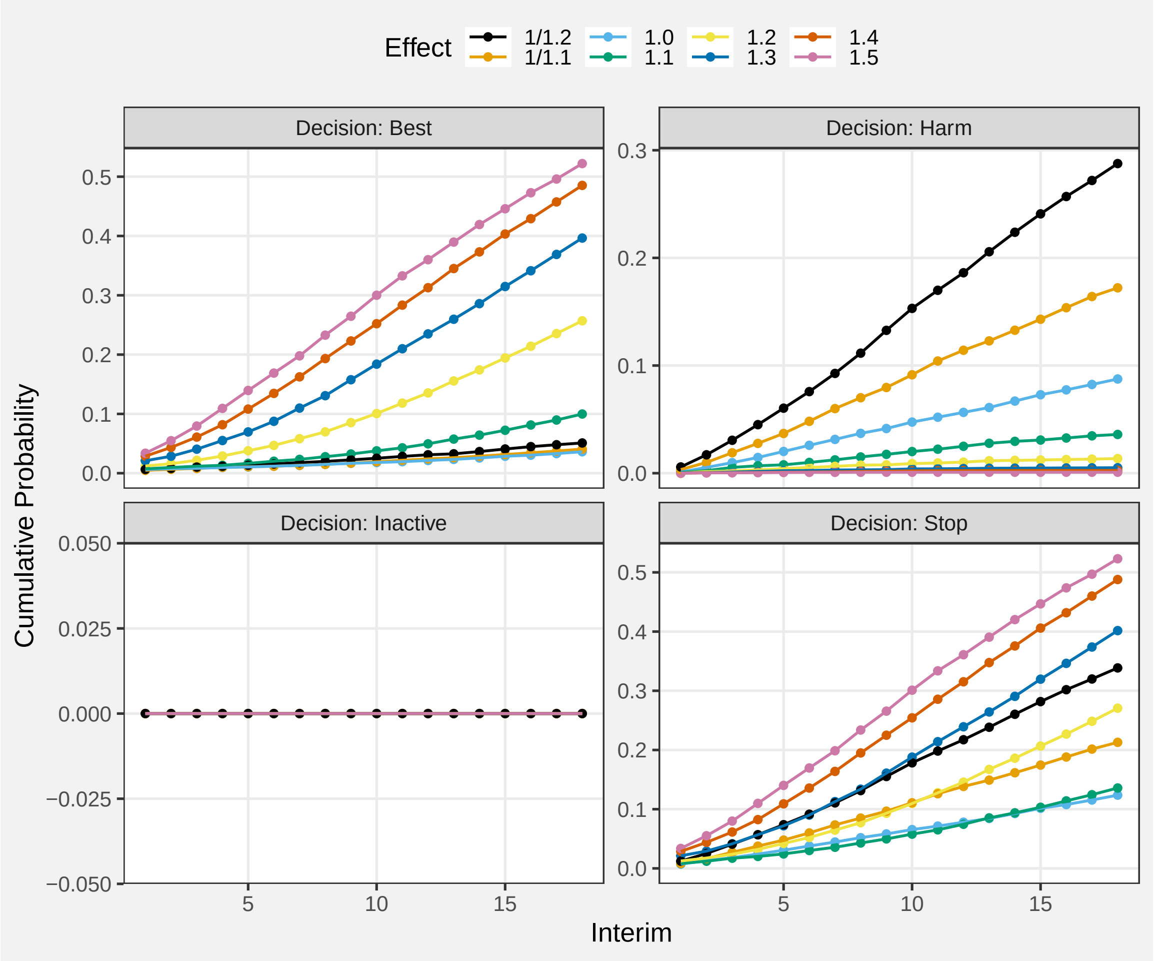

Figure 32: Stopping times and reason for stopping.

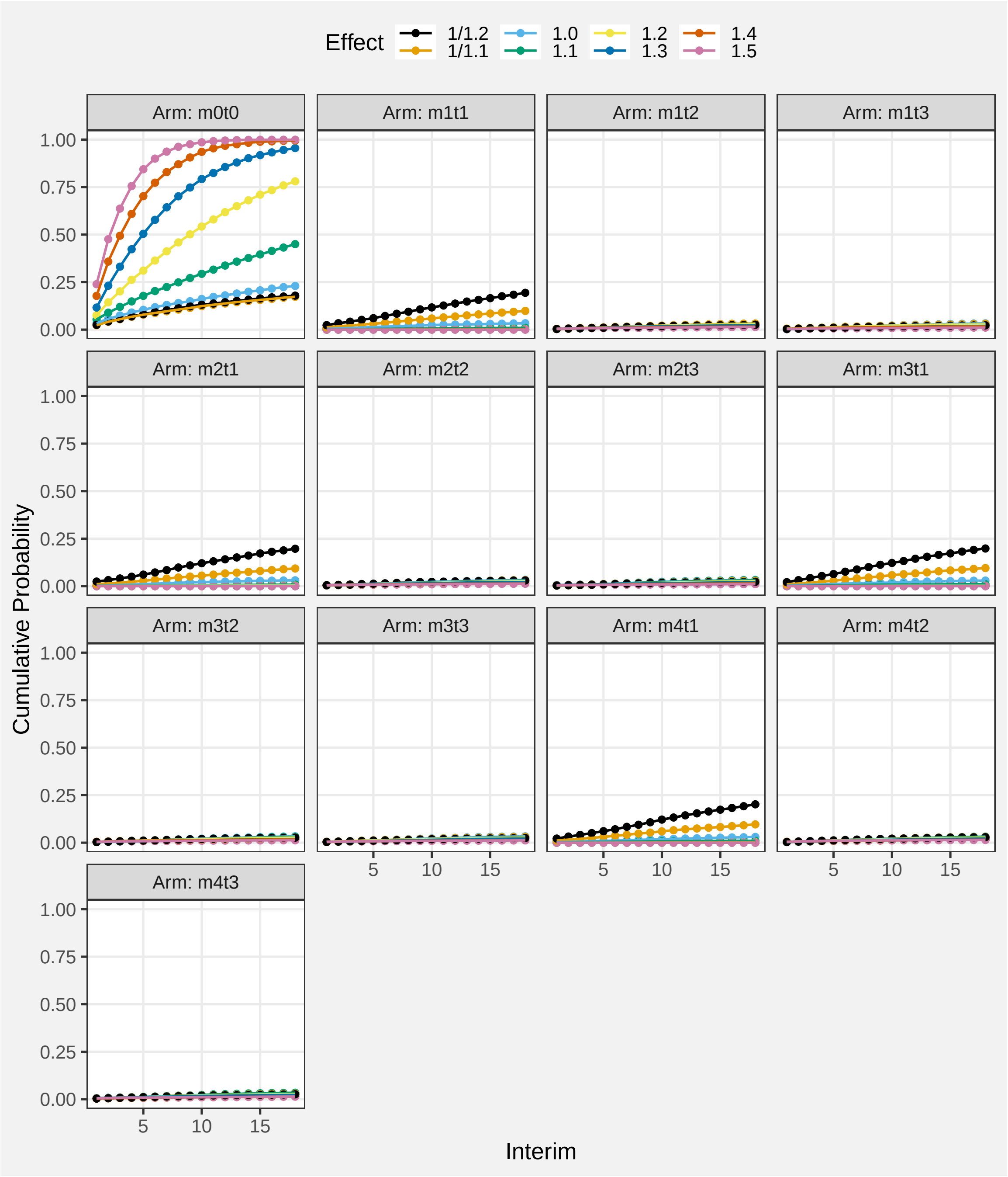

Figure 33: Decision times for dropping each intervention arm.

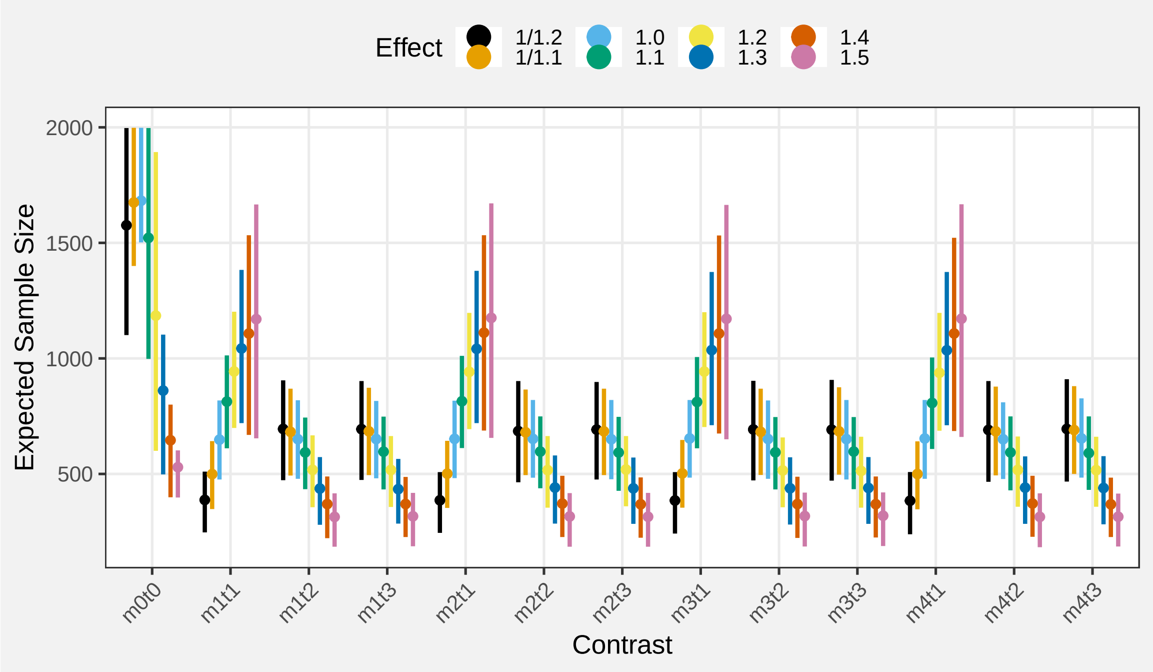

Figure 34: Expected sample size by arm.

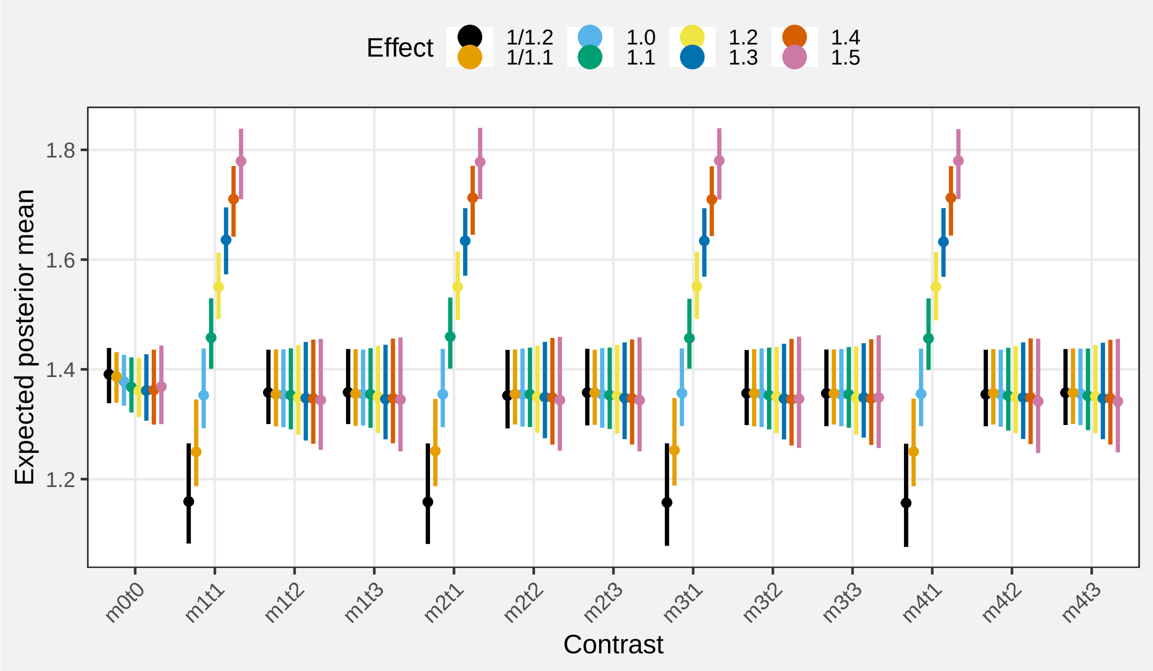

Figure 35: Expected posterior mean by arm.

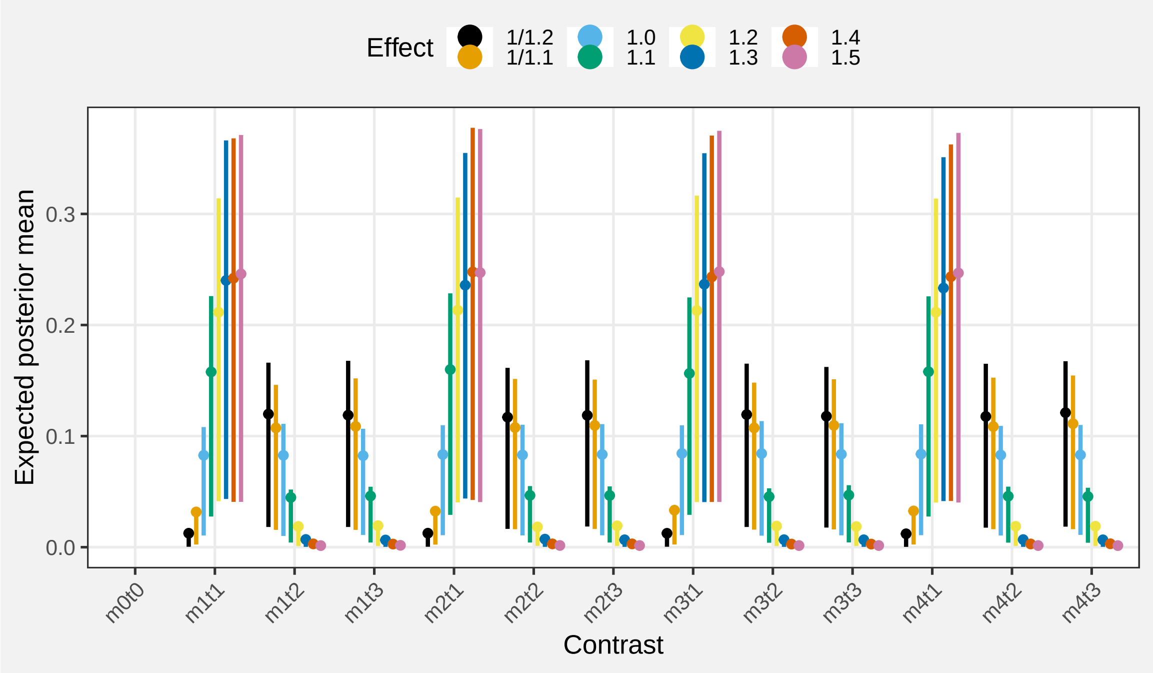

Figure 36: Expected posterior probability of superiority by arm.

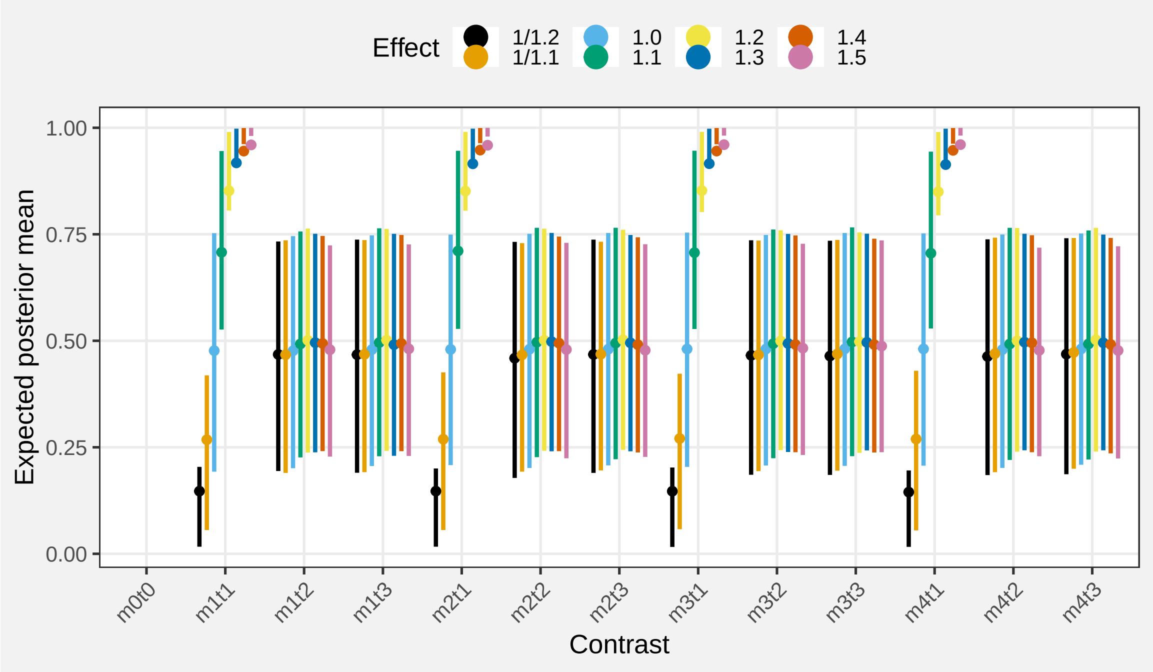

Figure 37: Expected posterior probability of effectiveness by arm.

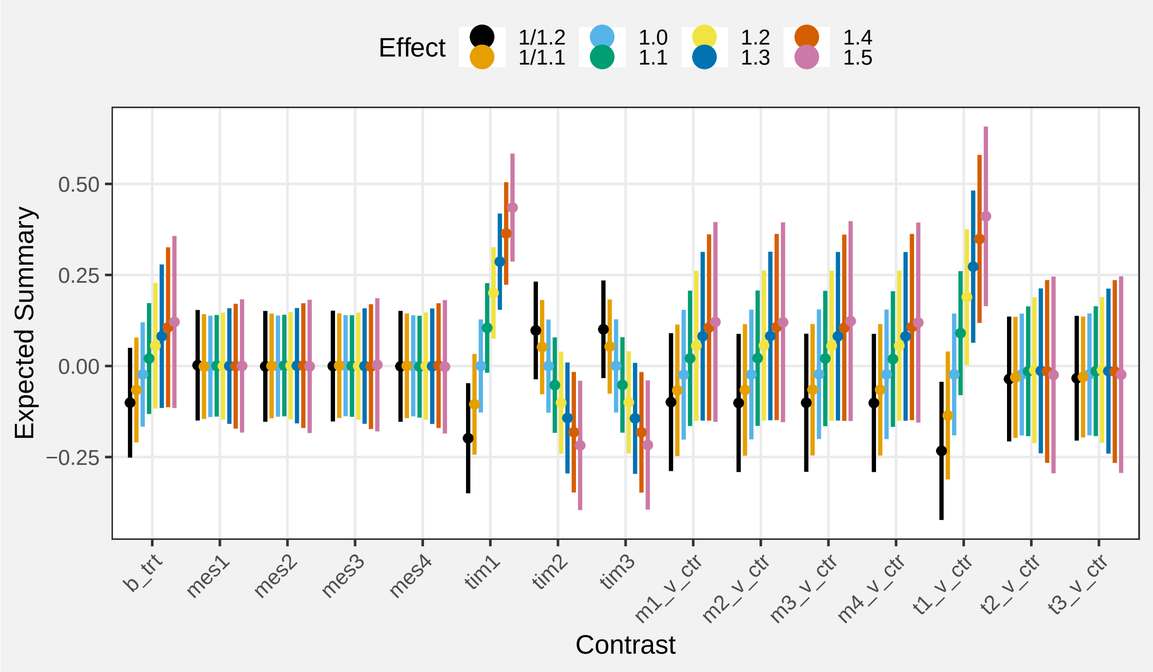

Figure 38: Expected posterior summary of contrasts.

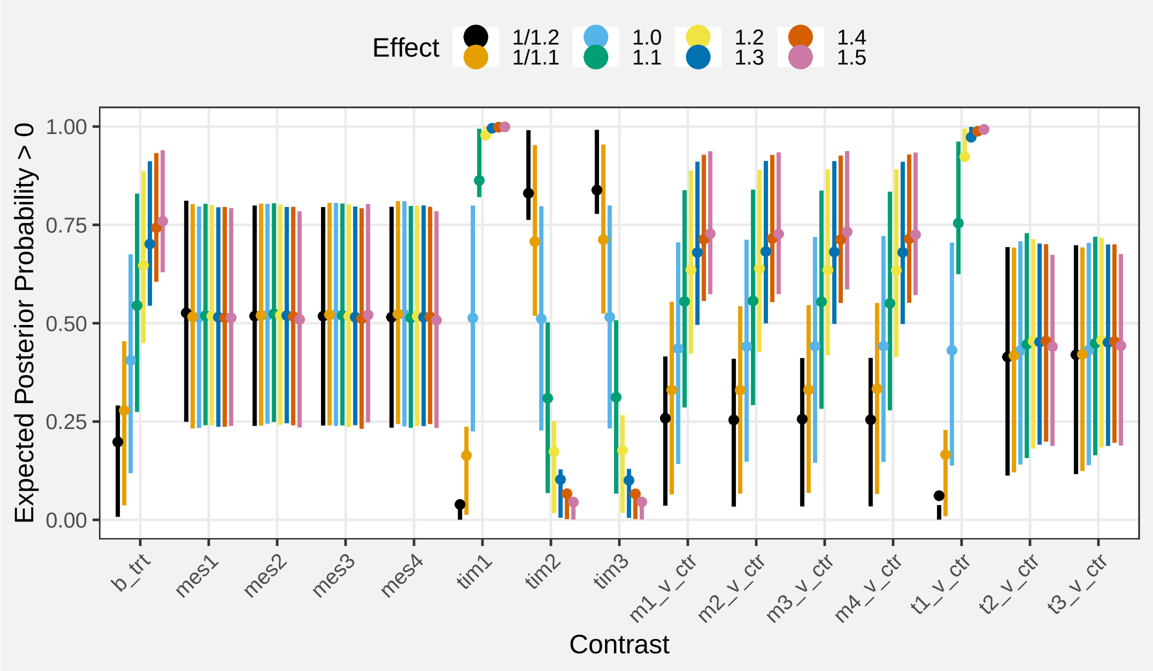

Figure 39: Expected posterior probability of contrast effect.

7.2.4 One framing effective

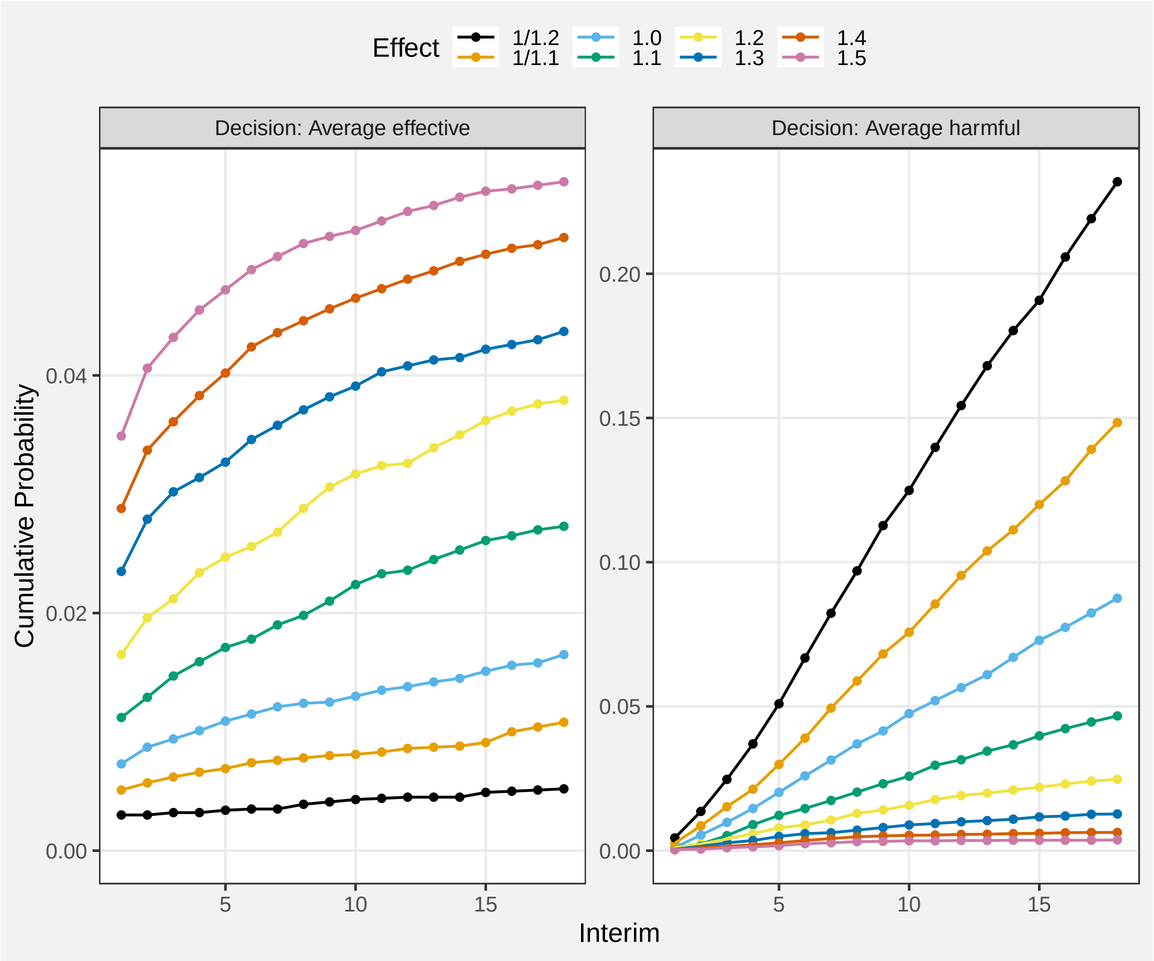

Figure 40: Decision times for average intervention effect.

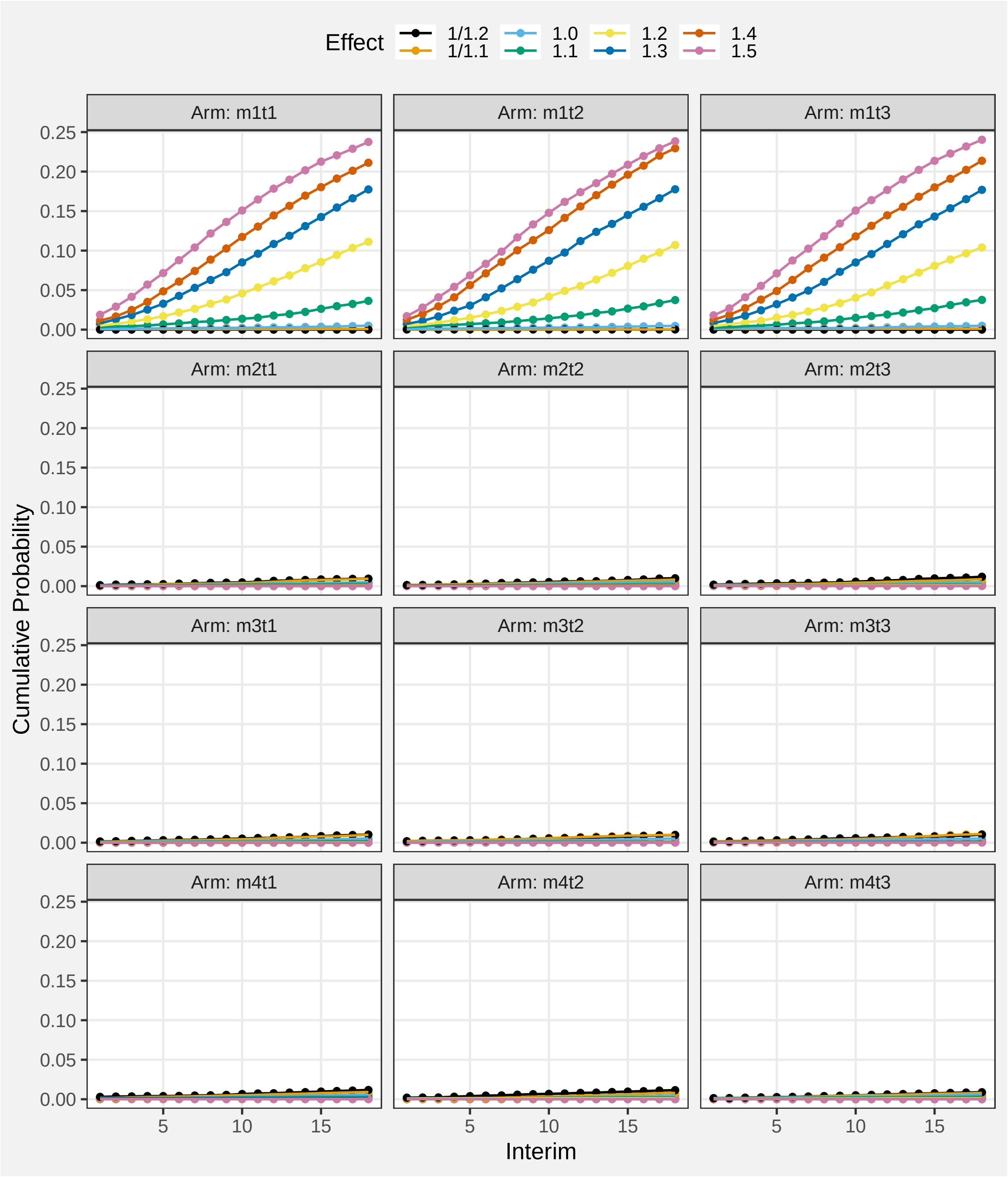

Figure 41: Decision times for superiority of each intervention.

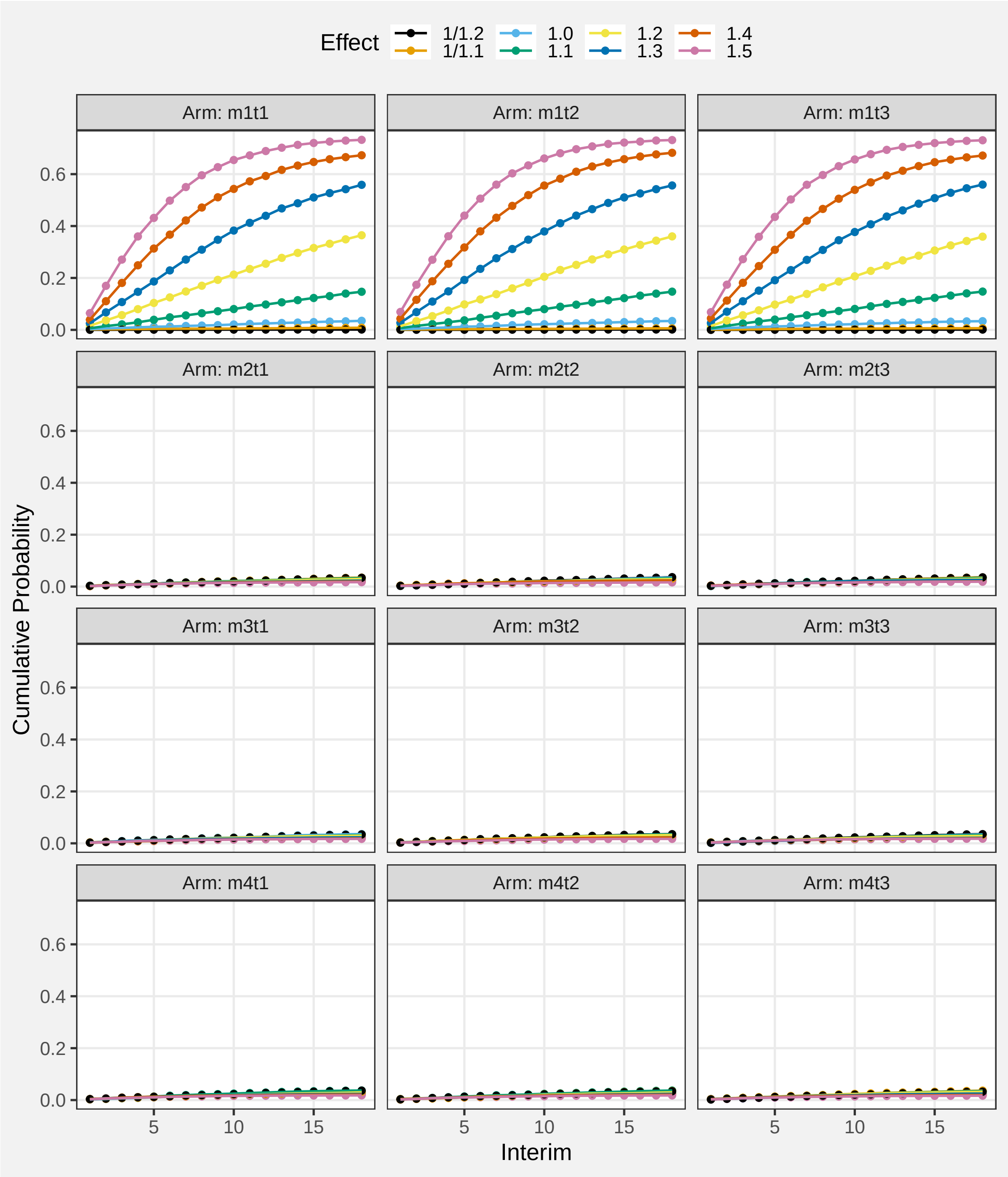

Figure 42: Decision times for effectiveness of each intervention.

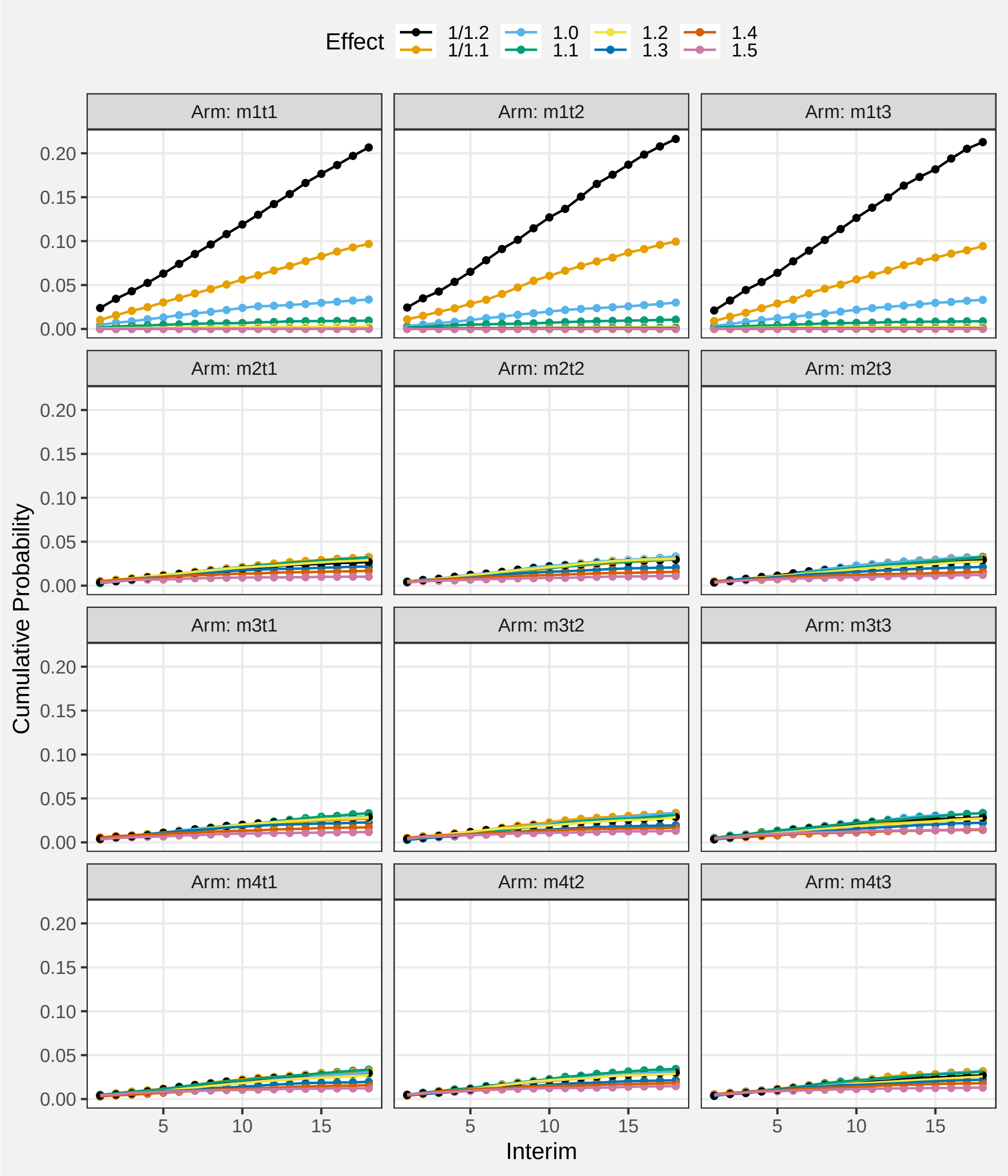

Figure 43: Decision times for harmfulness of each intervention.

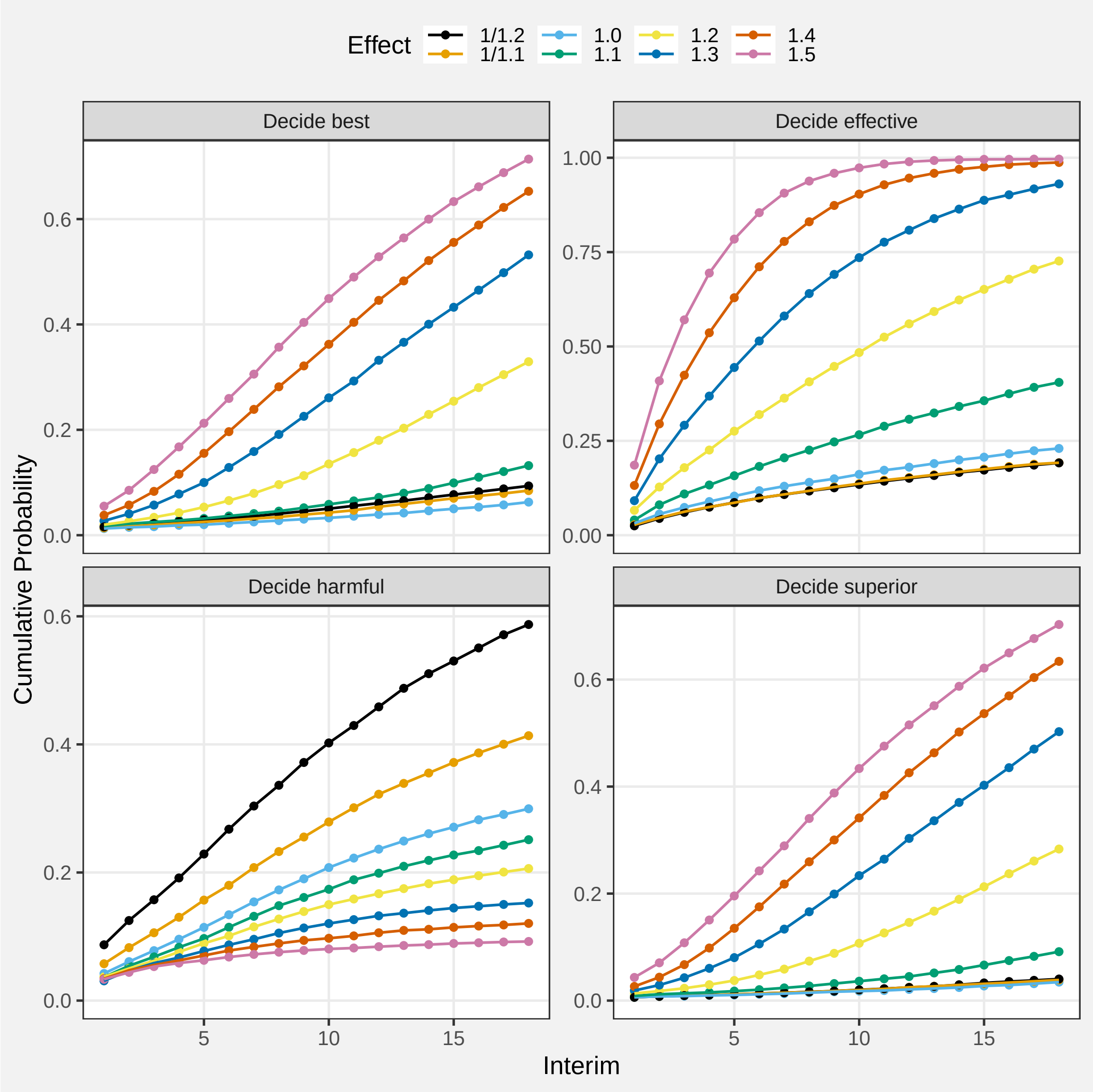

Figure 44: Earliest decision times across all intervention arms.

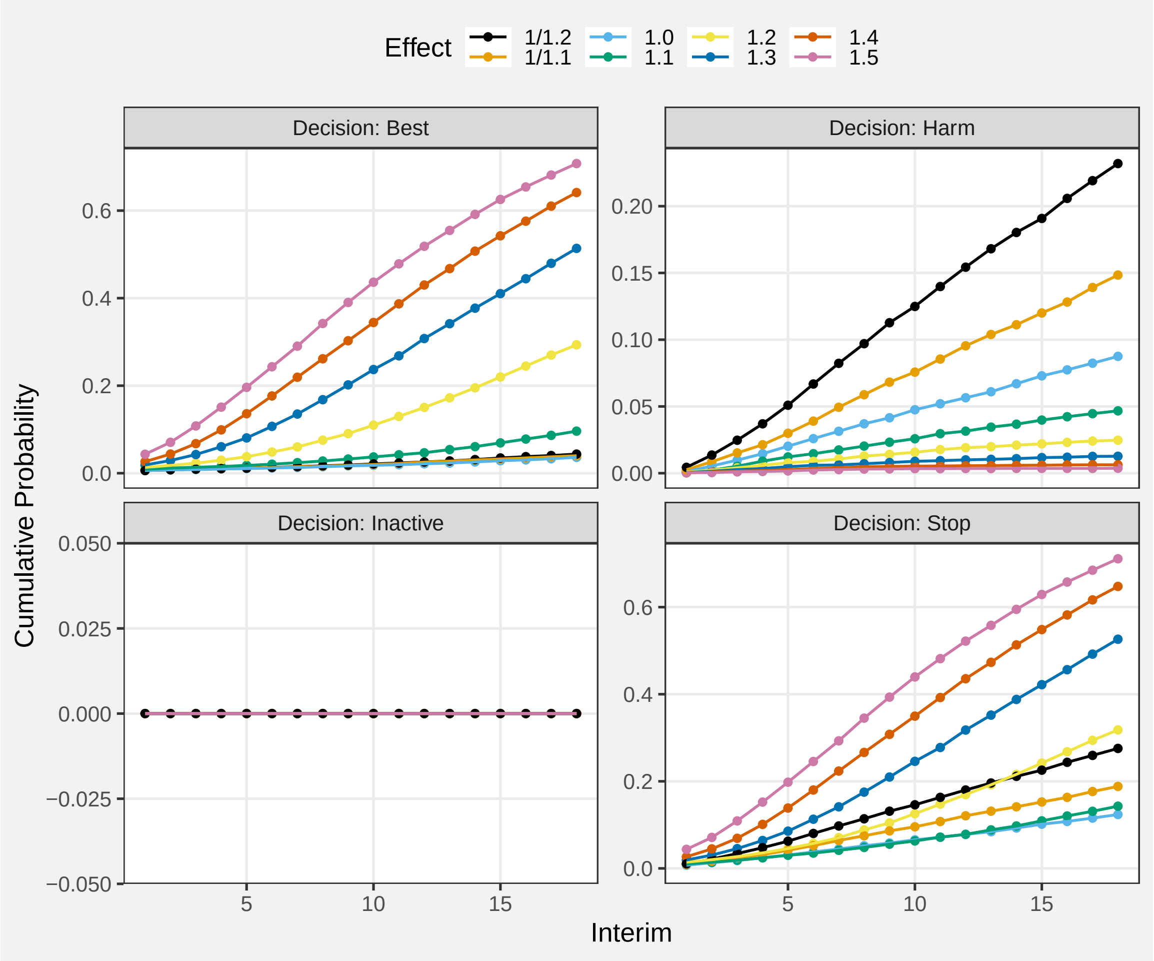

Figure 45: Stopping times and reason for stopping.

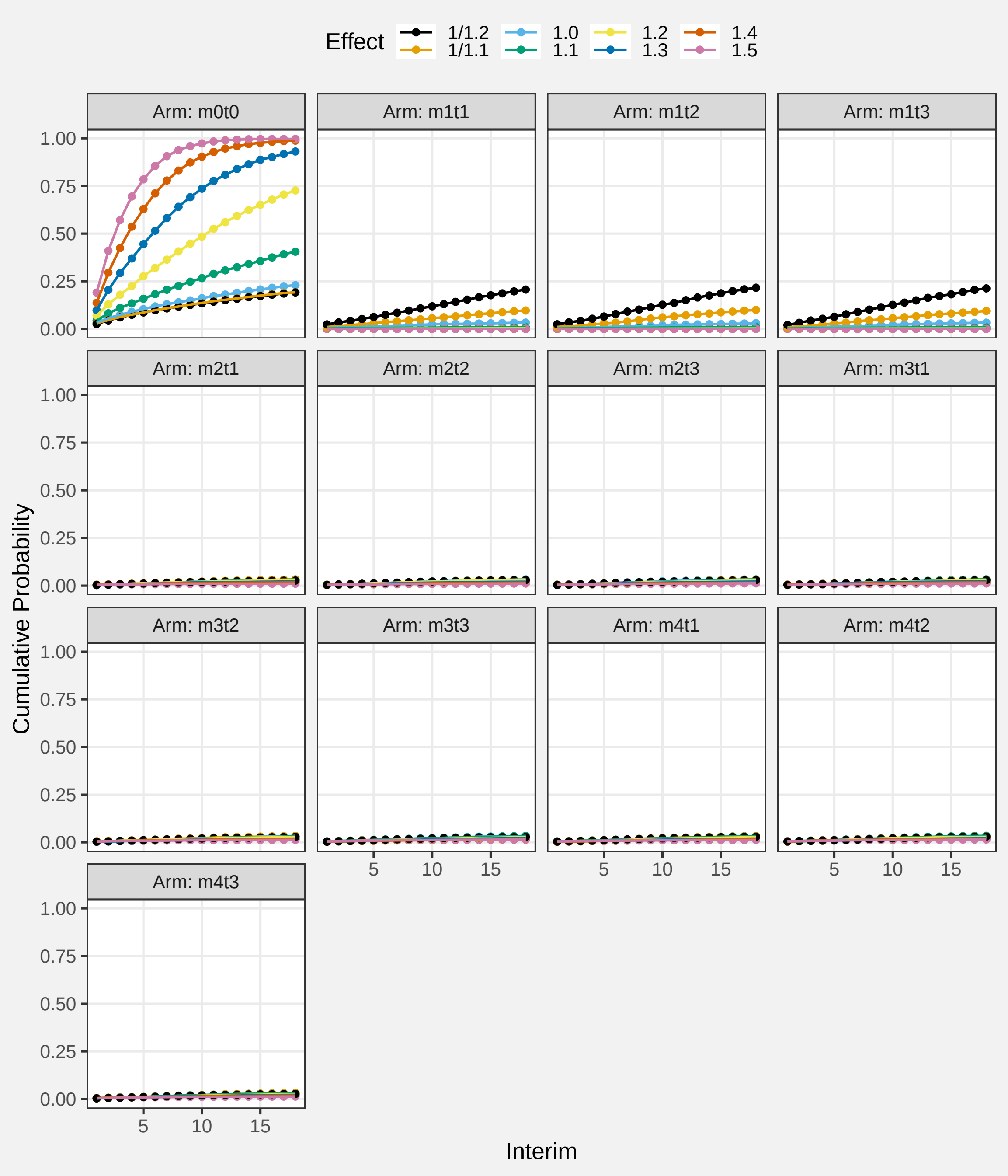

Figure 46: Decision times for dropping each intervention arm.

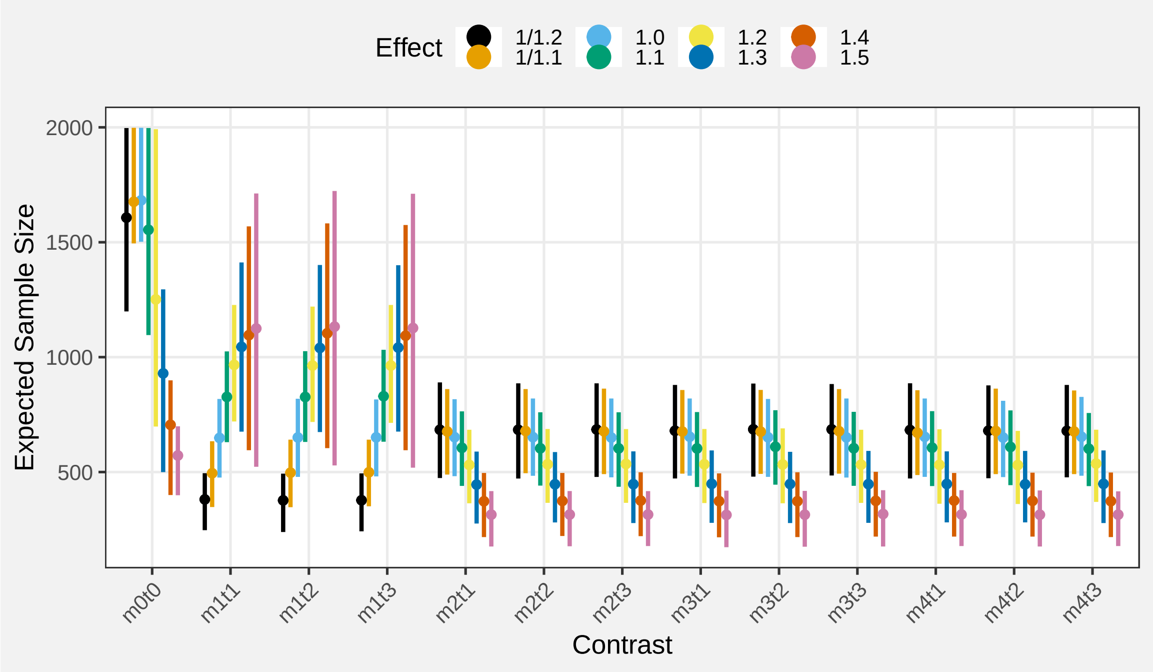

Figure 47: Expected sample size by arm.

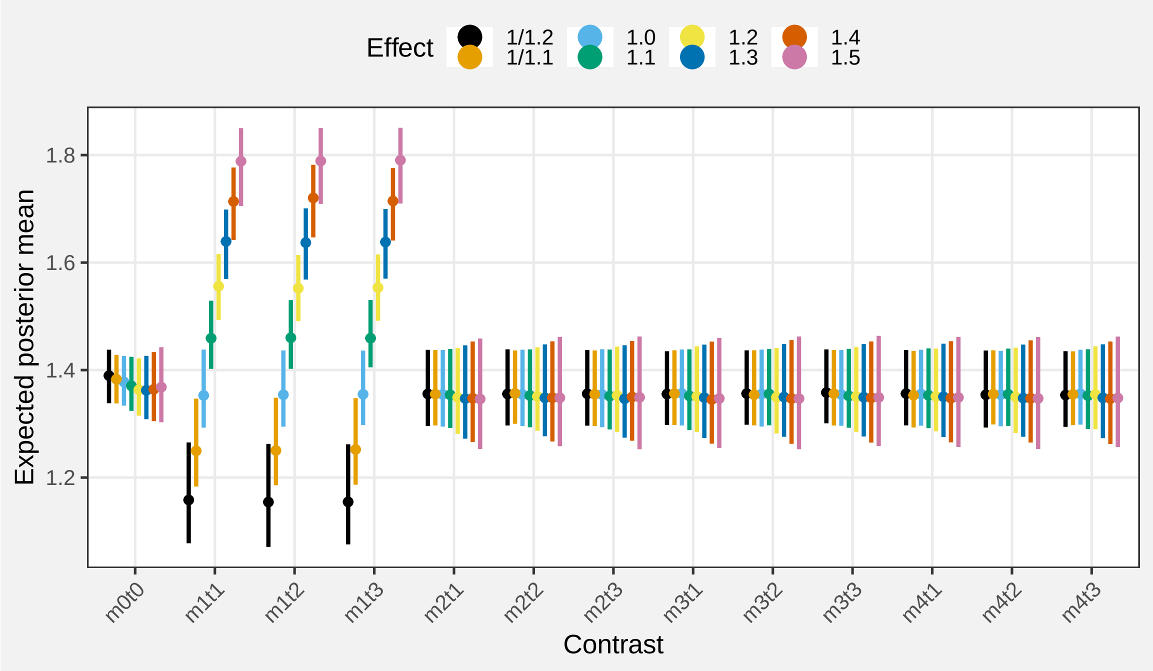

Figure 48: Expected posterior mean by arm.

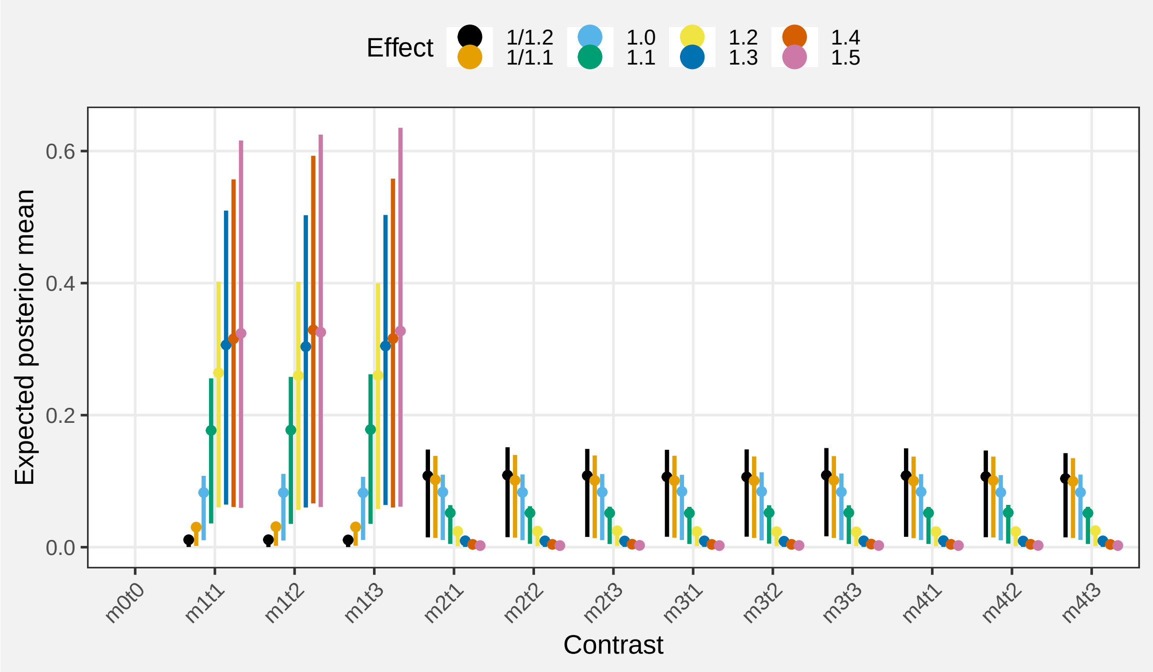

Figure 49: Expected posterior probability of superiority by arm.

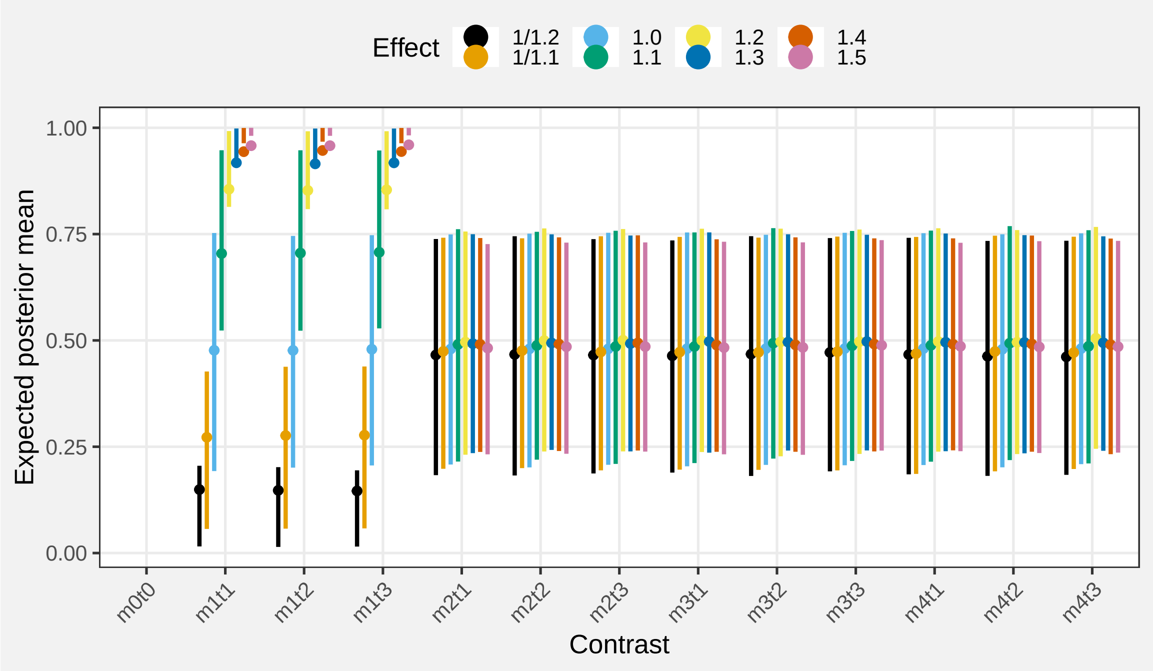

Figure 50: Expected posterior probability of effectiveness by arm.

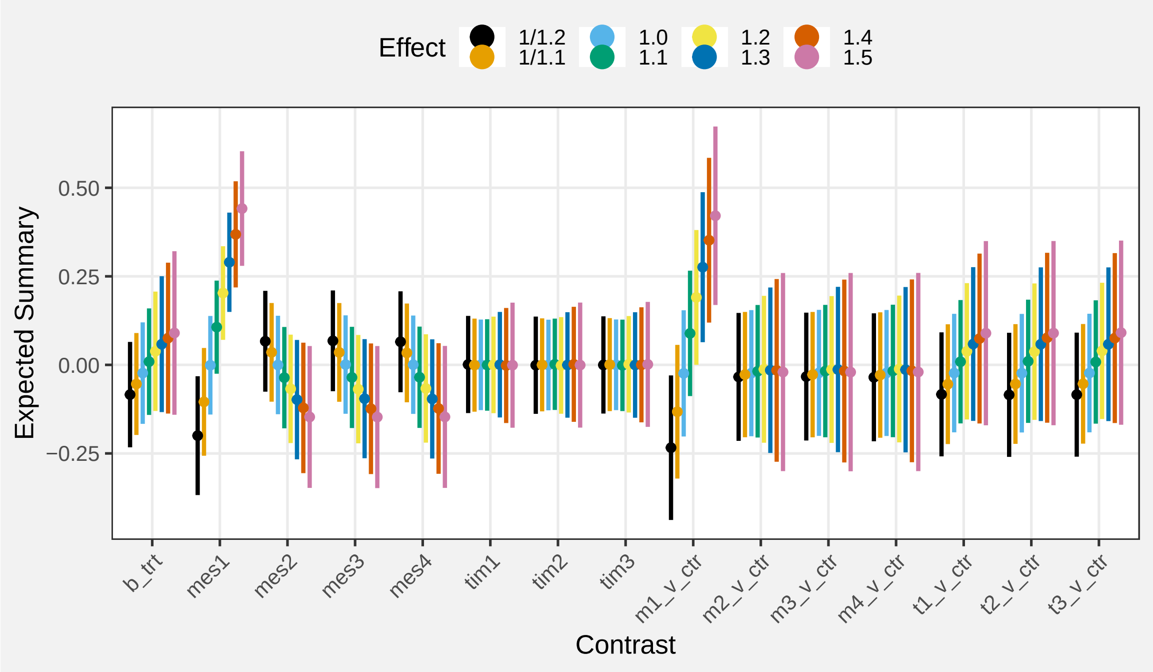

Figure 51: Expected posterior summary of contrasts.

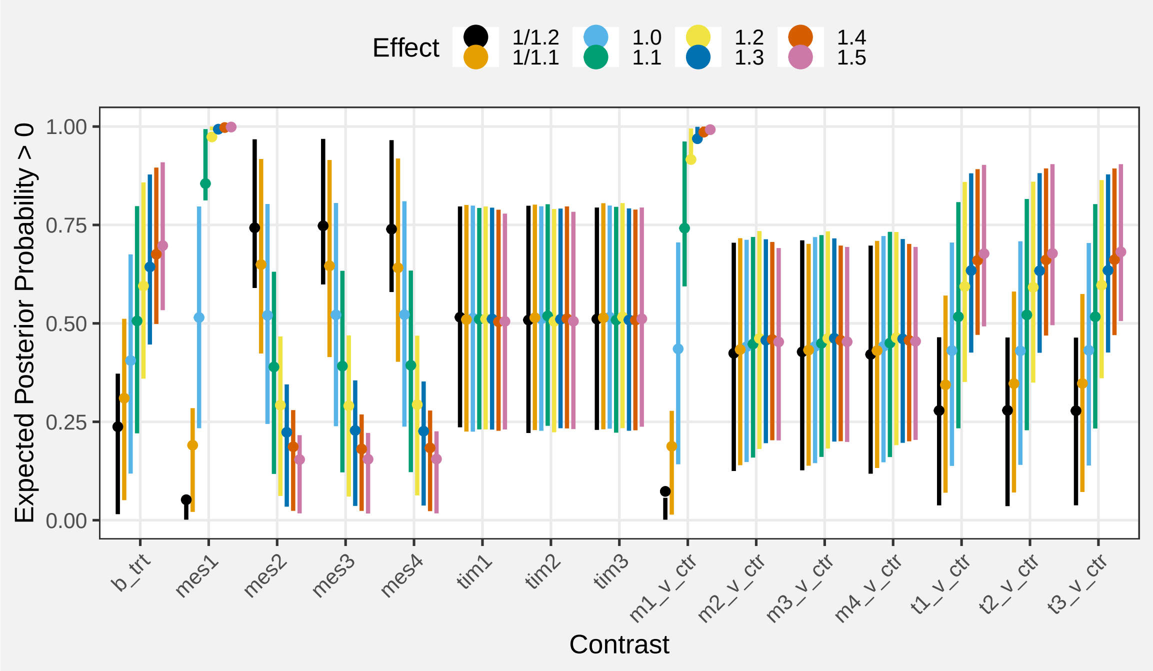

Figure 52: Expected posterior probability of contrast effect.

References

Currie, I.D., Durban, M., 2002. Flexible smoothing with P-splines: a unified approach. Statistical Modelling 2, 333–349.

Eilers, P.H., Marx, B.D., 1996. Flexible smoothing with B-splines and penalties. Statistical science 89–102.

Royston, P., Parmar, M.K., 2013. Restricted mean survival time: An alternative to the hazard ratio for the design and analysis of randomized trials with a time-to-event outcome. BMC medical research methodology 13, 152.

Royston, P., Parmar, M.K., 2011. The use of restricted mean survival time to estimate the treatment effect in randomized clinical trials when the proportional hazards assumption is in doubt. Statistics in medicine 30, 2409–2421.

Wand, M., Ormerod, J., 2008. On semiparametric regression with O'Sullivan penalized splines. Australian & New Zealand Journal of Statistics 50, 179–198.

SPIRIT
STANDARD PROTOCOL ITEMS: RECOMMENDATIONS FOR INTERVENTIONAL TRIALS

SPIRIT 2013 Checklist: Recommended items to address in a clinical trial protocol and related documents*

Section/item	ItemNo	Description	Comments
**Administrative information**			
Title	1	Descriptive title identifying the study design, population, interventions, and, if applicable, trial acronym	Page 3
Trial registration	2a	Trial identifier and registry name. If not yet registered, name of intended registry	Page 3
	2b	All items from the World Health Organization Trial Registration Data Set	N/A
Protocol version	3	Date and version identifier	Page 3
Funding	4	Sources and types of financial, material, and other support	Page 3
Roles and responsibilities	5a	Names, affiliations, and roles of protocol contributors	Page 3
	5b	Name and contact information for the trial sponsor	Page 4
	5c	Role of study sponsor and funders, if any, in study design; collection, management, analysis, and interpretation of data; writing of the report; and the decision to submit the report for publication, including whether they will have ultimate authority over any of these activities	Page 4
	5d	Composition, roles, and responsibilities of the coordinating centre, steering committee, endpoint adjudication committee, data management team, and other individuals or groups overseeing the trial, if applicable (see Item 21a for data monitoring committee)	Page 21
**Introduction**			
Background and rationale	6a	Description of research question and justification for undertaking the trial, including summary of relevant studies (published and unpublished) examining benefits and harms for each intervention	Page 4

	6b	Explanation for choice of comparators	Page 11
Objectives	7	Specific objectives or hypotheses	Page 6
Trial design	8	Description of trial design including type of trial (eg, parallel group, crossover, factorial, single group), allocation ratio, and framework (eg, superiority, equivalence, noninferiority, exploratory)	Page 7
**Methods: Participants, interventions, and outcomes**			
Study setting	9	Description of study settings (eg, community clinic, academic hospital) and list of countries where data will be collected. Reference to where list of study sites can be obtained	Page 9
Eligibility criteria	10	Inclusion and exclusion criteria for participants. If applicable, eligibility criteria for study centres and individuals who will perform the interventions (eg, surgeons, psychotherapists)	Page 9
Interventions	11a	Interventions for each group with sufficient detail to allow replication, including how and when they will be administered	Page 11
	11b	Criteria for discontinuing or modifying allocated interventions for a given trial participant (eg, drug dose change in response to harms, participant request, or improving/worsening disease)	Page 12
	11c	Strategies to improve adherence to intervention protocols, and any procedures for monitoring adherence (eg, drug tablet return, laboratory tests)	N/A – adherence to SMS reminders cannot be measured without contacting the parent to confirm it has been read but is assumed based on the reminder being sent.
	11d	Relevant concomitant care and interventions that are permitted or prohibited during the trial	Page 12

Outcomes	12	Primary, secondary, and other outcomes, including the specific measurement variable (eg, systolic blood pressure), analysis metric (eg, change from baseline, final value, time to event), method of aggregation (eg, median, proportion), and time point for each outcome. Explanation of the clinical relevance of chosen efficacy and harm outcomes is strongly recommended	Page 6
Participant timeline	13	Time schedule of enrolment, interventions (including any run-ins and washouts), assessments, and visits for participants. A schematic diagram is highly recommended (see Figure)	Page 12
Sample size	14	Estimated number of participants needed to achieve study objectives and how it was determined, including clinical and statistical assumptions supporting any sample size calculations	Page 15
Recruitment	15	Strategies for achieving adequate participant enrolment to reach target sample size	Page 16

Methods: Assignment of interventions (for controlled trials)

Allocation:

Sequence generation	16a	Method of generating the allocation sequence (eg, computer-generated random numbers), and list of any factors for stratification. To reduce predictability of a random sequence, details of any planned restriction (eg, blocking) should be provided in a separate document that is unavailable to those who enrol participants or assign interventions	Page 16
Allocation concealment mechanism	16b	Mechanism of implementing the allocation sequence (eg, central telephone; sequentially numbered, opaque, sealed envelopes), describing any steps to conceal the sequence until interventions are assigned	Page 16
Implementation	16c	Who will generate the allocation sequence, who will enrol participants, and who will assign participants to interventions	Page 16
Blinding (masking)	17a	Who will be blinded after assignment to interventions (eg, trial participants, care providers, outcome assessors, data analysts), and how	Page 17

	17b	If blinded, circumstances under which unblinding is permissible, and procedure for revealing a participant's allocated intervention during the trial	Page 17

Methods: Data collection, management, and analysis

Data collection methods	18a	Plans for assessment and collection of outcome, baseline, and other trial data, including any related processes to promote data quality (eg, duplicate measurements, training of assessors) and a description of study instruments (eg, questionnaires, laboratory tests) along with their reliability and validity, if known. Reference to where data collection forms can be found, if not in the protocol	Page 17
	18b	Plans to promote participant retention and complete follow-up, including list of any outcome data to be collected for participants who discontinue or deviate from intervention protocols	N/A - Plans to promote participant retention and complete follow up are not applicable to this study, as the study team will have no direct contact with participants due to the waiver of consent applied.
Data management	19	Plans for data entry, coding, security, and storage, including any related processes to promote data quality (eg, double data entry; range checks for data values). Reference to where details of data management procedures can be found, if not in the protocol	Page 18

Statistical methods	20a	Statistical methods for analysing primary and secondary outcomes. Reference to where other details of the statistical analysis plan can be found, if not in the protocol	Page 18
	20b	Methods for any additional analyses (eg, subgroup and adjusted analyses)	Page 20
	20c	Definition of analysis population relating to protocol non-adherence (eg, as randomised analysis), and any statistical methods to handle missing data (eg, multiple imputation)	Page 21
**Methods: Monitoring**			
Data monitoring	21a	Composition of data monitoring committee (DMC); summary of its role and reporting structure; statement of whether it is independent from the sponsor and competing interests; and reference to where further details about its charter can be found, if not in the protocol. Alternatively, an explanation of why a DMC is not needed	Page 22
	21b	Description of any interim analyses and stopping guidelines, including who will have access to these interim results and make the final decision to terminate the trial	Page 19
Harms	22	Plans for collecting, assessing, reporting, and managing solicited and spontaneously reported adverse events and other unintended effects of trial interventions or trial conduct	Page 22
Auditing	23	Frequency and procedures for auditing trial conduct, if any, and whether the process will be independent from investigators and the sponsor	Page 22
**Ethics and dissemination**			
Research ethics approval	24	Plans for seeking research ethics committee/institutional review board (REC/IRB) approval	Page 26
Protocol amendments	25	Plans for communicating important protocol modifications (eg, changes to eligibility criteria, outcomes, analyses) to relevant parties (eg, investigators, REC/IRBs, trial participants, trial registries, journals, regulators)	Page 22

Consent or assent	26a	Who will obtain informed consent or assent from potential trial participants or authorised surrogates, and how (see Item 32)	Page 10
	26b	Additional consent provisions for collection and use of participant data and biological specimens in ancillary studies, if applicable	N/A – This study will not use laboratory data
Confidentiality	27	How personal information about potential and enrolled participants will be collected, shared, and maintained in order to protect confidentiality before, during, and after the trial	Page 18
Declaration of interests	28	Financial and other competing interests for principal investigators for the overall trial and each study site	Page 27
Access to data	29	Statement of who will have access to the final trial dataset, and disclosure of contractual agreements that limit such access for investigators	Page 26
Ancillary and post-trial care	30	Provisions, if any, for ancillary and post-trial care, and for compensation to those who suffer harm from trial participation	Page 12
Dissemination policy	31a	Plans for investigators and sponsor to communicate trial results to participants, healthcare professionals, the public, and other relevant groups (eg, via publication, reporting in results databases, or other data sharing arrangements), including any publication restrictions	Page 23
	31b	Authorship eligibility guidelines and any intended use of professional writers	Page 26
	31c	Plans, if any, for granting public access to the full protocol, participant-level dataset, and statistical code	Page 21
**Appendices**			
Informed consent materials	32	Model consent form and other related documentation given to participants and authorised surrogates	Page 27

Biological specimens	33	Plans for collection, laboratory evaluation, and storage of biological specimens for genetic or molecular analysis in the current trial and for future use in ancillary studies, if applicable	N/A – This study will not use laboratory data.

*It is strongly recommended that this checklist be read in conjunction with the SPIRIT 2013 Explanation & Elaboration for important clarification on the items. Amendments to the protocol should be tracked and dated. The SPIRIT checklist is copyrighted by the SPIRIT Group under the Creative Commons "Attribution-NonCommercial-NoDerivs 3.0 Unported" license.
